# The method of loci in the context of psychological research: A systematic review and meta‐analysis

**DOI:** 10.1111/bjop.12799

**Published:** 2025-06-03

**Authors:** Jan Ondřej

**Affiliations:** ^1^ Department of Psychology Masaryk University Brno Czech Republic

**Keywords:** loci method, memory palaces, memory techniques, meta‐analysis, method of loci, mnemonics, systematic review

## Abstract

This systematic review and meta‐analysis aimed to evaluate (1) the effectiveness of the method of loci (MoL) in enhancing recall in adults, (2) its underlying cognitive mechanisms, and (3) its neurobiological correlates. Studies on adult populations were included from multiple databases. Risk of bias was assessed, and the GRADE approach and RoBMA_PSMA_ were used for qualitative and quantitative synthesis. The MoL showed strong evidence for a large effect on immediate serial recall compared with rehearsal (*d* = 0.88, 95% CI [0.47, 1.25], P(M|data) = 0.994, BF = 161.94) in adult populations. Its general effectiveness is supported by multiple cognitive mechanisms, all of which align with the levels of processing framework. Neuroimaging studies have shown consistent activation of brain regions primarily associated with spatial memory and navigation – namely, the hippocampus, parahippocampus, and retrosplenial cortex. MoL training also induces structural and functional brain changes. However, the evidence is rated as low to very low quality, mainly due to the high risk of bias and other limitations. The MoL is a powerful mnemonic for enhancing recall in adults, grounded in robust cognitive and neural mechanisms, though more rigorous studies are needed.

## BACKGROUND

### The method of loci

The method of loci (MoL), also known as the memory palaces or historically as ars memoriae, is widely recognized as a highly effective visuospatial mnemonic technique, first described by Simonides of Ceos in the fifth century BCE (Eysenck, 2020; Yates, 2015). The method is outlined in three ancient texts (Ad Herennium, 1954; Cicero, 1948; Quintilianus, 2002), which provide a structured approach. First, individuals select a space containing distinct locations. Second, they create vivid, often unusual, mental images of the items to be remembered, frequently associating these items with one another. Third, these images or associations are linked to specific locations within the chosen space. Finally, during recall, individuals typically retrieve items in a serial fashion by mentally navigating through the chosen space along a prescribed route, visualizing what was placed in each location in sequence. While there are additional rules and nuances, this forms the method's core. Historically, this mnemonic was closely associated with rhetoric, helping orators memorize lengthy speeches (Yates, 2015).

The MoL has a rich historical background, with an extensive body of literature spanning from ancient oratory handbooks to Renaissance manuals, and more recently, to contemporary best‐sellers on memory techniques. However, despite its enduring use and popularity, scientific research on the method remains relatively sparse. Formal investigations into the MoL began in 1968, and to date, only 83 experimental studies have been conducted. Most studies differ conceptually, with some topics frequently repeated and others significantly underrepresented. This suggests that the method remains relatively underexplored, and research is not cohesively united within the psychological context, highlighting the need to evaluate available knowledge comprehensively.

A single meta‐analysis (Twomey & Kroneisen, 2021) focused on the effectiveness of the MoL throughout its research history. Despite its promising results (*d* = 0.65, 95% CI [0.45, 0.85]), the meta‐analysis does not evaluate the risk of bias of randomized control trials (RCTs) with the Cochrane Risk of Bias 2 tool (RoB 2; Sterne et al., 2019), does not differentiate between young and adult populations, does not differentiate between conceptually different studies (e.g. Dalgleish et al., 2013), does not distinguish between different retention intervals, includes a limited number of studies, and does not explicitly say how the effect sizes for the meta‐analysis were chosen, despite some studies (e.g. Moè & De Beni, 2005) having more than 20 effect sizes possible to calculate.

Similarly, while one earlier meta‐analysis included a limited number of studies about the MoL (Verhaeghen et al., 1992), and several reviews mention it only marginally (Herrmann, 1987; Schneider et al., 2020; Vance et al., 2008; Yang et al., 2020), these studies are not systematic reviews, do not focus on the MoL primarily, and some of them need updating to incorporate recent findings.

This gap underscores the need for a comprehensive evaluation and synthesis of prior research. Such an effort can guide future studies and consolidate existing knowledge into a coherent body of evidence.

### The current study

This study is a systematic review and meta‐analysis summarizing the most relevant current evidence base concerning the MoL. Although we included a broad range of independent variables, our primary objective was to answer three questions: How effective is the MoL as a mnemonic device in enhancing recall? What cognitive mechanisms underlie its effectiveness? What are its neuropsychological correlates?

The systematic review follows the Preferred Reporting Items for Systematic Reviews and Meta‐Analyses (PRISMA) guidelines (Hutton et al., 2015) and PICO search strategies. We utilize the GRADE approach (Schünemann et al., 2013) to assess the quality of evidence in studies that do not report effect sizes and RoBMA_PSMA_ (Bartoš et al., 2022) for the quantitative evaluation of evidence of the method's effectiveness.

## METHODS

### Information sources

We searched the Web of Science, SCOPUS, EBSCOhost with Academic Search Ultimate, APA PsycArticles, APA PsycExtra, APA PsycInfo, and MEDLINE Complete databases included on 27 May 2024. For additional searches, we utilized previous reviews and meta‐analyses that included studies involving the MoL, although they did not primarily focus on it (Herrmann, 1987; Vance et al., 2008; Verhaeghen et al., 1992; Yang et al., 2020) except one (Twomey & Kroneisen, 2021) and also screened reference sections of cited articles for additional relevant articles. Keywords were searched in full text.

### Search strategy

Following the goal of the systematic review and meta‐analysis and the eligibility criteria, we used the following sets of keywords with the boolean operator ‘AND’ to search databases mentioned above: (1) method of loci OR loci method OR loci system OR memory palace OR memory palaces OR mind palace OR journey method OR art of memory OR ars memoriae OR ars memorativa OR spatial memory method OR method of places OR ancient memory system; (2) memory OR mnemonic technique OR memory strategies OR memorisation technique OR memory training OR memory aids OR mnemotechnic OR mnemonic OR memory enhancement OR memory improvement; (3) psychology OR cognitive psychology OR cognitive training OR neuropsychology.

### Selection process

To manage the study selection process, we imported the results into Rayyan, a web‐based application for systematic reviews (Ouzzani et al., 2016). After removing duplicates, we screened the remaining articles in three stages: title, abstract, and full‐text screening. At the abstract level, studies were excluded if they were clearly irrelevant (e.g. non‐empirical papers, animal studies, or studies unrelated to memory or MoL). During full‐text screening, studies were excluded if they did not meet the predefined inclusion criteria (see Eligibility Criteria).

### Eligibility criteria

Studies were included if they met the following criteria: (i) employed the method of loci, (ii) used an experimental design, and (iii) were published in an academic journal. *Language* restrictions were applied. Only studies published in English, Czech, or Slovak were considered for inclusion, but no methodological limitations were imposed to allow for a comprehensive overview of the available evidence. Specific criteria for each identified topic are outlined below.

#### The effectiveness of the method of loci in adults

We differentiate between younger (18–50 years) and older adult populations (51–85 years). We included studies that compared the effectiveness of the MoL to one or more of the following learning strategies: (1) rehearsal, defined as rote memorization through repetition without mnemonic aids; (2) free learning, where participants received no explicit learning instructions and used spontaneous, unguided strategies without structured mnemonics; and (3) alternative mnemonic techniques, including structured methods, such as the Pegword method, Story method, and Keyword method. Studies with varying recall types were also included, primarily using serial and free recall, although cued recall was also assessed.

##### The effectiveness of the method of loci in young adults

Eligible studies included healthy participants aged 18–50 years and compared the MoL with other learning strategies. Studies involving participants diagnosed with psychiatric disorders, such as schizophrenia (e.g. Sousa et al., 2021) or major depressive disorder (e.g. Dalgleish et al., 2013; Werner‐Seidler & Dalgleish, 2016), or studies that were thematically different were excluded (e.g. those that did not focus primarily on the effectiveness of the MoL in young adults, but rather on, for example, underlying cognitive mechanisms).

##### The effectiveness of the method of loci in older adults

Eligible studies included healthy participants aged 51–85 years. Studies were included if they examined the MoL compared with other learning strategies or investigated it within the context of longitudinal training. Studies involving participants with neuropsychological disorders, such as mild cognitive impairment or more advanced cognitive conditions, or studies that were thematically different were excluded (e.g. de Tournay‐Jetté et al. (2012), which focused on improvements in attention and memory among older adults following coronary artery bypass surgery).

#### The cognitive mechanisms underlying the effectiveness of the method of loci

Studies were included if they examined the cognitive mechanisms of the MoL in adults aged 18–85 years. This also encompassed studies involving participants with neurological or neuropsychological disorders (e.g. Canellopoulou & Richardson, 1998) to provide additional insight due to affected brain regions. Studies not thematically aligned were excluded.

#### The method of loci in the context of neuropsychology

Studies were eligible if they included adults aged 18–85 years, employed neuroimaging methods, such as fMRI, PET, or EEG to examine the neural mechanisms underlying the MoL. Studies that did not include neuroimaging methods were excluded.

### Data extraction

Data from each study were extracted and thoroughly verified to ensure accuracy and prevent errors. For studies published after 2010, if necessary data were unclear or unavailable, corresponding authors were contacted for clarification or additional information. Extracted details included study design, participant information (age, mean age, standard deviation, gender, location), intervention specifics (number of to‐be‐remember items, locations used, items per location, retention interval), comparison groups, missing data, and dependent variables. Descriptive statistics from randomized, non‐randomized, within‐subject, and other designs were used to calculate standardized mean differences for cross‐study comparisons. If data were unavailable, raw data were requested from the authors. Otherwise, effect sizes and confidence intervals were derived from inferential statistics.

### Statistical analysis and software

After data extraction, we calculated Cohen's *d* for each dependent variable using mean gains, pre‐test and post‐test standard deviations, and post‐test sample sizes from experimental and control groups (i.e. change from baseline effect size), employing a web‐based calculator (Wilson, n.d.). Effect sizes for RCTs, nRCTs, within‐subject, and other group designs were computed using mean gains, standard deviations, significance values, or standard error estimates unless specified otherwise. For studies with subscale scores or multiple measures, weighted mean effect sizes and confidence intervals were computed for simplicity using the formula for weighted means (Borenstein et al., 2021, pp. 65–66).

Data were analysed using JASP (v0.16.4; JASP Team, 2022) with the Robust Bayesian Meta‐Analysis with Publication Selection Model‐Averaging (RoBMA_PSMA_) method (Bartoš et al., 2022). Simulation studies employing RoBMA show it performs well under high heterogeneity, quantifies evidence for the absence of publication bias, and reduces the risk of confirmation bias by providing model‐averaged effect size estimates (Bartoš et al., 2022; Maier et al., 2023). Default prior probabilities were set: 18 of 36 models assumed the presence of the effect [P(M) = 0.50], 18 assumed heterogeneity [P(M) = 0.50], and 32 assumed publication bias [P(M) = 0.50]. To interpret Bayes factors (BF) and evaluate evidence for or against a hypothesis, we used guidelines by Bartoš et al. (2022): BF 1–3 (0.3–1), anecdotal evidence; BF 3–10 (0.1–0.3), moderate evidence; BF ≥10 (≤0.1), strong evidence; very strong evidence BF ≥30 (≤0.033). Effect sizes (Cohen's *d*) were categorized as: 0.00–0.19, negligible effect; 0.20–0.49, small; 0.50–0.79, moderate; ≥0.80, large (Cohen, 1988).

We also applied the GRADE approach (Schünemann et al., 2013) to assess the certainty of evidence, which ranges from high (further research is unlikely to change the estimate) to very low (the estimate is highly uncertain). Randomized controlled trials (RCTs) start as high‐certainty evidence, while observational studies start as low‐certainty, with ratings adjusted according to GRADE criteria. Downgrades were applied for risk of bias (e.g. poor randomization, lack of blinding, missing data), inconsistency (heterogeneity and unexplained variability), indirectness (differences in population, intervention, or outcomes), imprecision (wide confidence intervals, small sample sizes), and publication bias (suspected missing studies). Based on these criteria, a final GRADE rating was assigned to each outcome.

### Risk of bias assessment

We evaluated the risk of bias in the included studies using appropriate tools based on study design. For randomized controlled trials, we employed the Cochrane Risk of Bias 2 tool (RoB 2: Sterne et al., 2019), the Cochrane Risk of Bias for Non‐randomized Studies for Non‐randomized Controlled Trials (ROBINS‐I: Sterne et al., 2016) and the method quality of cross‐over studies involved in Cochrane Systematic Reviews (Ding et al., 2015) for a within‐subject experimental design. Other study designs were classified as having a high risk of bias.

Under established guidelines above, for assessing the risk of bias, a study's overall rating matches the highest risk level assigned to any of its domains. For instance, if a study is assessed across five domains and four are rated low risk while one is high, the study is deemed high risk overall. A high‐risk rating indicates that methodological flaws could plausibly distort the study's findings.

Using the RoB 2 framework (Sterne et al., 2019) and the randomization process (Domain 1) as an example, this domain is rated high risk if the allocation sequence is not adequately concealed or if baseline differences between groups suggest compromised randomization. It is rated low risk when the allocation sequence is appropriately generated and concealed, and any observed differences are likely due to chance.

For instance, a high‐risk scenario could occur if the allocation sequence is known in advance, potentially leading to participants with stronger baseline memory clustering in the intervention group. In contrast, a low‐risk scenario would involve a sealed, computer‐generated randomization procedure, preventing both participants and researchers from predicting group assignments and ensuring comparable baseline characteristics.

## RESULTS

### Selected studies

**FIGURE 1:**
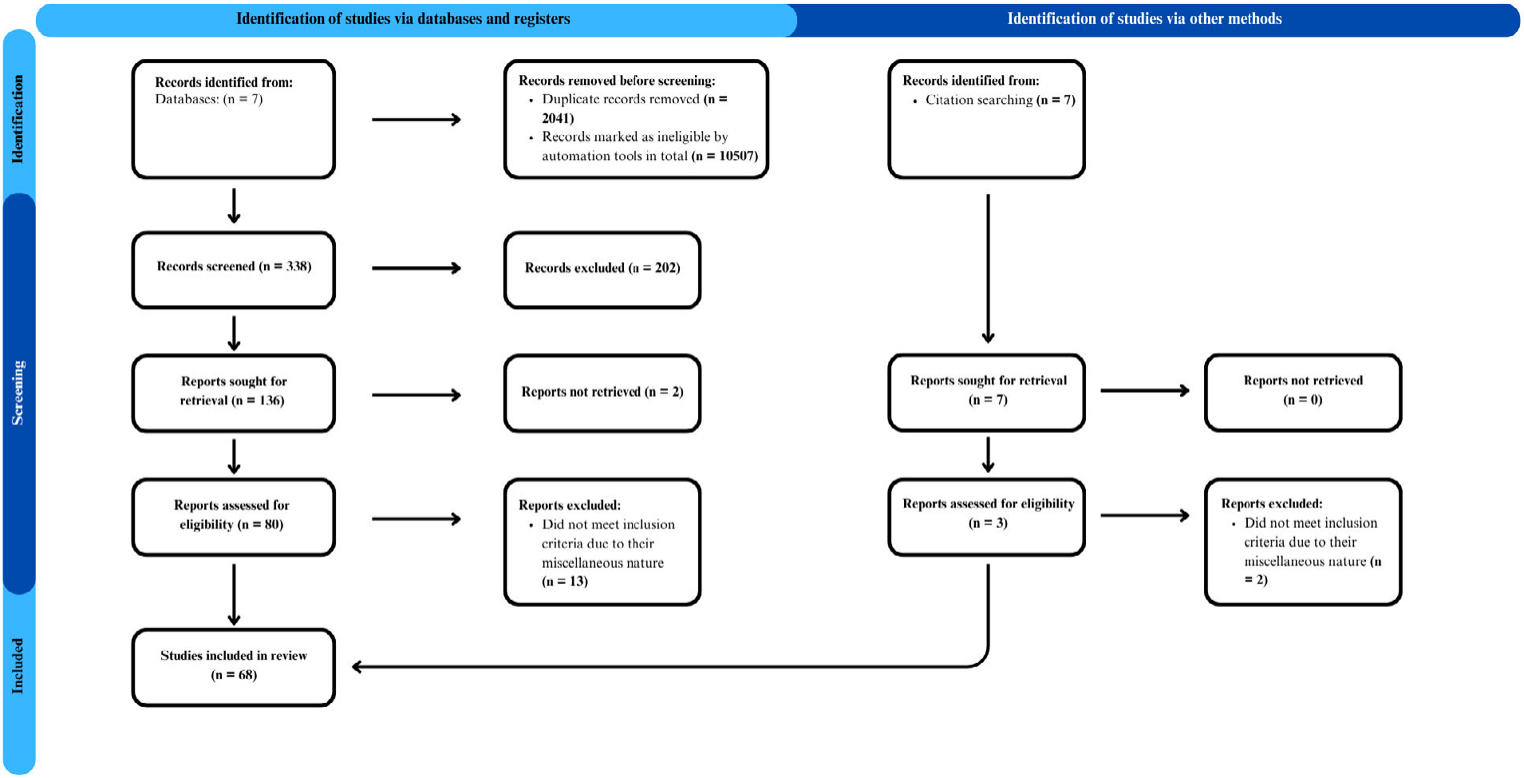
The PRISMA diagram.

We initially identified 12,886 studies through the search process, which was reduced to 10,845 after removing duplicates. Using the predefined keywords in the search strategy, we excluded 10,507 articles via Rayyan (Ouzzani et al., 2016), resulting in 338 articles for further screening. After reviewing the titles and abstracts, 202 studies were excluded, leaving 136 for full‐text screening. Ultimately, 83 studies were deemed eligible. However, not all of these studies directly addressed the main research questions due to their diverse or tangential focus. These studies are still reported to provide a comprehensive overview of the evidence base. The final number of studies included in the main analyses is 68 (Figure 1).

### Study characteristics

Studies included in the systematic review were grouped based on the thematic categories identified in the research aims. Several studies contributed to multiple categories and are marked as such in Tables 1 and 4.

#### The general effectiveness of the method of loci in young adults

The synthesis includes 37 experiments, comprising 24 randomized controlled trials (RCTs), 2 non‐randomized controlled trials (NRCTs), 5 within‐subject designs, and 6 studies using other designs. The qualitative evidence on effectiveness is based on 2604 participants, a total that includes data from Fassbender and Heiden (2006) and Moll and Sykes (2023).

Although these two studies did not fully meet the criteria for inclusion in the quantitative synthesis, they were retained in the qualitative analysis due to their thematic relevance. In both cases, only a small number of participants (3–5 individuals, representing 0.11–0.19% of the total sample) failed to meet the formal inclusion criteria.

Of the 37 experiments included, the mean age and age range were not consistently reported. And 8 studies provided the mean age but not the age range, 4 studies reported the age range without the mean, 15 studies did not report either, and only 10 studies included both. Based on the available data, the weighted mean age was 21.27 ± 3.25 years (17–44 years), or 21.47 ± 3.89 years (14–65 years) when including the two additional studies.

The study's participants were predominantly college undergraduate students from European and American universities. Table 1 summarizes the included studies.

**TABLE 1:** The effectiveness of the method of loci in young adults: characteristics of included studies and GRADE.

Citation	Participants description	Outcome	Number of to‐be‐remembered items	Number of locations	Number of to‐be‐remembered items per location	Nature locations	Retention interval
*Randomized control trials*							
Bass and Oswald (2014)	94 (%f = 64) undergraduate psychology students from California State University, Fresno, each receiving one credit towards course requirements, aged NI (M = 19 ± NI), United States	FR	5 sets of 5 similar concrete words	25	1; 2; 3; 4; 5 (technically 1)	S	I
Bower and Reitman (1972)	30 (%f = NI) Stanford University undergraduates paid for their effort, aged NI (M = NI ± NI), United States	SR	5 lists of 20 concrete words	20	≈1–5	S	I, D, 1W
Carlson et al. (1976)	122; 76 after exclusion (%f = NI) college students from four introductory psychology classes at Georgia Southern College, aged NI (M = NI ± NI), United States	FR	4 lists of 20 concrete noun	20	≈1–4	E	I, 1D
Cornoldi and De Beni (1991) Experiment 1	105 (%f = 86.7%) university students attending introductory courses in psychology, aged NI (M = NI ± NI), Italy	SR	Complex text	20	NI	S	I
Cornoldi and De Beni (1991) Experiment 2	125 (%f = NI) university students and senior high school students, aged 17–20 (M = NI ± NI), Italy	SR	Complex text	20	NI	S	I, 1W
Crovitz (1971)	70 (%f = NI) Duke University undergraduates who served as part of a course requirement, aged NI (M = NI ± NI), United States	SR	32 concrete words	32; 16; 8; 4; 2; 1; 0	32; 16; 8; 4; 2; 1	E	I
De Beni and Cornoldi (1985) Experiment 1	28 (%f = NI) university students attending an introductory psychology course, aged NI (M = NI ± NI), Italy	SR	20 triplets (60) high‐imagery, concrete nouns	20	3 (triplets)	E	I
De Beni and Cornoldi (1985) Experiment 2	56 (%f = NI) university students attending an introductory psychology course, aged NI (M = NI ± NI), Italy	SR and REC	20 triplets (60) high‐imagery, concrete nouns	20	3 (triplets)	E	I, 1W
De Beni and Cornoldi (1988)	56 (%f = NI) university students attending an introductory psychology course, aged NI (M = NI ± NI), with adequate training in the use of the mnemonic, Italy	SR	3 lists of 20 (abstract, concrete)	NI	NI	≈S	I
De Beni et al. (1997) Experiment 1	125 (%f = 49,6%) senior high school students, aged 17–20 (M = 19 ± NI), Italy	SR	Complex text	20	NI	S	I
De Beni et al. (1997) Experiment 2	34 (%f = 82.35%) university students, aged NI (M = 20 ± NI), Italy	SR	Complex text	20	NI	S	I
De Beni et al. (1997) Experiment 3	32 (%f = 82.35%) psychology students, aged NI (M = 20 ± NI), Italy	SR	Complex text	NI	NI	≈S	I, 1W
Groninger (1971)	72 initially, 46 after 1 week, 38 after 5 weeks (%f = NI) Students of the University of Maryland who served in this study as a substitute for other course work, aged NI, (M = NI ± NI), United States	SR and REC	25 high‐imagery words	25	1	S	1W, 5W
Kluger et al. (2022) Experiment 1	221 (%f = NI) undergraduate psychology students recruited from the introductory psychology research participation pool in partial fulfilment of course requirements, aged NI (M = 19.58 ± NI), Canada	SR and FR	8 lists of 10 nouns	10	1–8	S	I
Kroneisen and Makerud (2017) Experiment 1	48 (%f = 93.75) university students from the University of Mannheim; received course credit for compensation, aged NI (M = 20.37 ± 1.95) Germany	FR	30 high‐low imageability words	NI	NI	S	I
Kroneisen and Makerud (2017) Experiment 2	52 (%f = 80.76) university students from the University of Mannheim received course credit for compensation, aged 18–26 (M = 20.92 ± 1.67), Germany	FR	28 high‐imageability, high‐survival relevance	NI	NI	S	I
Legge et al. (2012)	142 (%f = 61.97) undergraduate students for partial credit in an introductory psychology course at the University of Alberta, aged 17–27 (M = 19.3 ± 1.69), Canada	SR and FR	10 lists of 11 high‐low imaginability words	7, 8 and 8	NI	S and E	I
Massen and Vaterrodt‐Plünnecke (2006) Experiment 1	132 (%f = 37.87) undergraduate students at the University of Bonn, aged 20–40 (M = NI ± NI), Germany	SR	3 lists of 20 (similar, dissimilar)	20	1; 2; 3	S	I
Massen and Vaterrodt‐Plünnecke (2006) Experiment 2	108 (%f = 63.88) undergraduate students at the University of Bonn, aged 18–44 (M = NI ± NI), Germany	SR	3 lists of 20 (similar, dissimilar)	20	1; 2; 3	S	I
Moè and De Beni (2005)	90 (%f = 61.11%), high‐school students, aged NI (M = 18.21 ± 1.04), Italy	SR	Complex text	10	1–2	S	I
Qureshi et al. (2014)	78 (%f = NI), second‐year medical students at Rawal Medical College, Rawal Institute of Health Sciences aged NI (M = NI ± NI), Pakistan	(MCQs)	Complex university material	3MP	NI	S	≈I–D
Roediger (1980)	150 (%f = NI) undergraduate students from Purdue University who served in partial fulfilment of a course requirement, aged NI (M = NI ± NI), United States	SR and FR	3 lists of high‐imagery nouns	20	≈1–3	S	I, 1D
Wang and Thomas (2000)	202 (%f = NI) students in introductory psychology class, aged NI (M = NI ± NI), United States	FR	20 concrete words	20	NI	≈S	I, 2D
Weinstein et al. (1981)	100 (%f = NI) students from the introductory educational psychology course, aged NI (M = NI ± NI), United States	SR	20	20	1	S	I
*Within‐subject designs*							
Bouffard et al. (2018) Experiment 1	31 (%f = 77.42) undergraduate psychology students, all participants were compensated with course credit for participating, aged 18–26 (M = 20.92 ± 1.67), United States	FR	10 concrete nouns	10	1	S	I
Bouffard et al. (2018) Experiment 2	31 (%f = 61.29) undergraduate psychology students from the University of California were compensated with course credit for participating, aged 18–29 (M = 20.72 ± 2.50), United States	FR	10 concrete nouns	10	1	S	I
Fellner et al. (2016)^a^	For EEG 21 (%f = 57.14) healthy participants, aged 18–24 (M = 20.19 ± NI) and for fMRI 23 (%f = 65.21) healthy participants, aged 18–36 (M = 22.9 ± NI), Germany	SR	6 lists of 20 words	20	1–6	S	I
Liu et al. (2022)^a^	29 (%f = 65.51) healthy participants, aged 18–24 (M = 20.3 ± NI)	SR	2 lists of 30 words	10	1–6	E	I
Saraiva et al. (2016)	56 (%f = 89.2) volunteer undergraduate students from the University of Minho received course credits for their participation, aged NI (M = 21.18 ± 3.85), Portugal	SR	30 concrete words	30	1	S + E	I
*Non randomized trials*							
Dresler et al. (2017)^a^	23 (%f = 39.13) Memory athletes at the Top‐50 of the memory sports world rankings, aged NI (M = 28 ± 8.6) matched with NI (%NI = NI) control participants recruited among gifted students of academic foundations and members of the high‐IQ society Mensa, aged NI (M = NI ± NI) and 51 (%f = 0) participants without any prior experience in mnemonic strategies, aged NI (M = 24 ± 3), Germany	SR	NI, 72	NI, NI (3 memory palaces)	NI, NI	NI, S + E	I
Mallow et al. (2015)^a^	11 (%f = 27.27) mnemonists highly trained in the MoL and with proven abilities in public competitions of memory performance, aged NI (M = 30.8 ± NI) and 11 (%f = 45.45) control participants naïve to the MoL, aged NI (M = 26.3 ± NI), Germany	SR	8 block of 40 digits (320 in total)	NI	NI	S	I
*Other designs*							
Fassbender and Heiden (2006)	15 (%f = NI) NI (only students), aged 21–51 (M = NI ± NI)	SR	10 concrete words	10	1	E	I
Kliegl et al. (1987)	2 (%f = 0) young adult students, aged 19–23 (M = 21 ± NI), Germany	SR and FR	Digits	NI	NI	S	I
McCabe (2015)	(probably 57, uses 39–40 participants in analysis) reports 30 (%f = NI) undergraduate students enrolled in a Human Learning and Memory course at Goucher College, aged NI (M = NI ± NI), United States	SR and FR	12 concrete words	12	1	S	I
Moll and Sykes (2023)	11 (%f = 27.27) post‐secondary degree college students, aged 14–65 (M = 35.9 ± 14.9), Canada	SR and FR	30 high‐imageability words	NI	NI	E	I
Ross and Lawrence (1968) Experiment 1	1 (%f = 0) the junior author, aged NA (M = NI ± NI), Australia	SR and FR	50	52	1	S	I, 1D, NI
Ross and Lawrence (1968) Experiment 2	5 (%f = NI) senior students in psychology, aged NI (M = NI ± NI), Australia	SR	40 concrete (abstract, nouns)	52	1	S	I, 4D, 10D
GRADE	Very low: The available evidence regarding the loci method's effectiveness in the young adults is highly uncertain due to significant limitations such as the risk of bias, inconsistency, imprecision, or indirectness. Any effect estimate is very uncertain, and further research is required to provide more reliable evidence						

*Note*: SR = serial recall, FR = free recall, NI = no information, ≈ = approximately, E = experimentator‐generated locations, S = subject‐generated locations, S + E = subject‐generated locations but controlled by experimentator, I = immediate recall, D = days, W = week, ; = intentionally placed more than one to‐be‐remember item per location.

^a^ Studies contributing to the neuropsychological topic.

#### The general effectiveness of the method of loci in older adults

The synthesis includes 18 experiments, where 4 are RCTs, 10 NRCTs, and 4 other designs. The qualitative evidence for the effectiveness in older adults is based on 2421 participants. Not all studies reported age‐related data. Three experiments did not provide age ranges. The weighted mean age was 61.26 ± 6.49 years (17–88 years). The participants were predominantly elderly volunteers from the United States and Europe. Table 2 summarizes the included studies.

**TABLE 2:** The effectiveness of the method of loci in older adults: characteristics of included studies and GRADE.

Citation	Participants description	Outcome	Number of to‐be‐remembered items	Number of locations	Number of to‐be‐remembered items per location	Nature locations	Retention interval
*Randomized control trials*							
Boller et al. (2021)	40 (%f = 82.5) independent community‐dwelling older adults, aged NI (50+) from which 20 were in VR+ group (M = 66.0 ± 7.64), and 20 in VR− group (M = 68.6 ± 7.27), Canada	FR	2 lists of 12 words	12	≈1−2	S, E	I
Gross et al. (2014)	1401 (%f = 75.02) ACTIVE study participants of community‐living adults from which 174 were AVLT skippers, aged NI (M = 71.8 ± 5.2), AVLT non‐skippers, aged NI (M = 74.1 ± 6.2) and control group, aged NI (M = 74.0 ± 6.0), United States	SR	AVLT (≈15)	NI	NI	NI	≈I
Hill et al. (1991)	71 (%f = 39) elderly volunteers, aged 60−83 (M = 70.4 ± 6.2), United States	SR	26 concrete words	25	NI (≈1)	S	I, 1H, 3D
Yesavage and Rose (1984)	37 (%f = 78) middle‐level managers of the industrial firms belonging to a large retirement fund, aged 60−73 (M = 66.15 ± 4.61), France	SR	18 concrete words	20	1	S	I, 20 M
*Non‐randomized trials*							
Baltes and Kliegl (1992)	35 (NI) of the same 37 (%f = 48.64) participants, from which 18 were university students, aged 19−29 (M = 23.9 ± NI) and 19 older adults, aged 65−80 (M = 71.7 ± NI), Germany (Kliegl et al., 1989; Experiment 2)	SR	≈72+ lists of 30 words	NI, (≈30)	≈36−72 (without training)	E	I
Brooks et al. (1999)	268 (%f = 71.64) elderly volunteers, aged 55−88 (M = 68.81 ± 7.07), United States	SR	16 (abstract, concrete) words	16	1	S	I
Kliegl et al. (1989) Experiment 1	24 participants, from which 20 (%f = NI) were old adults, aged 65−83 (M = 71.7 ± NI) and 4 (%f = NI) university students, aged 20−24 (M = 22.8 ± NI), Germany	SR	3 lists of 40 words	40	≈1−3	E	I
Kliegl et al. (1989) Experiment 2	37 (%f = 48.64) participants, from which 18 were university students, aged 19−29 (M = 23.9 ± NI) and 19 older adults, aged 65−80 (M = 71.7 ± NI), Germany	SR	6 lists of 40 words	30	≈1−6	E	I
Kliegl et al. (1990)	37 participants, from which 18 (%f = 38.9) were university students, aged 19−29 (M = 23.9 ± NI) and 19 older adults, aged 65−80 (M = 71.7 ± NI), Germany	SR	≈36 lists of 30 words	30	≈12−36	E	I
Lindenberger et al. (1992)	24 (%f = 50) participants, from which 6 older graphic designers, aged 64−81 (M = 69.9 ± NI) 6 older control adults, aged 64−80 (M = 70.5 ± NI), 6 younger graphic design students, aged 22−24 (M = 23.0 ± NI) and 6 normal younger control students, aged 21−24 (M = 22.6 ± NI), Germany	SR	48 lists of 20 words	20	≈12−48 (without training)	E	I
Rebok and Balcerak (1989)	93 participants, from which 48 (%f = 81.25) of young adults from the introductory psychology subject pool, aged 17−19 (M = 18.08 ± NI) and 45 (%f = 85.71) old adults from a senior citizens' community organization in Rochester, aged 60−78 (M = 67.84 ± NI), United States	SR	12 concrete words	12	NI, (≈1)	S	I
Robertson‐Tchabo et al. (1976) Experiment 2	30 (%f = 70) elderly volunteers from senior citizens centres, aged 60−84 (M = 71.02 ± NI), United States	FR	16 concrete words	16	1	S	I
Verhaeghen and Marcoen (1996)	139 participants, from which 76 (%f = 53) elderly volunteers, aged 60−87 (M = 23.0 ± 5.1) and 63 (%f = 51) college undergraduates students, aged 18−25 (M = 18.7 ± 1.3), Belgium	SR	3 lists of 25 concrete words	25	≈1−3	NI (≈S)	I
Rose and Yesavage (1983)	67 (%f = 35.82) middle‐level managers of the industrial firm and students, aged 21−67 (M = 50.35 ± 8.57); young group (M = 27.8 ± 5.8), middle‐aged group (M = 53.3 ± 5.6), older group (M = 61.4 ± 1.5), France	SR	18 (abstract, concrete)	NI (≈18)	NI, (≈1)	S	I
*Other designs*							
Anschutz et al. (1985)	10 (%f = 80) elderly volunteers from apartment complexes for older adults, aged 63−82, (M = 71 ± NI), United States	FR	12 words	12	1	S	I, 4W
Anschutz et al. (1987)	9 (%f = 77.8) elderly volunteers from apartment complexes for older adults, aged 66−85, (M = 73.5 ± NI), United States	FR, REC	12 words	12	1	S	3Y, I
Robertson‐Tchabo et al. (1976) Experiment 1	5 (%f = 75) elderly volunteers, aged 60−67 (M = 69.3 ± NI), United States	FR	16 concrete words	16	1	S	I
Weintruab‐Youdkes et al. (2015)	22 (%f = 86.33), elderly volunteers, aged NI (M = 80.6 ± 6.4), Israel	FR	NI	NI	NI	S	NA
GRADE	Very low: The available evidence regarding the loci method's effectiveness in the elderly is highly uncertain due to significant limitations such as the risk of bias, inconsistency, imprecision, or indirectness. Any effect estimate is very uncertain, and further research is required to provide more reliable evidence						

*Note*: SR = serial recall, FR = free recall, NI = no information, ≈ = approximately, E = experimentator‐generated locations, S = subject‐generated locations, S + E = subject‐generated locations but controlled by experimentator, I = immediate recall, D = days, M = minutes, W = week, Y = year, NA = not applicable, ; = intentionally placed more than one to‐be‐remember item per location.

#### The cognitive mechanisms underlying the effectiveness of the method of loci

The synthesis includes 15 experiments, with 7 RCTs, 1 NRCT, 5 within‐subject designs, and 2 other designs. The qualitative evidence is based on 1220 participants. Not all studies reported age‐related data. Nine studies did not report either the mean age or age range, while six studies reported both. Among those that did, the weighted mean age was 26.45 ± 10.68 years (17–44 years). Most participants were undergraduate students from European and American universities. Table 3 summarizes the included studies.

**TABLE 3:** The cognitive mechanisms underlying the effectiveness of the method of loci: characteristics of included studies and GRADE.

Citation	Participants description	Outcome	Number of to‐be‐remembered items	Number of locations	Number of to‐be‐remembered items per location	Nature locations	Retention interval
*Randomized control trials*							
Bellezza and Reddy (1978)	72 (%f = NI) volunteers from introductory courses in psychology, subjects received extra course credit for their participation in the experiment, aged NI (M = NI ± NI), United States	SR	20 concrete words	20	1	S + E	I
Blunt and VanArsdall (2021) Experiment 1	154 (%f = 61) participants recruited online via a Human Intelligence Task (HIT) posted on Amazon Mechanical Turk, aged NI (M = NI ± NI), United States	FR	30 words (animate, inanimate)	NI	NI (pairs)	S	I
Blunt and VanArsdall (2021) Experiment 2	200 (%f = 58.5) participants recruited online via a Human Intelligence Task (HIT) posted on Amazon Mechanical Turk, aged 19−67 (M = 35 ± 10.8), United States	FR	30 words (animate, inanimate)	NI	NI	S	I
Kemp and Van der Krogt (1985) Experiment 1	77 (%f = NI) students in the 1st‐year psychology course, aged NI (M = NI ± NI), New Zealand	SR	25 concrete words	25	1	E	I
Massen et al. (2009) Experiment 1	40 (%f = 57.5) students from the University of Bonn campus, aged 20−47 (M = 26 ± NI), Germany	SR	3 lists of 20 grocery items	20	1	S	I
Massen et al. (2009) Experiment 2	120 (%f = 60.83) mostly students recruited from the University of Bonn campus, aged 18−40 (M = 23.2 ± NI), Germany	SR	4 lists of 20 concrete words	20	1	S	I
Reggente et al. (2020)	60 (%f = 50) participants, aged 18−27 (M = 21 ± 2.25), Switzerland	SR, FR	3 sets of 15‐3D objects	15 per 3 environments (45 in total)	1	E	I
*Non‐randomized trials*							
Kemp and Van der Krogt (1985) Experiment 2	164 (%f = NI) students in the 1st‐year psychology course, aged NI (M = NI ± NI), New Zealand	SR	25 concrete words	25	1	E	I
*Within‐subject designs*							
Canellopoulou and Richardson (1998)	50 (%f = 62) patients with mental sclerosis, aged 35−76 (M = 51 ± NI), UK	FR	2 × 8 groceries	NI (≈8−16)	NI (≈1−2)	S + E	I
Caplan et al. (2019)	173 (%f = 66.47) students in introductory psychology courses remunerated with up to CAD $3.25 depending on their compliance level, aged 17−35 (M = 19.5 ± 2.5), Canada	SR, FR	5 lists of 11 concrete words	Three environments, 7, 8 and 8 locations	NI	E	I
Lea (1975) Experiment 1	12 (%f = NI) students participating in fulfilment of a class requirement, aged NI (M = NI ± NI), United States	SR	12 concrete words	12	1	E	I
Lea (1975) Experiment 2	24 (%f = NI) students participating in fulfilment of a class requirement, aged NI (M = NI ± NI), United States	SR	12 concrete words	12	1	E	I
Lea (1975) Experiment 3	12 (%f = NI) students participating in fulfilment of a class requirement, aged NI (M = NI ± NI), United States	SR	12 concrete words	12	1	E	I
*Other designs*							
Briggs et al. (1970)	50 (%f = NI) undergraduate students, aged NI (M = NI ± NI), England	SR	20 concrete words	20	1	E	I
Crovitz (1969)	12 (%f = NI) undergraduate students, aged NI (M = NI ± NI), England	SR	40 concrete words	20	2 (pairs)	E	I
GRADE	Very low: The available evidence regarding the cognitive mechanisms underlying the loci method's effectiveness in the elderly is highly uncertain due to significant limitations such as the risk of bias, inconsistency, imprecision, or indirectness. Any effect estimate is very uncertain, and further research is required to provide more reliable evidence						

*Note*: SR = serial recall, FR = free recall, NI = no information, ≈ = approximately, E = experimentator‐generated locations, S = subject‐generated locations, S + E = subject‐generated locations but controlled by experimentator, I = immediate recall.

#### The method of loci in the context of neuropsychology

The synthesis includes 15 experiments, comprising 1 RCT, 9 NRCTs, 3 within‐subject designs, and 2 other designs. The qualitative evidence is based on 710 participants. Not all studies reported age‐related data. Four studies did not report the age range, and two did not provide the mean age. Among the studies that did, the weighted mean age was 26.1 ± 33.4 years (18–80 years). Participants comprised memory experts, gifted controls (e.g. Mensa members), older adults (some with subjective cognitive decline), and young healthy adults or students, making the population rather diverse.

**TABLE 4:** The method of loci in the context of neuropsychology: characteristics of included studies and GRADE.

Citation	Participants description	Neuroimaging method	Number of to‐be‐remembered items	Number of locations	Number of to‐be‐remembered items per location	Nature locations
*Non‐randomized trials*						
Belleville et al. (2023)	Full neuroimaging and behavioural assessment: 29 (%f = 83) healthy seniors but with subjective cognitive decline, aged NI (M = 67 ± 8.44) and with only behavioural assessment: 40 (%f = 82.5) healthy seniors but with subjective cognitive decline, aged NI (M = 67 ± 7.49), Canada	fMRI	6 lists of 12	12	1−6	S
Dresler et al. (2017)^a^	23 (%f = 39.13) Memory athletes of the Top‐50 of the memory sports world rankings, aged NI (M = 28 ± 8.6) matched with NI (23) (%NI = NI) control participants recruited among gifted students of academic foundations and members of the high‐IQ society Mensa, aged NI (M = NI ± NI) and 51 (%f = 0) participants without any prior experience in mnemonic strategies, aged NI (M = 24 ± 3), Germany	fMRI	NI, 72	NI, NI (3 memory palaces)	NI, NI	NI, S + E
Engvig et al. (2010)	22 (%f = 54.54) healthy volunteers recruited through a local newspaper ad as an experimental group, aged 42−76 (M = 61.3 ± 9.4) and 20 (%f = 55) healthy volunteers recruited through a local newspaper ad as a control group, aged 42−77 (M = 60.3 ± 9.1), Norway	MRI	NA	NA	NA	S
Maguire et al. (2003)	10 (%f = 0) superior mnemonists, aged 22−53 (M = 33.9 ± 9.33) and 10 (%f = 0) control participants, aged 20−46 (M = 31.1 ± 7.9), UK	fMRI	NI, digits, face, snowflakes	NI	NI	NI, ≈S
Mallow et al. (2015)^a^	11 (%f = 27.27) mnemonists highly trained in the MoL and with proven abilities in public competitions of memory performance, aged NI (M = 30.8 ± NI) and 11 (%f = 45.45) control participants naïve to the MoL, aged NI (M = 26.3 ± NI), Germany	fMRI	320 digits	NI	NI	S
Müller et al. (2018)	23 (%f = 39.13) memory athletes of the Top‐50 of the memory sports world rankings, aged 19−51 (M = 27.8 ± NI) and 23 (%f = 39.13) gifted students of academic foundations and members of the Mensa Society, aged 20−53 (M = 28.1 ± NI), Germany	fMRI	NA	NA	NA	NA
Nyberg et al. (2003)	8 (%f = 40) young adults, aged NI (M = 25.75 ± 2.6), 16 older adults: 8 (%f = 100) facilitated old, aged NI (M = 69.75 ± 2.96), 8 (%f = 33.3) unimproved old, aged NI (M = 67.75 ± 1.98), Sweden	PET	2 lists of 18 words	18	1−2	S
Wagner et al. (2021) Experiment 2	51 (%f = 0) students at the University of Munich, aged 20−30 (M = 24 ± 3), Germany	fMRI	72	NI	NI	S + E
Wagner et al. (2021) Experiment 1	17 (%f = 47.05) experts in using the method of loci ranking among the world's top 50 in memory sports, aged 19−32 (M = 24.6 ± 4.3) and 16 (%f = 43.75) matched control participants that were gifted students of academic foundations and members of the high‐IQ society Mensa, aged 20−35 (M = 25.4 ± 3.9), Germany	fMRI	72	NI	NI	NI
*Randomized control trial*						
de Lange et al. (2018)	107 (%f = 68.2), aged 70−80, where Group 1 (ABAB) had 57 (M = 72.8 ± 2.6), Group 2 (BABA) 50 (M = 73.5 ± 3.2) and the active control group 18 (M = 73.5 ± 2.9), Norway	MRI (DTI)	NA (15−40)	NI	NI	NI
*Within‐subject designs*						
Fellner et al. (2016)^a^	For EEG 21 (%f = 57.14) healthy participants, aged 18−24 (M = 20.19 ± NI) and for fMRI 23 (%f = 65.21) healthy participants, aged 18−36 (M = 22.9 ± NI), Germany	EEG, fMRI	6 lists of 20 words	20	1−6	S
Kondo et al. (2005)	16 (%f = 0) healthy male adult volunteers, aged 20−26 (M = 22.3 ± NI), Japan	fMRI	30 words	10	1−3	S
Liu et al. (2022)^a^	29 (%f = 65.51) healthy participants, aged 18−24 (M = 20.3 ± NI)	fMRI	2 lists of 30 words	10	1−6	E
*Other designs*						
Raz et al. (2009) Experiment 1	1 (%f = 0) healthy, unremarkable student of mechanical engineering, aged 21 (M = NA ± NA), United States	fMRI	recalling 540 π digits	NI	NI	S
Raz et al. (2009) Experiment 2	1 (%f = 0) healthy, unremarkable student of mechanical engineering, aged 21 (M = NA ± NA), United States and 53 (%f = 45.28) healthy subjects from the authors database, aged 18−35 (M = NI ± NI), United States	fMRI	100 digits	NI	NI	S
GRADE	Low: The available evidence suggests that the evidence base might be valid. However, due to limitations, such as risk of bias, inconsistency, or imprecision, confidence in the estimate is limited. Further well‐conducted research is likely to have an important impact on our confidence in the effect estimate and may change the estimate					

*Note*: SR = serial recall, FR = free recall, NI = no information, ≈ = approximately, E = experimentator‐generated locations, S = subject‐generated locations, S + E = subject‐generated locations but controlled by experimentator, I = immediate recall, D = days, M = minutes, W = week/s, Y = year, NA = not applicable, ; = intentionally placed more than one to‐be‐remembered item per location.

^a^ Studies contributing to the effectiveness topic.

### Risk of bias in chosen studies

Tables 5, 6, 7, 8, 9, 10, 11, 12, 13, 14, 15 below summarize the results of the risk of bias assessments. All studies utilizing other group designs were deemed to have a high risk of bias.

#### Risk of bias for studies concerning effectiveness in young adults

About 89.2% of the experiments have a high risk of bias, while the remaining exhibit some concerns. Contributing factors generally include insufficient information on the randomization process, inconsistencies between the number of participants reported and those included in the statistical analyses, absence of control or comparable groups, lack of blinding for assessors and participants, and significant discrepancies or errors in study design and protocol adherence. See Tables 5, 6, 7 for the risk of bias assessment.

**TABLE 5:** The effectiveness of the method of loci in young adults: risk of bias of randomized controlled trials.

Study	D1	D2	D3	D4	D5	Overall bias
Bass and Oswald (2014)	S	H	L	S	S	H
Bower and Reitman (1972)	S	H	H	S	S	H
Carlson et al. (1976)	S	H	H	S	S	H
Cornoldi and De Beni (1991) Experiment 1	H	H	H	S	S	H
Cornoldi and De Beni (1991) Experiment 2	H	H	H	S	H	H
Crovitz (1971)	S	S	H	S	S	H
De Beni and Cornoldi (1985) Experiment 1	S	H	H	S	S	H
De Beni and Cornoldi (1985) Experiment 2	S	H	H	S	S	H
De Beni and Cornoldi (1988)	H	H	H	S	S	H
De Beni et al. (1997) Experiment 1	H	H	H	S	S	H
De Beni et al. (1997) Experiment 2	H	H	H	S	S	H
De Beni et al. (1997) Experiment 3	H	H	H	S	S	H
Groninger (1971)	S	H	H	S	S	H
Kluger et al. (2022) Experiment 1	S	H	H	S	S	H
Kroneisen and Makerud (2017) Experiment 1	S	H	L	S	S	H
Kroneisen and Makerud (2017) Experiment 2	S	H	L	S	S	H
Legge et al. (2012)	S	H	L	S	S	H
Massen and Vaterrodt‐Plünnecke (2006) Experiment 1	S	H	H	S	S	H
Massen and Vaterrodt‐Plünnecke (2006) Experiment 2	S	H	H	S	S	H
Moè and De Beni (2005)	S	H	H	S	S	H
Qureshi et al. (2014)	S	H	H	S	S	H
Roediger (1980)	S	H	H	S	S	H
Saraiva et al. (2016)	L	L	L	S	S	S
Wang and Thomas (2000)	S	H	H	S	S	H
Weinstein et al. (1981) Experiment 1	S	H	S	S	S	H

*Note*: D1 = bias due to randomization process, D2 = bias due to deviations from the intervention, D3 = bias due to missing outcome data, D4 = bias due to measurement of the outcome, D5 = bias due to selection of the reported results. L = low risk of bias, S = some risk of bias, H = high risk of bias.

**TABLE 6:** The effectiveness of the method of loci in young adults: Risk of bias for within‐subject designs.

Study	D1	D2	D3	D4	D5	D6	D7	D8	D9	Overall bias
Bouffard et al. (2018) Experiment 1	L	S	H	L	S	H	H	S	S	H
Bouffard et al. (2018) Experiment 2	L	S	H	L	S	H	H	S	S	H
Liu et al. (2022)	S	S	S	L	S	S	L	S	L	S

*Note*: D1 = cross‐over design, D2 = randomized treatment order, D3 = carry‐over effect, D4 = unbiased data, D5 = allocation concealment, D6 = blinding, D7 = incomplete data, D8 = Selective reporting, D9 = other bias. L = low risk of bias, S = some risk of bias, H = high risk of bias, + = other risk of bias.

**TABLE 7:** The effectiveness of the method of loci in young adults: Risk of bias of non‐randomized trials.

Study	D1	D2	D3	D4	D5	D6	D7	Overall bias
Dresler et al. (2017)	L	L	L	L	M	L	?	M
Mallow et al. (2015)	M	L	L	L	M	L	?	M

*Note*: D1 = bias due to confounding, D2 = bias due to selection of participants, D3 = bias in classification of intervention, D4 = bias due to deviations from the interventions, D5 = bias due to missing outcome data, D6 = bias in measurement of outcomes, D7 = bias in selection of the reported results. L = low risk of bias, M = moderate risk of bias, S = serious risk of bias, C = critical risk of bias,? = unknown risk of bias.

#### Risk of bias for studies concerning effectiveness in older adults

A total of 55.6% of experiments had high risk, while 44.4% showed serious risk. Factors contributing to this bias included insufficient control of confounding variables, such as participants’ clinical condition and education level, and non‐representative participant selection. A major concern was the high likelihood of carry‐over effects, particularly pronounced proactive and retroactive interference in older adults (Carretti et al., 2011; Hogge et al., 2008; Loewenstein et al., 2015), especially when the same locations were repeatedly used, as was often the case. See Tables 8 and 9 for the risk of bias assessment.

**TABLE 8:** The effectiveness of the method of loci in older adults: risk of bias for randomized controlled trials.

Study	D1	D2	D3	D4	D5	Overall bias
Boller et al. (2021)	L	H	H	L	S	H
Brooks et al. (1999)	S	H	H	S	S	H
Gross et al. (2014)	L	S	H	S	S	H
Hill et al. (1991)	H	H	H	S	S	H
Robertson‐Tchabo et al. (1976) Experiment 2	S	H	L	S	S	H
Yesavage and Rose (1984)	S	H	H	S	S	H

*Note*: D1 = bias due to randomization process, D2 = bias due to deviations from the intervention, D3 = bias due to missing outcome data, D4 = bias due to measurement of the outcome, D5 = bias due to selection of the reported results. L = low risk of bias, S = some risk of bias, H = high risk of bias.

**TABLE 9:** The effectiveness of the method of loci in older adults: risk of bias of non‐randomized trials.

Study	D1	D2	D3	D4	D5	D6	D7	Overall bias
Baltes and Kliegl (1992)	S	M	M	M	M	L	?	S
Kliegl et al. (1989) Experiment 1	S	L	M	M	M	L	?	S
Kliegl et al. (1989) Experiment 2	S	L	M	M	M	L	?	S
Kliegl et al. (1990)	S	M	M	M	M	L	?	S
Lindenberger et al. (1992)	S	M	M	M	L	L	?	S
Rebok and Balcerak (1989)	S	M	M	M	S	M	?	S
Verhaeghen and Marcoen (1996)	S	L	L	M	M	L	?	S
Rose and Yesavage (1983)	S	M	S	?	S	M	?	S

*Note*: D1 = bias due to confounding, D2 = bias due to selection of participants, D3 = bias in classification of intervention, D4 = bias due to deviations from the interventions, D5 = bias due to missing outcome data, D6 = bias in measurement of outcomes D7 = bias in selection of the reported results. L = low risk of bias, M = moderate risk of bias, S = serious risk of bias, C = critical risk of bias, ? = unknown risk of bias.

#### Risk of bias for studies concerning cognitive mechanisms

A total of 80% of the experiments were assessed as having a high risk of bias, 6.67% as having a serious risk of bias, and the remaining 13.3% were judged to have some concerns. Contributing factors included insufficient reporting of the randomization process, missing data, inconsistencies between participant characteristics and statistical analyses, lack of assessor and participant blinding, and critical design flaws, such as the absence of a valid control group. See Tables 10, 11, 12 for the risk of bias assessment.

**TABLE 10:** The cognitive mechanisms underlying the effectiveness of the method of loci: risk of bias of randomized control trials.

Study	D1	D2	D3	D4	D5	Overall bias
Bellezza and Reddy (1978)	S	H	H	S	S	H
Blunt and VanArsdall (2021) Experiment 1	S	H	H	S	S	H
Blunt and VanArsdall (2021) Experiment 2	S	S	H	S	S	H
Kemp and Van der Krogt (1985) Experiment 1	S	H	L	S	S	H
Massen et al. (2009) Experiment 1	S	S	L	S	S	S
Massen et al. (2009) Experiment 2	S	S	H	S	S	H
Reggente et al. (2020)	S	S	L	S	S	S

*Note*: D1 = bias due to randomisation process, D2 = bias due to deviations from the intervention, D3 = bias due to missing outcome data, D4 = bias due to measurement of the outcome, D5 = bias due to selection of the reported results. L = low risk of bias, S = some risk of bias, H = high risk of bias.

**TABLE 11:** The cognitive mechanisms underlying the effectiveness of the method of loci: risk of bias of non‐randomized control trials.

Study	D1	D2	D3	D4	D5	D6	D7	Overall bias
Kemp and Van der Krogt (1985) Experiment 2	S	S	M	M	L	L	?	S

*Note*: D1 = bias due to confounding, D2 = bias due to selection of participants, D3 = bias in classification of intervention, D4 = bias due to deviations from the interventions, D5 = bias due to missing outcome data, D6 = bias in measurement of outcomes, D7 = bias in selection of the reported results. L = low risk of bias, M = moderate risk of bias, S = serious risk of bias, C = critical risk of bias, ? = unknown risk of bias.

**TABLE 12:** The cognitive mechanisms underlying the effectiveness of the method of loci: risk of bias for within‐subject designs.

Study	D1	D2	D3	D4	D5	D6	D7	D8	D9	Overall bias
Canellopoulou and Richardson (1998)	H	H	S	L	H	H	L	S	S	H
Caplan et al. (2019)	H	S	S	L	S	H	L	S	H	H
Lea (1975) Experiment 1	S	S	H	L	H	H	L	S	S	H
Lea (1975) Experiment 2	S	S	H	L	H	H	L	S	S	H
Lea (1975) Experiment 3	S	S	H	L	H	H	L	S	S	H

*Note*: D1 = cross‐over design, D2 = randomized treatment order, D3 = carry‐over effect, D4 = unbiased data, D5 = allocation concealment, D6 = blinding, D7 = incomplete data, D8 = selective reporting, D9 = other bias. L = low risk of bias, S = some risk of bias, H = high risk of bias, + = other risk of bias.

#### Risk of bias for studies concerning neuropsychology

A total of 26.67% of the experiments had a high or serious risk of bias, while 73.33% had some or moderate risk. Key factors contributing to the risk of bias include confounding variables, missing data, very small sample sizes, and results variability. However, neuropsychological studies provide significantly higher quality evidence compared with those previously discussed due to more robust methodologies and neuroimaging methods. See Tables 13, 14, 15 for the risk of bias assessment.

**TABLE 13:** The method of loci in the context of neuropsychology: risk of bias of randomized trials.

Study	D1	D2	D3	D4	D5	Overall bias
de Lange et al. (2018)	S	S	L	S	S	S

*Note*: D1 = bias due to randomisation process, D2 = bias due to deviations from the intervention, D3 = bias due to missing outcome data, D4 = bias due to measurement of the outcome, D5 = bias due to selection of the reported results. L = low risk of bias, S = some risk of bias, H = high risk of bias.

**TABLE 14:** The method of loci in the context of neuropsychology: risk of bias of non‐randomized trials.

Study	D1	D2	D3	D4	D5	D6	D7	Overall bias
Belleville et al. (2023)	M	L	L	L	M	L	?	M
Dresler et al. (2017)	L	L	L	L	M	L	?	M
Engvig et al. (2010)	L	L	L	L	M	L	?	M
Maguire et al. (2003)	S	L	L	S	L	L	?	S
Mallow et al. (2015)	M	L	L	L	M	L	?	M
Müller et al. (2018)	M	L	L	L	L	L	?	M
Nyberg et al. (2003)	M	L	L	L	?	L	?	M
Wagner et al. (2021) Experiment 2	M	L	L	L	M	L	?	M
Wagner et al. (2021) Experiment 1	M	L	L	L	M	L	?	M

*Note*: D1 = bias due to confounding, D2 = bias due to selection of participants, D3 = bias in classification of intervention, D4 = bias due to deviations from the interventions, D5 = bias due to missing outcome data, D6 = bias in measurement of outcomes, D7 = bias in selection of the reported results. L = low risk of bias, M = moderate risk of bias, S = serious risk of bias, C = critical risk of bias, ? = unknown risk of bias.

**TABLE 15:** The method of loci in the context of neuropsychology: risk of bias for within‐subject designs.

Study	D1	D2	D3	D4	D5	D6	D7	D8	D9	Overall bias
Fellner et al. (2016)	H	S	H	S	S	S	L	S	L	H
Kondo et al. (2005)	S	S	L	L	S	S	L	S	L	S
Liu et al. (2022)	S	S	S	L	S	S	L	S	L	S

*Note*: D1 = cross‐over design, D2 = randomized treatment order, D3 = carry‐over effect, D4 = unbiased data, D5 = allocation concealment, D6 = blinding, D7 = incomplete data, D8 = selective reporting, D9 = other bias. L = low risk of bias, S = some risk of bias, H = high risk of bias.

## SYNTHESES

In the following syntheses, we first present both the quantitative (RoBMA_PSMA_) syntheses of the effectiveness of MoL in young, older, and all adults in immediate serial recall, where usable effect sizes were available, and the qualitative (narrative and GRADE) syntheses of the general MoL's effectiveness in the same populations. Second, analyses of cognitive mechanisms and neuropsychological aspects are presented only in qualitative form (narrative and GRADE) due to the nature of the topic and the absence of quantifiable data.

Effect sizes for the quantitative analyses were calculated using various methods, depending on the inferential statistics reported in the individual studies. However, we were not able to calculate effect sizes for all studies. Moreover, in studies where calculation was possible, not all effect sizes could be calculated using the same primary formula (i.e. based on *M*, *SD* or *SE*). Effect sizes calculated using alternative formulas (e.g. based on *t* or *p* values and *N*) resulted in variable and often imprecise estimates. Therefore, to ensure the highest possible accuracy of the RoBMA_PSMA_ results, we used only one formula (based only on *M*, *SD* or *SE* if reported). As a result, only data related to immediate serial recall could be included. The additional meta‐analyses with mixed formulas and further insights are in Appendix S1.

### The general effectiveness of the method of loci in young adults

The research of the effectiveness of the MoL in young adults has been investigated from three main perspectives. First, by distinguishing between serial recall, where participants reproduce items in the exact order presented, and free recall, which allows retrieval in any order. Second, by examining retention intervals, ranging from immediate to long‐delayed recall. Third, by comparing the method to other learning strategies, including rehearsal (repeating of to‐be‐remembered items), free learning strategy (spontaneous, unguided learning without explicit instruction or mnemonic support), and alternative mnemonics (structured techniques other than the method of loci, such as the Pegword method, Keyword method, or Story method).

Effect sizes were possible to calculate for 18 out of 37 experiments, but only 13 effect sizes assessing serial immediate recall compared with rehearsal were included in the meta‐analysis due to differing calculation formulas. Table 16 summarizes the effect sizes and the formulas used.

**TABLE 16:** Studies with calculable effect sizes when comparing the method of loci with rehearsal in serial immediate recall.

Citation	Serial recall (*d*, 95% CI)
Cornoldi and De Beni (1991) Experiment 1 ^a^	Oral presentation: *d =* 0.98 ^b^, [0.10, 1.84] Written presentation: *d =* 0.60, [−0.32, 1.52] Weighted Mean Effect Size: *d* = 0.80, [0.17, 1.43]
De Beni et al. (1997) Experiment 1^c^	MoL vs. Rehearsal: *d =* 1.44, [1.05, 1.84]
De Beni et al. (1997) Experiment 2 ^a^	Written presentation: *d =* −0.45, [−1.39, 0.49] Oral presentation: *d =* 0.80 ^b^, [−0.17, 1.76] Weighted Mean Effect Size: *d* = 0.16, [−0.51, 0.83]
De Beni et al. (1997) Experiment 3 ^a^	Written presentation: *d =* −1.46, [−2.57, −0.37] Oral presentation: *d =* 4.25 ^b^, [2.48, 6.03] Weighted Mean Effect Size: *d* = 0.12, [−0.81, 1.06]
De Beni and Cornoldi (1985) Experiment 2^d^	*d* = 1.08, [0.47, 1.68]
De Beni and Cornoldi (1988) ^a^	Concrete words: List 1: *d =* 0.28, [−0.11, 0.67], List 2: *d =* 0.67, [0.27, 1.06], List 3: *d =* 0.41, [0.02, 0.80] Abstract words: List 1: *d* = 0.54, [0.15, 0.93], List 2: *d =* 1.20, [0.78, 1.62], List 3: *d =* 0.78, [0.38, 1.17] Weighted Mean Effect Size: *d* = 0.63, [0.47, 0.79]
Kroneisen and Makerud (2017) Experiment 1^NCE^	(MoL vs. Survival vs. Imagery: *η*_p_ ^e^ = 0.37)
Kroneisen and Makerud (2017) Experiment 2^NCE^	(MoL vs. Survival vs. Control: *η*_p_ ^e^ = 0.28)
Legge et al. (2012) ^f^	All participants (group: *η*_p_ ^e^ = 0.002, *p* > .1) Compliant only (group: *η*_p_ ^e^ = 0.14): cMOL vs. CON: *d* = 0.47, [0.06, 0.88]; vMOL vs. CON: *d* = 0.33 [−0.07, 0.73]; cMOL vs. vMOL: *d* = 0.13 [−0.27, 0.53] Weighted Mean Effect Size: *d* = 0.40, [0.11, 0.68]
Liu et al. (2022) ^f^	MoL vs. baseline: *d* = 1.56, [0.98, 2.14]; MoL vs. 1st practice: *d* = 1.14, [0.64, 1.64]; MoL vs. last practice: *d* = 0.75, [0.31, 1.19] Weighted Mean Effect Size: *d* = 1.08, [0.79, 1.36]
Mallow et al. (2015) ^f^	MoL vs. Control in digit recall: *d* > 4, [2.72, 5.28]
Massen and Vaterrodt‐Plünnecke (2006) Experiment 1 ^a^	Loci Similar vs. Rehearsal Similar: *d* = 0.43, [−0.18, 1.05] for List 1; *d* = 0.23, [−0.38, 0.84] for List 2; *d* = 0.32, [−0.29, 0.93] for List 3 Loci Dissimilar vs. Rehearsal Dissimilar: *d* = 0.04, [−0.57, 0.65] for List 1; *d* = 0.41, [−0.20, 1.03] for List 2; *d* = 1.07, [0.42, 1.72] for List 3 Weighted Mean Effect Size: *d* = 0.40, [0.15, 0.66] Loci Similar: *d* = −0.41, 95% CI [−0.88, 0.05] indicating a moderate decline in performance Loci Dissimilar: *d* = 0.91, 95% CI [0.38, 1.44] indicating a significant improvement in performance
Massen and Vaterrodt‐Plünnecke (2006) Experiment 2 ^a^	Loci Similar vs. Rehearsal Similar: *d* = 0.77, [0.26, 1.27] for List 1; *d* = 0.46, [0.00, 0.93] for List 2; *d* = 0.29, [−0.16, 0.74] for List 3 Loci Dissimilar vs. Rehearsal Dissimilar: *d* = 0.21, [−0.24, 0.66] for List 1; *d* = 0.53, [0.06, 1.01] for List 2; *d* = 0.35, [−0.10, 0.81] for List 3 Weighted Mean Effect Size: *d* = 0.42, [0.23, 0.61] Loci Similar: *d* = −0.11, 95% CI [−0.56, 0.33] indicating a slight decline in performance Loci Dissimilar: *d* = 0.24, 95% CI [−0.21, 0.69] indicating a slight improvement in performance
McCabe (2015) ^a^	*d* = 0.81, [0.54, 1.08]
Moè and De Beni (2005) ^a?^	*Oral presentation*: Descriptive passage: *d* = 0.82, [−0.09, 1.73] for Subject‐generated vs. rehearsal; *d* = 0.36, [−0.53, 1.24] for Experimentator‐genereted vs. rehearsal; *d* = 0.55, [−0.34, 1.44] for Subject vs. Experimentator‐generated. Narrative passage: *d* = 1.42, [0.44, 2.40] for Subject‐generated vs. rehearsal; *d* = 1.36, [0.39, 2.34] for Experimentator‐genereted vs. rehearsal; *d* = 0.16, [−0.72, 1.04] for Subject vs. Experimentator‐generated. Expository passage: *d* = 2.04, [0.96, 3.12] for Subject‐generated vs. rehearsal; *d* = 1.32, [0.35, 2.28] for Experimentator‐genereted vs. rehearsal; *d* = 0.64, [−0.26, 1.54] for Subject vs. Experimentator‐generated Weighted Mean Effect Size: *d* = 0.93, [0.70, 1.16] *Written presentation*: Descriptive passage: *d* = −1.13, [−2.07, −0.18] for Subject‐generated vs. rehearsal; *d* = −0.65, [−1.55, 0.24] for Experimentator‐generated vs. rehearsal; *d* = −0.49, [−1.38, 0.40] for Subject vs. Experimentator‐generated. Narrative passage: *d* = −0.50, [−1.39, 0.39] for Subject‐generated vs. rehearsal; *d* = 0.12, [−0.76, 1.00] for Experimentator‐generated vs. rehearsal; *d* = −0.70, [−1.61, 0.20] for Subject vs. Experimentator‐generated. Expository passage: *d* = −1.94, [−3.00, −0.87] for Subject‐generated vs. rehearsal; *d* = −1.40, [−2.37, −0.42] for Experimentator‐generated vs. rehearsal; *d* = −0.45, [−1.34, 0.44] for Subject vs. Experimentator‐generated. Weighted Mean Effect Size: *d* = −0.76, [−0.99, −0.54] Written and Oral presentation Weighted Mean Effect Size: *d* = 0.13, [−0.14, 0.40] *Comparsion between passages*: Descriptive passage: *d* = 1.13, [0.19, 2.10] for Subject‐generated Oral vs. Written; *d* = 0.23, [−0.65, 1.11] for Experimentator‐generated Oral vs. Written. Narrative passage: *d* = 0.84, [−0.07, 1.76] for Subject‐generated Oral vs. Written; *d* = 0.14, [−0.74, 1.02] for Experimentator‐generated Oral vs. Written. Expository passage: *d* = 2.33, [1.20, 3.47] for Subject‐generated Oral vs. Written; *d* = 1.01, [0.08, 1.94] for Experimentator‐generated Oral vs. Written. *All passages together comparsion of written* vs *oral presentation*: Subject‐generated: *d* = −1.67, [−2.26, −1.08]; Experimenter‐generated: *d* = −0.66, [−1.18, −0.14] *All passages together comparsion oral*: Subject‐generated vs. rehearsal: *d* = 2.06, [1.44, 2.67]; Experimenter‐generated vs. rehearsal: *d* = 1.56, [0.99, 2.14]; Subject vs. Experimentator‐generated: *d* = 0.43, [−0.08, 0.94] *All passages together comparsion written*: Subject‐generated vs. rehearsal: *d* = −1.23, [−1.79, −0.68]; Experimenter‐generated vs. rehearsal: *d* = −0.68, [−1.20, −0.16]; Subject vs. Experimentator‐generated: *d* = −0.74, [−1.26, −0.22]
Roediger (1980)^g^	MoL vs. Rehearsal: *d* = 2.18, [1.55, 2.82]
Saraiva et al. ( 2016 ) ^a^	MoL vs. Free strategy (*η* ^e^ = 0.73): *d* = 9.39, [6.82, 11.96]
Weinstein et al. ( 1981 ) Experiment 1 ^a^	Elaborated instructions MoL vs. rehearsal: *d* = 0.86, [0.21, 1.50] Training MoL vs. rehearsal: *d* = 2.10, [1.33, 2.88] Elaborated training MoL vs. rehearsal: *d* = 1.90, [1.16, 2.65] Standard MoL vs. rehearsal: *d* = 0.16, [−0.47, 0.78] Weighted Mean Effect Size: *d* = 1.11, [0.77, 1.46]

*Note*: NCE = no calculable effect sizes for a specific method. Underscored studies = studies used for the main meta‐analysis. Underscored effect sizes = effect sizes used for calculating weighted means.

^a^ Means, standard deviations, and sample sizes.

^b^ Only these effect sizes were used for the primary meta‐analysis because written presentations were intentionally designed to create interference between the use of the method of loci and the act of reading the presented text, which, in my opinion, makes the resulting effects unreliable and unusable for meaningful analysis.

^c^ One‐way *F*‐test with two groups and equal sample sizes.

^d^ Student's *t*‐test and total sample size.

^e^ *p*‐value from a Student's *t*‐test with unequal sample sizes.

^f^ Effect sizes reported, 95% CI calculated using formula CI = *d* ± Z*SE_d_.

^g^ One‐way ANOVA with three or more groups.

#### The effectiveness of the method of loci compared with rehearsal in immediate serial recall in young adults

A RoBMA_PSMA_ based on 936 participants (weighted mean age = 23.89, aged 17–44) was used to evaluate the effectiveness of the MoL compared with rehearsal in immediate serial recall in young adults. Moderate evidence was found for a small effect (*d* = 0.42, 95% CI [0.00, 0.80], [P(M|data) = 0.86, BF = 6.24]), with very strong evidence for moderate heterogeneity (*τ* = 0.46, 95% CI [0.18, 0.90], [P(M|data) = 1, BF = 12196.17]) and publication bias ([P(M|data) = 1, BF = 6.41 × 10^6^]). PET–PEESE analyses indicated the presence of small‐study effects, with the PET model showing no evidence of an effect (*d* = 0.00, 95% CI [0.00, 0.00]), and PEESE showing an implausibly large estimate (*d* = 9.11, 95% CI [7.13, 11.35]).

**FIGURE 2:**
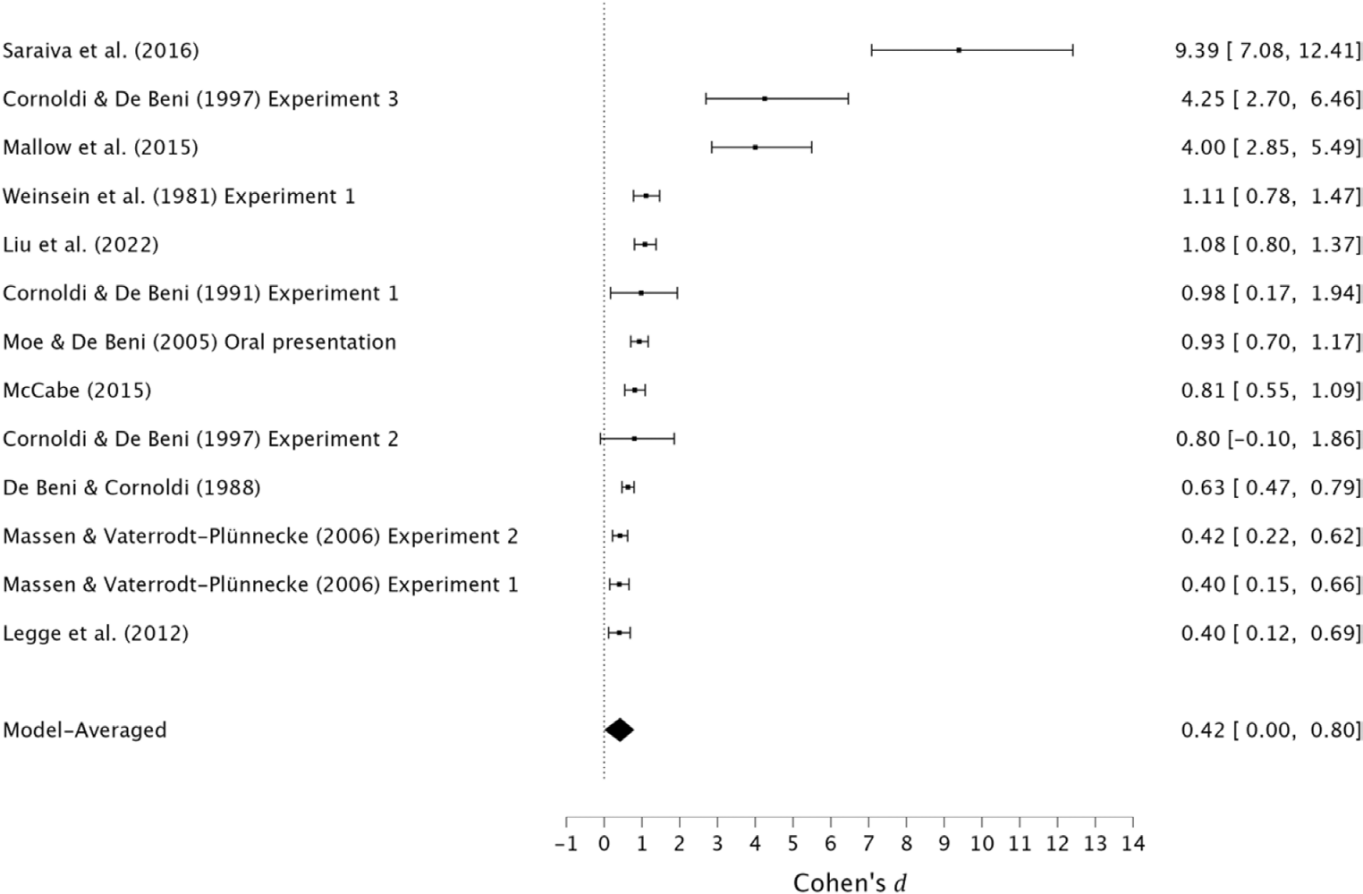
Meta‐analysis forest plot of the effectiveness of the method of loci compared with rehearsal in immediate serial recall among young adults.

In conclusion, moderate evidence supports a small effect of the MoL in enhancing immediate serial recall in young adults. However, very strong evidence for moderate heterogeneity, very strong evidence for publication bias, and the presence of small‐study effects suggests potential inflation of effect sizes, advising caution in interpreting the findings. See Figure 2 for forest plots.

#### The narrative synthesis of the general effectiveness of the method of loci in young adults

The MoL consistently demonstrates effectiveness across various memory retrieval tasks, including free recall, serial recall, recognition, and knowledge‐based assessments. Despite variations in materials and experimental procedures, many studies converge on MoL's capacity to enhance both the quantity and quality of remembered information.

##### The effectiveness of the method of loci in different types of memory recall

###### Free recall

A total of 15 experiments evaluated MoL's impact on free recall, generally demonstrating substantial improvements, reduction of interference, and retention over time. Bass and Oswald (2014) found that MoL reduced proactive interference by ~25%, markedly enhancing recall. Carlson et al. (1976) demonstrated that spontaneous MoL use by students achieved similar recall success as explicit MoL instruction, emphasizing its intuitive applicability. Wang and Thomas (2000) indicated that MoL outperformed maintenance rehearsal, especially after retention intervals, while Roediger (1980) found MoL equally effective as the Pegword method but superior to simpler link strategies. Kroneisen and Makerud (2017) reported that MoL provided advantages over survival processing, particularly with abstract material. McCabe (2015) further established MoL's classroom utility, doubling students’ performance and promoting broader use. Bouffard et al. (2018), Fellner et al. (2016), and Kliegl et al. (1987) consistently showed enhanced recall with MoL compared with temporal or non‐spatial methods. Legge et al. (2012), Moll and Sykes (2023), and Ross and Lawrence (1968) further confirmed MoL's efficacy in enhancing free recall across virtual reality (VR) and traditional settings, maintaining high recall and minimal interference effects.

###### Serial recall

A total of 28 experiments have investigated the MoL for its exceptional ability to maintain strict item order during serial recall. Its primary advantage lies in preserving precise item sequences even in environments prone to interference. Early investigations by Ross and Lawrence (1968) and Bower and Reitman (1972) observed minimal interference and high accuracy over extended intervals. Dresler et al. (2017) found that mnemonic training using the MoL led to significant, enduring improvements in memory performance – even among individuals unfamiliar with such strategies. In a similar vein, Liu et al. (2022) provided compelling neuroimaging evidence demonstrating that the MoL markedly enhances temporal order memory.

De Beni and Cornoldi (1985, 1988) consistently reported the MoL's superiority in both immediate and delayed recall, emphasizing its resilience against proactive interference, while also advising caution regarding retroactive interference when loci are reused. Massen and Vaterrodt‐Plünnecke (2006) further reinforced the method's effectiveness in overcoming interference, and Saraiva et al. (2016) showed that the MoL effectively eliminates collaborative inhibition by offering a consistent retrieval framework.

Additional studies have underscored the MoL's versatility across various content types. Kluger et al. (2022) demonstrated that the MoL – and similar strategies such as the Body Scaffold—yield superior serial recall compared with less structured methods. Kliegl et al. (1987) illustrated that integrating the MoL with domain‐specific knowledge can boost digit recall, although factors like encoding speed and prior expertise are crucial. Moreover, Moè and De Beni (2005) showed that applying the MoL to expository passages in oral presentations is particularly beneficial when participants generate their own pathways. Finally, Mallow et al. (2015) provided clear evidence that skilled memory users employing the MoL not only recalled significantly more digits but also did so more rapidly than their less experienced counterparts.

#### Recognition and Multiple‐Choice Questionnaires

Recognition tasks appeared in two experiments, with mixed outcomes. Groninger (1971) found high recognition performance across groups but superior recall among MoL users due to stronger retrieval cues. De Beni and Cornoldi (1985) noted minimal advantages for MoL in recognition tasks, suggesting that MoL primarily enhances ordered recall rather than recognition memory.

Qureshi et al. (2014) uniquely explored MoL in MCQ tasks, demonstrating that medical students using MoL scored significantly higher than controls. Despite highlighting practical challenges, such as the time‐intensive creation of memory palaces, students reported greater confidence and favourable perceptions, underscoring MoL's potential in high‐stakes educational contexts.

##### Proactive and retroactive interference

The MoL consistently demonstrates resilience against interference, particularly proactive interference. Bass and Oswald (2014), Massen and Vaterrodt‐Plünnecke (2006), and Kluger et al. (2022) reported reduced proactive interference effects, emphasizing MoL's efficacy in minimizing retrieval competition between encoded items. However, De Beni and Cornoldi (1988) observed retroactive interference when loci were repeatedly reused across different materials, suggesting careful management of loci reuse. Groninger (1971) and Ross and Lawrence (1968) also cautioned about increased intrusion errors at extended intervals.

##### Comparison to other mnemonics

Direct comparisons underscore MoL's robust effectiveness relative to alternative mnemonic techniques. While Bower and Reitman (1972) and Roediger (1980) observed comparable outcomes between MoL and Pegword systems, Fellner et al. (2016) reported advantages for MoL over the nonspatial Pegword system. MoL consistently outperformed simpler link methods (Massen & Vaterrodt‐Plünnecke, 2006; Roediger, 1980) and showed advantages over survival processing for abstract words (Kroneisen & Makerud, 2017). Although temporal mnemonic or body scaffolds improved with practice, MoL typically provided immediate and sustained recall benefits (Bouffard et al., 2018; Kluger et al., 2022).

##### Practical applications and modality effects

MoL demonstrates versatility across educational and professional settings. Classroom applications by McCabe (2015) and medical education reported by Qureshi et al. (2014) illustrate its practical benefits. Technological advancements further support MoL's utility, with VR implementations by Fassbender and Heiden (2006), Legge et al. (2012), and Moll and Sykes (2023) showing equivalent or superior effectiveness compared with classical MoL. Conversely, Cornoldi and De Beni (1991), De Beni et al. (1997), and Moè and De Beni (2005) suggest that written or computer‐assisted presentations can sometimes reduce MoL's effectiveness, emphasizing the need for oral modality considerations.

##### Limitations and constraints

Although the outcomes are promising, several limitations deserve careful consideration. Practical constraints include a recommended two‐item per locus limit (Crovitz, 1971), which restricts the overall ‘storage capacity’ of a memory palace. In addition, creating memory palaces is time‐intensive (Qureshi et al., 2014), and achieving rapid encoding requires considerable practice (Kliegl et al., 1987). Moreover, intrusion errors have been observed at longer delays (Groninger, 1971), and reusing loci can lead to retroactive interference (De Beni & Cornoldi, 1988).

##### Quality of evidence

Despite its reported benefits, our evaluation revealed several limitations. Specifically, 89.2% of the experiments were at high risk of bias, and the results were imprecise. We found very strong evidence of moderate heterogeneity and publication bias, along with moderately strong evidence indicating the presence of small‐study effect. Consequently, the overall quality of evidence (GRADE) for the general effectiveness of the method of loci in young adults is very low, meaning that the true effect may be substantially different from our estimate.

##### The combined conclusion of qualitative and narrative syntheses

The MoL consistently demonstrates improvements in both free and serial recall performance, showing clear advantages over rehearsal and other mnemonics, with either superior or equivalent effectiveness – particularly with moderate evidence for a small effect in immediate serial recall among young adults (without the leveraged data from young adult subgroups in older adult‐focused research; see below a meta‐analysis of leveraged data from young adult subgroups in older adult–focused research compared with rehearsal in immediate serial recall).

Despite these benefits, substantial methodological concerns remain, including a high risk of bias in most of the included studies, very strong evidence of moderate heterogeneity, very strong evidence of publication bias, the presence of small‐study effects, and a very low GRADE rating. These issues necessitate caution when interpreting the magnitude and generalizability of the findings.

Nevertheless, the strengths of the MoL remain compelling, reinforcing its significant potential for enhancing memory performance across various contexts. Further rigorous empirical research is needed to clarify the true extent of its effectiveness.

### The general effectiveness of the method of loci in older adults

The effectiveness of the MoL in old adults has been studied from two perspectives: (1) comparing pre‐test and post‐test results and (2) comparing elderly participants with younger participants. Comparisons with other memory techniques, retention intervals, or recall types beyond immediate serial recall are rare, with only Hill et al. (1991) providing such a comparison. Only 9 experiments out of 18 were included in the effect size calculations due to missing inference data. Except for Gross et al. (2014), all effect sizes and confidence intervals were calculated additionally. Table 17 summarizes the effect sizes and formulas used.

**TABLE 17:** Studies with calculable effect sizes when comparing the method of loci with rehearsal or free learning strategies in immediate recall pre‐post intervention and comparison with young adults.

Citation	Immediate recall (*d*, 95% CI)
Brooks et al. (1999) ^a^	*Memory training, regular, pre‐post*: Standard: *d =* 0.59, [0.21, 0.96], Comprehensive: *d =* 0.95, [0.56, 1.34] and *Memory training, extended, pre‐post*: Standard: *d =* 0.85, [0.46, 1.23], Comprehensive: *d =* 0.87, [0.48, 1.26]; Weighted Mean Effect Size: *d* = 0.81, [0.62,1.00]
Gross et al. (2014) ^b^	*Memory‐trained skippers* vs. *Control group*: *d* = 2.1, [1.91, 2.29] and *Memory‐trained skippers* vs. *never‐kippers*: *d* = 2.4, [2.19, 2.61] Weighted Mean Effect Size: *d* = 2.25, [2.05, 2.45]
Hill et al. (1991) ^a^	*Immediate recall*: MoL vs. Rehearsal: *d =* 0.75, [0.17, 1.33]; MoL vs. Story: *d =* 0.43, [−0.15, 1.00]; MoL pre‐post: *d =* 1.13 [0.56, 1.70] Weighted Mean Effect Size: *d* = 0.94, [0.54, 1.35] *1‐hour delayed recall*: MoL vs. Rehearsal: *d =* 0.74, [0.16, 1.32]; MoL vs. Story: *d =* 0.38, [−0.19, 0.96]; MoL pre‐post: *d =* 0.94, [0.38, 1.50] Weighted Mean Effect Size: *d* = 0.84, [0.44, 1.25] *3‐days delayed recall*: MoL vs. Rehearsal: *d =* 0.81, [0.23, 1.39]; MoL vs. Story: *d =* 0.42, [−0.16, 1.00]; MoL pre‐post: *d =* 0.19, [−0.35, 0.72] Weighted Mean Effect Size: *d* = 0.48, [0.08, 0.87]
Kliegl et al. (1989) Experiment 1 ^a^	*Post Y* vs *O*: Self‐paced: *d =* 1.33, [0.19, 2.46]; 10s: *d =* 1.60, [0.44, 2.77]; 4 s: *d =* 3.1, [1.7, 4.48] Weighted Mean Effect Size: d = 1.88, [1.18, 2.58] *Pre‐post O*: 10s vs. 10s: *d =* 2.79, [1.92, 3.66]; 4 s vs. 4 s: *d =* 1.04, [0.37, 1.70]; 10s vs. self‐paced: *d* = 6.58, [5.01, 8.15] Weighted Mean Effect Size: d = 2.18, [1.68, 2.68] *Pre‐post Y*: 10s vs. 10s: *d* = 11.93, [5.92, 17.94]; 4 s vs. 4 s: *d* = 3.25, [1.14, 5.35]; 10s vs. self‐paced: *d* = 14.83, [7.43, 22.23] Weighted Mean Effect Size: *d* = 4.90, [2.99, 6.81] Young + Old pre‐post Weighted Mean Effect Size: *d* = 2.35, [1.87, 2.84]
Kliegl et al. (1989) Experiment 2 ^a^	*Post Y* vs *O*: 20s: *d* = 1.71, [1.04, 2.38]; 15 s: *d* = 1.87, [1.20, 2.54]; 10s: *d* = 1.93, [1.26, 2.60]; 5 s: *d* = 2.51, [1.84, 3.18]; 3 s: *d* = 2.02, [1.35, 2.69]; 1 s: *d* = 1.04, [0.37, 1.70]; Overall: *d* = 2.79, [2.12, 3.46] Weighted Mean Effect Size: *d* = 1.98, [1.88, 2.08] *Pre‐post Y*: 20s: *d* = 4.60, [4.10, 5.10]; 15 s: *d* = 4.05, [3.55, 4.54]; 10s: *d* = 2.37, [1.87, 2.87]; 5 s: *d* = 3.19, [2.69, 3.69]; 3 s: *d* = 1.84, [1.34, 2.34]; 1 s: *d* = 1.18, [0.68, 1.67]; Overall: *d* = 4.40, [3.91, 4.90] Weighted Mean Effect Size: *d* = 3.09, [2.98, 3.20] *Pre‐post O*: 20s: *d* = 2.05, [1.56, 2.53]; 15 s: *d* = 1.91, [1.43, 2.39]; 10s: *d* = 2.00, [1.52, 2.49]; 5 s: *d* = 1.73, [1.24, 2.21]; 3 s: *d* = 0.91, [0.43, 1.39]; 1 s: *d* = 0.62, [0.14, 1.11]; Overall: *d* = 2.38, [1.90, 2.86] Weighted Mean Effect Size: *d* = 1.66, [1.56, 1.76] Young + Old pre‐post Weighted Mean Effect Size: *d* = 2.31, [2.23, 2.38]
Kliegl et al. (1990) ^a^	*Pre‐post Y*: 20 s: *d* = 4.60, [3.35, 5.85]; 15 s: *d* = 4.05, [2.91, 5.19]; 10 s: *d* = 2.37, [1.52, 3.22]; 5 s: *d* = 3.19, [2.20, 4.18]; 3 s: *d* = 1.84, [1.06, 2.62]; 1 s: *d* = 1.18, [0.47, 1.88]; Overall: *d* = 4.40, [3.20, 5.61] Weighted Mean Effect Size: *d* = 2.41, [2.04, 2.77] *Pre‐post O*: 20 s: *d* = 2.05, [1.26, 2.83]; 15 s: *d* = 1.91, [1.14, 2.68]; 10 s: *d* = 2.01, [1.23, 2.78]; 5 s: *d* = 1.70, [0.96, 2.44]; 3 s: *d* = 0.91, [0.24, 1.58]; 1 s: *d* = 0.62, [−0.03, 1.28]; Overall: *d* = 2.38, [1.55, 3.21] Weighted Mean Effect Size: *d* = 1.45, [1.15, 1.75] *Post Y* vs. *O*: 20 s: *d* = 1.71, [0.96, 2.46]; 15 s: *d* = 1.87, [1.10, 2.64]; 10s: *d* = 1.93, [1.15, 2.71]; 5 s: *d* = 2.51, [1.65, 3.37]; 3 s: *d* = 2.02, [1.23, 2.81]; 1 s: *d* = 1.04, [0.35, 1.72]; Overall: *d* = 2.79, [1.88, 3.70] Weighted Mean Effect Size: *d* = 1.79, [1.47, 2.10] Young + Old pre‐post Weighted Mean Effect Size: *d* = 1.84, [1.61, 2.07]
Lindenberger et al. (1992) ^a^	*Normal Y* vs. *Normal O*: 7.5 s: *d =* 4.33, [2.26, 6.39]; 4.5 s: *d =* 4.06, [2.08, 6.03]; 1.5 s: *d =* 3.97, [2.02, 5.92] *Normal Y* vs. *Designers O*: 7.5 s: *d =* 2.37, [0.90, 3.85]; 4.5 s: *d =* 2.80, [1.21, 4.40]; 1.5 s: *d =* 3.26, [1.54, 4.99] *Designers O* vs. *Normal O*: 7.5 s: *d =* 2.17, [0.74, 3.60]; 4.5 s: *d =* 1.75, [0.42, 3.08]; 1.5 s: *d =* 1.11, [−0.11, 2.33] *Designers Y* vs. *Designers O*: 7.5 s: *d =* 3.44, [1.66, 5.22]; 4.5 s: *d =* 4.68, [2.49, 6.86]; 1.5 s: *d =* 3.81, [1.91, 5.71] *Designers Y* vs. *Normal O*: 7.5 s: *d =* 2.17, [0.74, 3.59]; 4.5 s: *d =* 2.30, [0.84, 3.76]; 1.5 s: *d =* 1.83, [0.49, 3.18] Weighted Mean Effect Size for all Young vs. Old comparsions: *d* = 2.97, [2.49,3.46]
Rebok and Balcerak (1989) ^a^	*Pre‐post O*: No Tr/No Fd: *d* = 0.15, [−0.26, 0.56]; No Tr/Fd: *d* = 0.44, [0.03, 0.86]; Tr/No Fd: *d* = 1.16, [0.71, 1.61]; Tr/Fd: *d* = 1.12, [0.67, 1.56] Weighted Mean Effect Size: *d* = 0.72, [0.51, 0.92] *Seassion 1 Y* vs. *O*: No Tr/No Fd: *d* = 1.60, [1.13, 2.07]; No Tr/Fd: *d* = 2.60, [2.05, 3.15]; Tr/No Fd: *d* = 2.16, [1.65, 2.67]; Tr/Fd: *d* = 1.58, [1.12, 2.05] Weighted Mean Effect Size: *d* = 1.99, [1.78, 2.19] *Seassion 3 Y* vs *O*: No Tr/No Fd: *d* = 2.33, [1.81, 2.86]; No Tr/Fd: *d* = 2.50, [1.96, 3.04]; Tr/No Fd: *d* = 3.09, [2.49, 3.69]; Tr/Fd: *d* = 1.72, [1.25, 2.20] Weighted Mean Effect Size: *d* = 2.41, [2.20, 2.62] *Seassion 1 and 3 Y* vs. *O*: Weighted Mean Effect Size: *d* = 2.12, [1.94, 2.30] *Pre‐post Y*: No Tr/No Fd: *d* = 0.37, [−0.03, 0.78]; No Tr/Fd: *d* = 0.52, [0.11, 0.92]; Tr/No Fd: *d* = 1.99, [1.50, 2.48]; Tr/Fd: *d* = 1.67, [1.21, 2.13] Weighted Mean Effect Size: *d* = 1.14, [0.94, 1.34] Young + Old pre‐post Weighted Mean Effect Size: *d* = 0.94, [0.79, 1.08]
Verhaeghen and Marcoen (1996) ^a^	*Pre‐post Y*: W6m: *d =* 1.62, [1.22, 2.02], CS6m: *d =* 1.66, [1.23, 2.08]; W4m: *d =* 1.15, [0.78, 1.54], CS4m: *d =* 1.27, [0.87, 1.68]; W2m: *d* = 0.98 [0.61, 1.35], CS2m: *d =* 1.26, [0.87, 1.67] Weighted Mean Effect Size: *d* = 1.23, [1.01, 1.45] *Pre‐post O*: W6m: *d =* 0.90, [0.57, 1.23], CS6m: *d =* 1.77, [1.33, 2.20]; W4m: *d* = 0.49, [0.17, 0.81], CS4m: *d =* 1.18, [0.78, 1.58]; W2m: *d =* 0.45, [0.13, 0.77], CS2m: *d =* 0.51, [0.12, 0.88] Weighted Mean Effect Size: *d* = 0.61, [0.42, 0.79] *Post Y* vs *O*: W6m: *d =* 2.17, [1.75, 2.60], CS6m: *d =* 1.72, [1.30, 2.14]; W4m: *d =* 2.89, [2.42, 3.37], CS4m: *d =* 2.68, [2.18, 3.17]; W2m: *d =* 2.70, [2.24, 3.16], CS2m: *d =* 2.37, [1.90, 2.84] Weighted Mean Effect Size: *d* = 2.56, [2.30, 2.82] Young + Old pre‐post Weighted Mean Effect Size: *d* = 1.84, [1.61, 2.07]

*Note*: Y = young, O = old; s = second, h = hour, d = day; W = whole group, CS = correct strategy used. NCE = no calculable effect sizes for a specific method. Underscored studies = studies used for the main meta‐analysis. Underscored effect sizes = effect sizes used for calculating weighted means.

^a^ Means, standard deviations, and sample sizes.

^b^ Effect sizes reported, 95% CI calculated using formula CI = *d* ± Z*SE_*d*_.

#### The effectiveness of the method of loci compared with rehearsal in immediate serial recall in older adults

A RoBMA_PSMA_ of 1919 participants (weighted mean age = 72.78, aged 55–88) evaluated the effectiveness of the MoL based on pre‐post results in older adults in immediate serial recall. Strong evidence for a large effect size was found (*d* = 1.05, 95% CI [0.00, 1.66], [P(M|data) = 0.93, BF = 12.71]). Very strong evidence for high heterogeneity was observed (*τ* = 0.77, 95% CI [0.00, 1.66], [P(M|data) = 1, BF = 1.28 × 10^59^]), anecdotal evidence for publication bias ([(P(M|data) = 0.62, BF = 1.62]). PET‐PEESE analyses suggested the presence of small‐study effects, with PET estimating a small effect (*d* = 0.24, 95% CI [0.00, 2.79]) and PEESE a large effect (*d* = 0.91, 95% CI [0.000, 12.30]).

**FIGURE 3:**
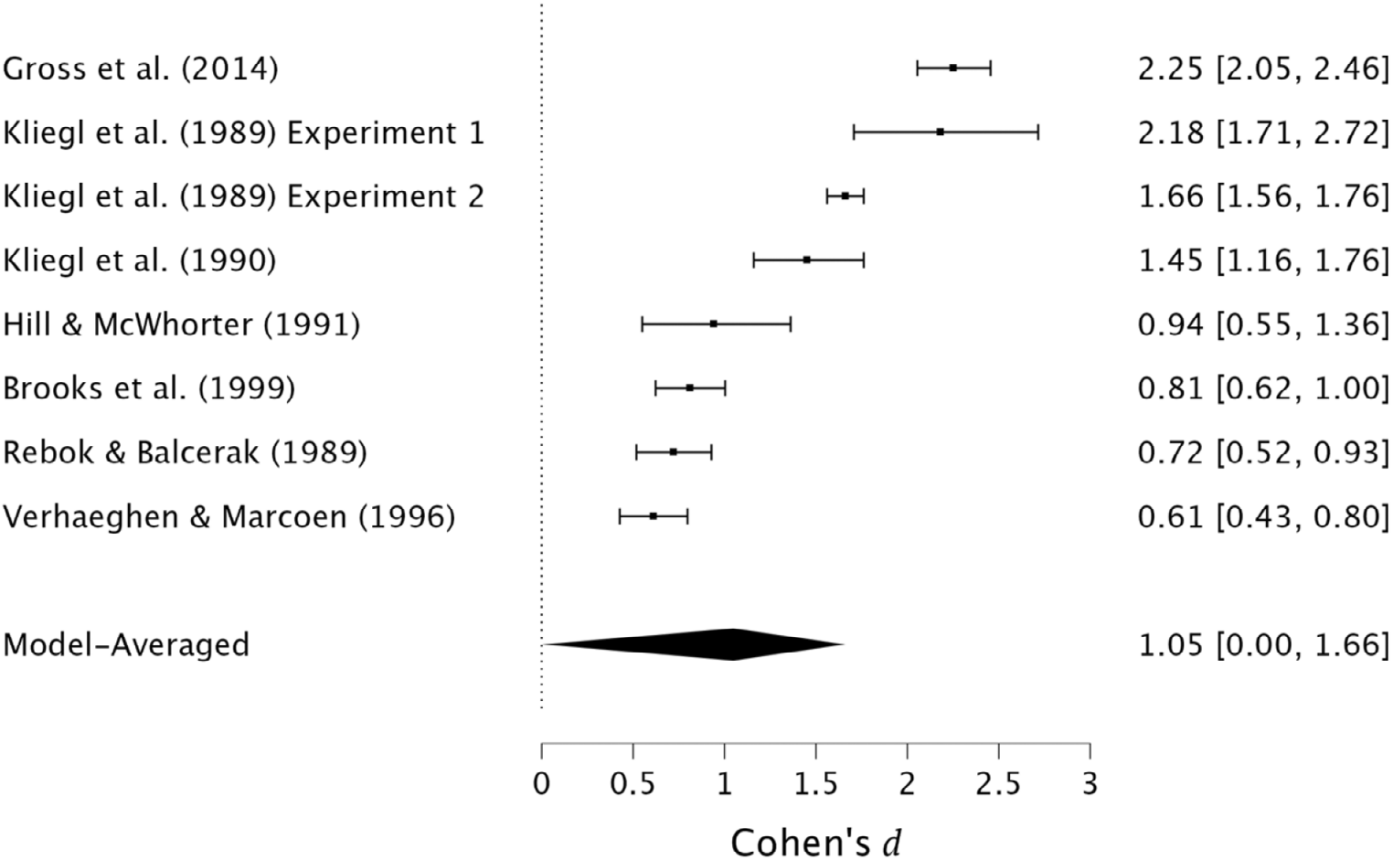
Meta‐analysis forest plot of the effectiveness of the method of loci compared with rehearsal in immediate serial recall among older adults, based on pre‐post results.

In conclusion, strong evidence for large effect size was found, with very strong evidence for high heterogeneity and anecdotal evidence for publication bias and the presence of small‐study effects. See Figure 3 for forest plots.

#### The narrative synthesis of the general effectiveness of the method of loci in older adults

Across numerous studies, the MoL has demonstrated potential for enhancing memory performance in older adults. Although it consistently improves recall, particularly when used systematically, older adults often face challenges in maintaining strategy use over time and spontaneously applying it without explicit prompts.

##### The effectiveness of the method of loci in different types of memory recall

The MoL demonstrates robust effectiveness for enhancing memory performance in older adults across different recalls and modalities.

###### Free recall

A total of six experiments focusing on free recall report clear benefits of MoL training for older adults. For instance, Robertson‐Tchabo et al. (1976) showed that older adults using MoL outperformed a control group. However, participants exhibited poor spontaneous transfer when explicit cues to use MoL were not provided. Although stronger instructions in the post‐test encouraged continued use of the strategy, performance declined compared with training sessions. Similar improvements in free recall and real‐life tasks (e.g. grocery shopping lists) were observed in Anschutz et al. (1985). Older adults who consistently used MoL maintained near‐perfect recall over a period of weeks, underscoring the importance of strategy adherence. In a long‐term follow‐up, Anschutz et al. (1987) found that although participants retained some memory of the technique after 3 years, only one individual continued to use it consistently; most showed diminished recall despite recognizing the strategy's value.

In more recent work, Boller et al. (2021) reported significant gains in free recall following MoL practice in a VR setting, although VR did not substantially enhance the transfer of these gains to new tasks. Likewise, Weintruab‐Youdkes et al. (2015) integrated music and movement into a modified MoL program, yielding robust improvements in free recall over 5 months. These enhancements, however, did not generalize to other cognitive domains (e.g. executive functioning), suggesting that MoL training primarily benefits memory.

###### Serial recall

A total of 12 experiments focusing on serial recall consistently documenting improvements among older adults. Early investigations, such as Rose and Yesavage (1983), highlighted significant gains in serial recall for young, middle‐aged, and older adults, though the magnitude of improvement was consistently lower for older participants. Further work by Yesavage and Rose (1984) demonstrated that adding semantic elaboration (e.g. pleasantness judgements alongside MoL) boosted recall durability in older adults relative to MoL alone.

Extensive ‘cognitive plasticity’ research conducted by Kliegl and colleagues underscores how, despite age‐related constraints, older adults exhibit marked improvements with MoL training. Kliegl et al. (1989) and Kliegl et al. (1990) found that while both younger and older adults benefited from MoL, age differences became more pronounced under fast presentation rates or after extended training sessions. Younger adults reached higher performance ceilings more quickly, whereas older adults continued to improve but plateaued at shorter presentation times requiring higher processing speed. Baltes and Kliegl (1992) likewise showed that even after 38 training sessions, older adults did not reach the performance levels of younger participants, suggesting neurophysiological limits on age‐related plasticity. Lindenberger et al. (1992) further revealed that older professionals (e.g. graphic designers skilled in spatial imagery) performed better than their non‐expert peers on MoL tasks but still could not match younger participants. This indicates that real‐world spatial expertise attenuates but does not eliminate age‐related declines.

Studies outside the Kliegl group confirm these findings. Rebok and Balcerak (1989) demonstrated that feedback‐based MoL training benefited both young and older adults but did not fully alleviate older adults' lower self‐efficacy beliefs. Hill et al. (1991) showed that MoL and narrative‐story strategies both significantly improved older adults’ word recall in serial recall, with gains persisting even after 3 days. Finally, Brooks et al. (1999) found that older adults (especially those over 70) who received extended MoL training exhibited greater improvements in serial recall than those given brief instruction or control treatments, indicating that protracted practice amplifies benefits.

#### Recognition

While no study examined recognition outcomes directly, Anschutz et al. (1987) assessed recognition alongside free recall in a 3‐year follow‐up. Participants who had originally learned MoL showed better recognition than recall, highlighting that even partial maintenance of the strategy can improve memory performance over extended intervals. However, as with free and serial recall, ongoing practice and strategy adherence appear essential for lasting benefits.

##### Comparison with other mnemonic methods

One study Hill et al. (1991) compared MoL against a narrative‐story mnemonic in older adults. Both techniques led to significant recall improvements over a placebo condition, and no strategy emerged as uniformly superior. This suggests that some older individuals may find narratives more intuitive while others prefer the spatial structure of MoL. Gross et al. (2014) reported that only about 25% of older adults chose to adopt MoL over simpler approaches like semantic grouping. This preference often stemmed from the perceived complexity and cognitive demands of MoL.

Modified approaches, such as adding movement and music (Weintruab‐Youdkes et al., 2015) or using VR (Boller et al., 2021), can boost engagement and concentration. However, neither study reported superior memory improvements beyond what traditional MoL training can achieve. These innovations may facilitate adherence but do not fundamentally alter MoL's effectiveness.

##### Limitations for older adults

MoL's use of structured spatial cues often reduces recall errors, such as omissions and intrusions, by providing a systematic retrieval path (e.g. Brooks et al., 1999; Gross et al., 2014; Yesavage & Rose, 1984). When participants faithfully adhered to MoL, they experienced less forgetting over time (Yesavage & Rose, 1984) and showed higher accuracy (Anschutz et al., 1985). However, older adults frequently discontinued or altered the technique (Anschutz et al., 1987; Gross et al., 2014), leading to diminished long‐term gains.

Moreover, Robertson‐Tchabo et al. (1976) and Gross et al. (2014) both noted that spontaneous application is inconsistent without explicit reminders. Many older adults find MoL cognitively demanding and are more inclined to adopt simpler strategies (Gross et al., 2014). Thus, while MoL can substantially enhance retrieval and minimize errors, consistent reinforcement and user‐friendly adaptations remain crucial.

##### Quality of evidence

We found that 55.6% of the experiments had a high risk of bias, while the remaining 44.4% showed a serious risk of bias. There is very strong evidence of high heterogeneity, anecdotal evidence of publication bias, and signs of small‐study effects in selected studies. According to the GRADE evaluation, the overall quality of evidence in older adults is very low – primarily due to limitations in study design and the prevalence of serious and high risk of bias. Furthermore, several studies report high attrition rates and inconsistent strategy adherence, which complicate longitudinal assessments (e.g. Anschutz et al., 1987; Gross et al., 2014).

##### The combined conclusion of qualitative and narrative syntheses

Collectively, results from the quantitative and qualitative synthesis indicate that the method of loci is a potent mnemonic for improving both free and serial recall, showing clear advantages over rehearsal – particularly with strong evidence of a large effect size in immediate serial recall among older adults.

Nevertheless, age‐related declines in processing speed and increased cognitive demands constrain the extent of these improvements, and the method's complexity often limits spontaneous adoption. While extended practice, explicit prompts, and semantic elaboration can enhance recall durability and reduce errors, older adults typically still do not reach the performance levels of younger adults.

#### Meta‐analyses synthesizing the effectiveness of the method of loci in immediate serial recall among young, older, and all adults

To enhance the robustness of our findings, we conducted additional analyses using data from young adult subgroups embedded within studies primarily focused on older adults (i.e. Kliegl et al., 1989, 1990; Lindenberger et al., 1992; Rebok & Balcerak, 1989; Verhaeghen & Marcoen, 1996). Including these subgroups allowed for a broader and more comprehensive evaluation of the MoL's effectiveness.

##### A meta‐analysis of leveraged data from young adult subgroups in older adult–focused research compared with rehearsal in immediate serial recall

A RoBMA_PSMA_ of 151 participants (weighted mean age = 19.85, aged 17–29) evaluated the effectiveness of the method of loci in young adult subgroups within older adult‐focused research, compared with rehearsal in immediate serial recall. The analysis found a moderate evidence for a large effect (*d* = 1.03, 95% CI [−0.44, 2.62], [P(M|data) = 0.79, BF = 3.66]). Very strong evidence was found for high heterogeneity (*τ* = 1.88, 95% CI [0.62, 4.89], [P(M|data) = 1, BF = 3.32 × 10^114^]), with moderate evidence for publication bias [P(M|data) = 0.88, BF = 7.38]. PET‐PEESE analyses suggested the presence of small‐study effects, with PET estimating a large effect (*d* = 1.39, 95% CI [0.00, 8.01]) and PEESE also estimating a large effect (*d* = 2.26, 95% CI [0.00, 11.50]).

In conclusion, moderate evidence for large effect size was observed, with very strong evidence for high heterogeneity and moderate evidence for publication bias. Although PET‐PEESE analyses gave estimates that are numerically higher than the unadjusted effect, their very wide credible intervals signal substantial uncertainty. More importantly, the divergence between these estimates indicates that small‐study effects are present.

**FIGURE 4:**
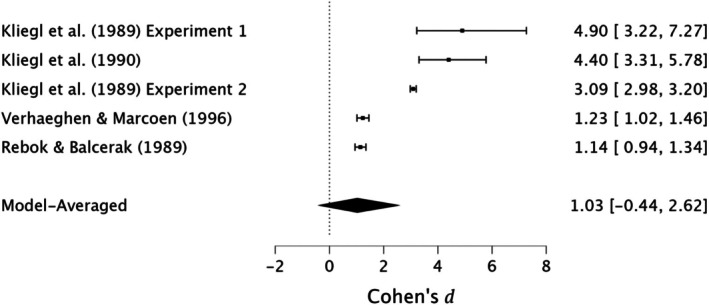
Meta‐analysis forest plot of the effectiveness of the method of loci in young adult subgroups within older adult–focused research, compared with rehearsal in immediate serial recall.

See Figure 4 for forest plots.

##### A meta‐analysis of the effectiveness of the method of loci for both young adults and young adult subgroups in older adult‐focused research compared with rehearsal in immediate serial recall

A RoBMA_PSMA_ of 1087 participants (weighted mean age = 19.96, aged 17–44) evaluated the effectiveness of the MoL for both young adults and young adult subgroups in older adult‐focused research compared with rehearsal in immediate serial recall. Strong evidence for a medium effect was found (*d* = 0.75, 95% CI [0.00, 1.33], [P(M|data) = 0.93, BF = 12.26]). Very strong evidence for high heterogeneity (*τ* = 0.946, 95% CI [0.63, 1.44], [P(M|data) = 1, BF = ∞), and very strong evidence for publication bias ([P(M|data) = 1, BF = 640440.49]), was observed. PET‐PEESE analyses indicated the presence of small‐study effects, with PET estimating a small effect (*d* = 0.22, 95% CI [0.00, 6.22]) and PEESE estimating a larger effect (*d* = 8.45, 95% CI [0.00, 11.35]).

**FIGURE 5:**
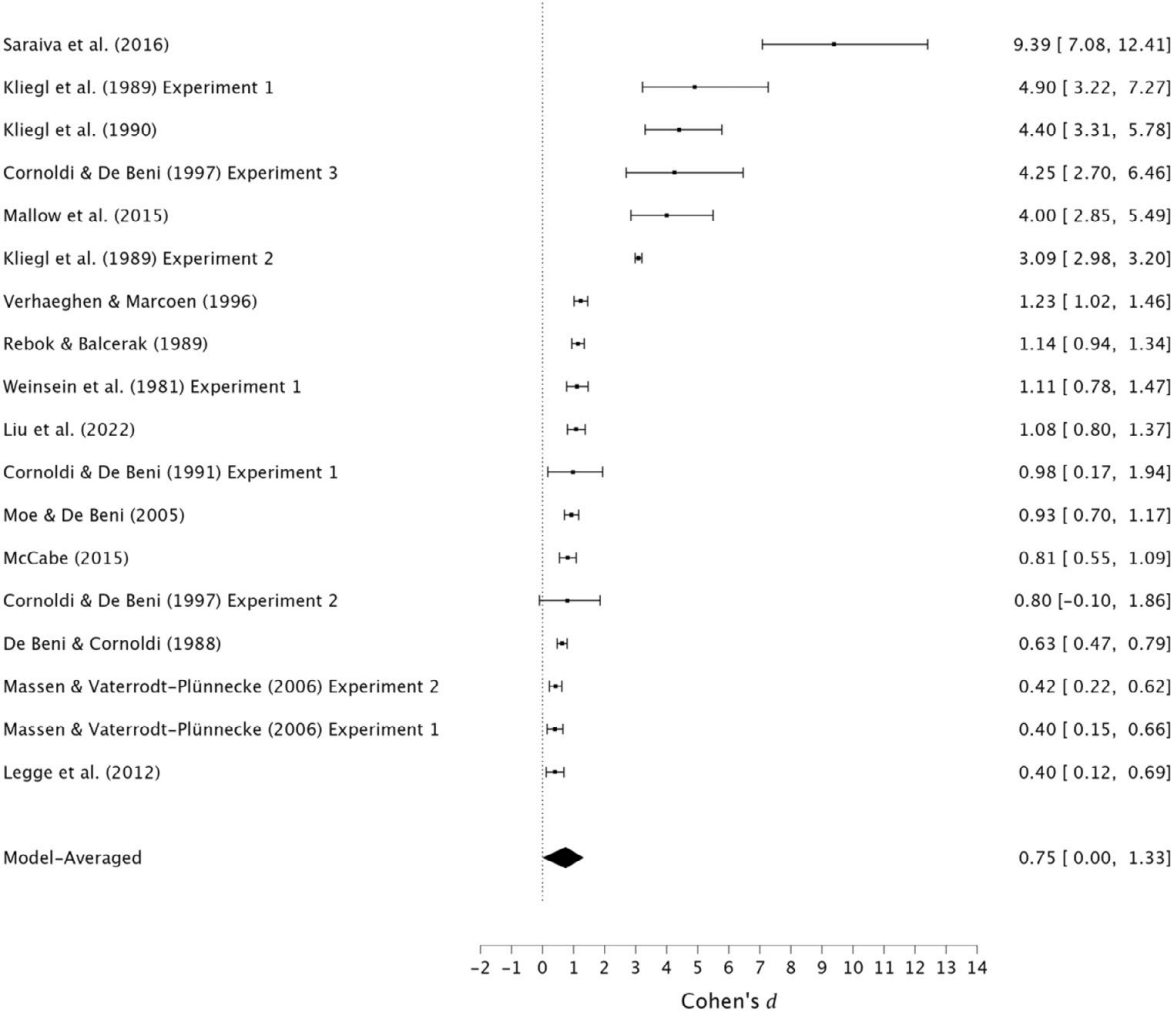
Meta‐analysis forest plots of the effectiveness of the method of loci for both young adults and young adult subgroups in older adult‐focused research compared with rehearsal in immediate serial recall.

In conclusion, there is strong evidence of a medium effect size, accompanied by very strong evidence of high heterogeneity, publication bias, and the presence of small‐study effects. See Figure 5 for the corresponding forest plots.

##### A meta‐analysis of the effectiveness of the method of loci in comparing young adults and older adults

A RoBMA_PSMA_ of 354 participants (weighted mean age = 47.66, aged 17–87) evaluated the effectiveness of the MoL compared with rehearsal in immediate serial recall, comparing young adult subgroups in older adult‐focused research to older adults. Very strong evidence of a large effect was found (*d* = 2.30, 95% CI [1.37, 2.82], [P(M | data) = 0.996, BF = 221.69]). Very strong evidence for moderate heterogeneity was observed (*τ* = 0.54, 95% CI [0.23, 1.27], [P(M|data) = 1, BF = 6.21 × 10^5^]). Anecdotal evidence for publication bias was found [P(M|data) = 0.58, BF = 1.39]. PET‐PEESE suggested the presence of small‐study effects, with PET estimating a small effect (*d* = 0.48, 95% CI [0.00, 4.22]) and PEESE estimating a medium effect (*d* = 0.71, 95% CI [0.00, 8.32]).

**FIGURE 6:**
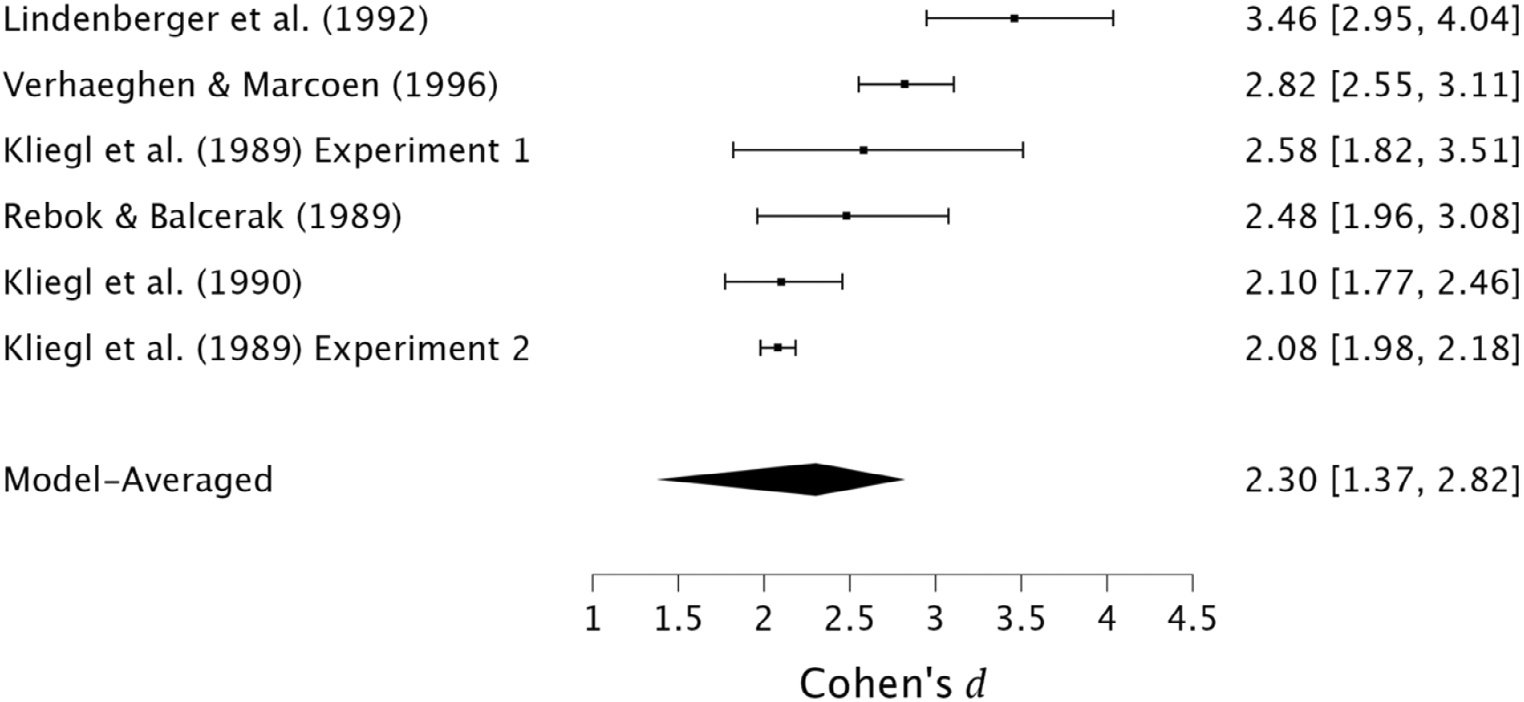
Meta‐analysis forest plots of the effectiveness of the method of loci compared with rehearsal in immediate serial recall, comparing young adult subgroups in older adult‐focused research to older adults.

In conclusion, very strong evidence supports a large effect of the MoL when comparing young and older adults in immediate serial recall, though this is accompanied by very strong evidence of moderate heterogeneity, anecdotal evidence of publication bias, and indications of small‐study effects. See Figure 6 for forest plots.

##### Meta‐analysis of the effectiveness of the method of loci in young adults, young adult subgroups in older adult–focused research, and older adults (combined adult sample), compared with rehearsal in immediate serial recall

A RoBMA_PSMA_ of 3006 participants (weighted mean age = 60.94, aged 17–88) evaluated the effectiveness of the MoL in young adults, young adult subgroups within older adult–focused research, and older adults (i.e. the combined adult sample), compared with rehearsal, in immediate serial recall. Very strong evidence of a large effect was found (*d* = 0.88, 95% CI [0.47, 1.25], [P(M|data) = 0.99, BF = 161.94]). Very strong evidence for high heterogeneity was observed (*τ* = 0.772, 95% CI [0.54, 1.11], [P(M|data) = 1, BF = 1.026 × 10^301^]), and very strong evidence for publication bias was found ([P(M|data) = 1.000, BF = 2.97 × 10^6^]). PET‐PEESE analyses indicated the presence of small‐study effects, with PET estimating a negligible effect (*d* = 0.04, 95% CI [0.00, 0.00]) while PEESE produced an implausibly large estimate (*d* = 8.24, 95% CI [5.87, 10.55]).

**FIGURE 7:**
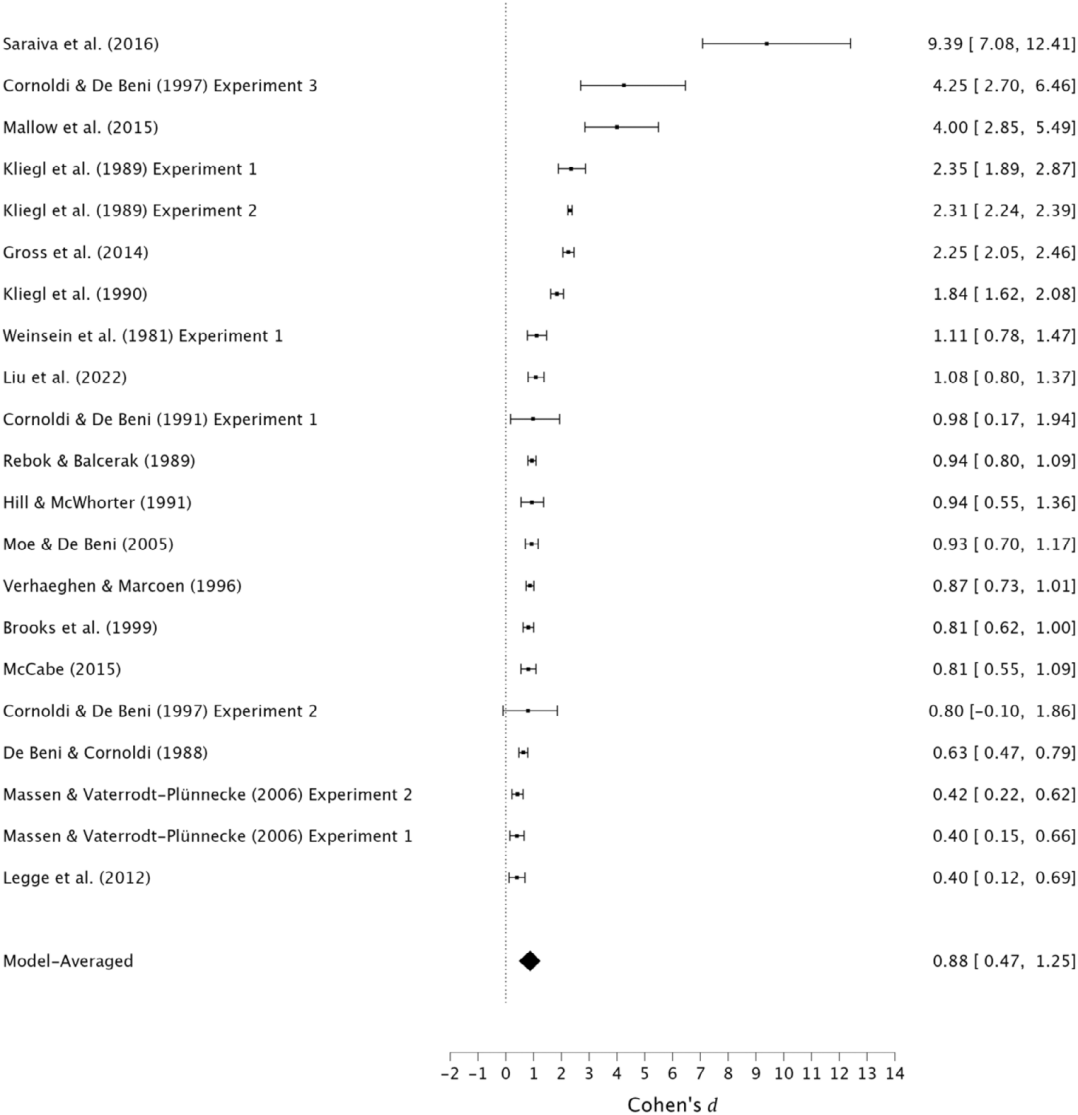
Meta‐analysis forest plots of the effectiveness of the method of loci in young adults, young adult subgroups in older adult–focused research, and older adults (combined adult sample), compared with rehearsal in immediate serial recall.

Although there is very strong evidence for a large effect of the MoL on immediate serial recall in adults, the presence of high heterogeneity, substantial publication bias, and clear indications of small‐study effects mean that the pooled estimate should be interpreted with caution (Figure 7).

##### The meta‐analytic conclusion

Meta‐analytic syntheses revealed relatively robust evidence supporting the effectiveness of the MoL in enhancing immediate serial recall across young adults, older adults, and combined samples. Across all analyses, effect sizes ranged from small to large and were consistently accompanied by very strong evidence of moderate to high heterogeneity. Publication bias was also prevalent, with evidence ranging from anecdotal to very strong, and PET‐PEESE analyses consistently indicated small‐study effects that likely inflated the true effect sizes. Collectively, these findings confirm the general efficacy of the MoL in improving memory performance but underscore the need for great caution in interpreting these results.

### The narrative synthesis of cognitive mechanisms underlying the effectiveness of the method of loci

The MoL is a mnemonic technique in which to‐be‐remembered items are associated with well‐known locations arranged along a familiar route, thus forming a structured, spatial memory palace. During recall, individuals mentally traverse this route, utilizing spatial cues to sequentially retrieve the encoded information. The levels of processing framework proposed by Craik and Lockhart (1972) provides a comprehensive theoretical perspective on the effectiveness of the MoL. According to this framework, deeper and more meaningful encoding processes result in stronger and more enduring memory traces. Within the context of the MoL, several cognitive mechanisms operate collectively to achieve this depth of processing, enhancing memory performance.

For clarity, in Table 18, we present the extracted characteristics of the MoL that formed the basis for including the cognitive mechanisms described in the narrative synthesis.

**TABLE 18:** The extracted characteristics of the method of loci that make it effective.

Study	Findings 1	Findings 2	Findings 3
Bellezza and Reddy (1978)	Subject‐generated loci better than experimenter‐generated	Conscious awareness of environment not necessary for recall	Primacy effect present
Blunt and VanArsdall (2021) Experiment 1	Animate words recalled more than inanimate ones	Animacy effect smaller in MoL group	
Blunt and VanArsdall (2021) Experiment 2	Animate images enhance recall for both animate and inanimate words	No difference between animate/inanimate word recall when paired	
Briggs et al. (1970)	Bizarre images enhance recall	Self‐created bizarre associations not necessary	Self‐reference elimination does not affect recall
Canelloupoulou and Richardson (1992)	Executive processing capacity necessary for effective MoL	Depression scores non‐influential	Experimenter‐generated imagery better for those with low capacity
Caplan et al. (2019)	Imagined spatial navigation not essential for MoL	MoL undistinguished among Peg methods	Apartment environment better than others for recall
Crovitz (1969)	Vivid, bizarre images at specific locations enhance recall	Pre‐known locations are not necessary for the MoL effectiveness	Newly presented locations do not reduce recall
Kemp and Van der Krogt (1985) Experiment 1	Recall worse with visible loci during learning, not recall	Loci consistency (visible/imagined) key to recall performance	Consistency needed across learning and recall
Kemp and Van der Krogt (1985) Experiment 2	Recall worse with visible loci during learning	Loci consistency is key to performance	Imagined loci can be as effective as physical ones
Lea (1975) Experiment 1	Linear relationship between loci scanned and reaction time	Distance hypothesis unsupported	RETRIEVE process independent of MOVE, time varies with familiarity
Lea (1975) Experiment 2	MOVE slower in unfamiliar environments	Distance hypothesis unsupported	MOVE and RETRIEVE processes are independent
Lea (1975) Experiment 3	Linear reaction time with loci scanned	Distance hypothesis unsupported	MOVE and RETRIEVE processes are independent
Massen et al. (2009) Experiment 1	Street routes more effective than house locations	Path complexity may reduce recall	Benefits of bizarreness unclear
Massen et al. (2009) Experiment 2	Street routes more effective than house locations	Type of item (grocery/house) does not affect path efficacy	Bizarreness effects unclear
Reggente et al. (2020)	Spatial binding enhances recall	No effect of environment type on recall	Spatial memory crucial to MoL

#### Dual coding theory

According to dual coding theory (Paivio & Csapo, 1973), forming both visual and verbal representations of information results in stronger and more durable memory traces.

MoL naturally activates these dual channels, prompting learners to create vivid mental imagery of each item while simultaneously retaining its verbal label. Three studies highlight the importance of distinctive visual representations. Crovitz (1969) demonstrated that bizarre and vivid images significantly enhance recall. Similarly, Briggs et al. (1970) showed that even when self‐referential processing is minimized, vivid or bizarre imagery alone substantially boosts memory performance. More recently, Blunt and VanArsdall (2021) found that animate imagery, compared with inanimate imagery, further enhances recall, underscoring the robustness of imagery‐based strategies inherent to MoL. Collectively, these findings emphasize how visual distinctiveness substantially contributes to memory enhancement within the MoL framework.

#### Self‐reference, emotional context enhancement, and autobiographical memory

The method of loci leverages personal relevance and emotional context to enhance encoding and recall, drawing upon mechanisms such as the self reference effect (Rogers et al., 1977), emotional context enhancement (Hamann, 2001), and autobiographical memory processes (Chen et al., 2017).

For instance, Bellezza and Reddy (1978) reported superior recall when participants used personally familiar locations as opposed to unfamiliar or arbitrary ones. In this regard, the MoL activates autobiographical memory networks by prompting learners to visualize personally familiar locations and routes.

Moreover, Massen et al. (2009) reported superior recall when participants utilized external, real‐world routes rather than internal, house‐based loci. This advantage likely arises because external routes evoke richer, more distinctive, and often emotionally charged associations between to‐be‐remembered items and locations – driven by the unconventionality, novelty, bizarreness, and vividness of the mental imagery they elicit.

Furthermore, by prompting individuals to create personal associations both among the items themselves and with specific locations, the MoL effectively integrates the self reference effect and autobiographical memory mechanisms, reinforcing the encoding process.

#### Context‐dependent memory

Another mechanism underlying the effectiveness of MoL is context‐dependent memory (Godden & Baddeley, 1975). This phenomenon describes how returning to the same context at retrieval as during encoding enhances recall performance.

In the MoL, users create a memory palace using well‐known locations. During encoding, learners mentally traverse their memory palace, forming associations between locations and items to be remembered. Later, during recall, learners mentally revisit the same locations, effectively reinstating the original encoding context. This consistency between encoding and retrieval contexts parallels the principle that memory performance improves when external or internal cues match those present during initial learning.

By design, MoL systematically reconstructs contextual cues – each locus within the memory palace – thereby optimizing retrieval through context‐dependent memory. Kemp and Van der Krogt (1985) demonstrated the importance of consistency in locus representation, showing that altering loci from visible to imagined between encoding and recall can disrupt memory performance. Supporting this, Essoe et al. (2022) in their study not directly focusing on MoL, found that learning in distinctive VR environments enhanced retention, particularly when those contexts were mentally reinstated during recall. Importantly, this benefit was strongest for participants who reported a high sense of presence, suggesting that the subjective realism of the learning context enhances the effectiveness of reinstatement. Neural data further revealed that reinstating brain activity patterns associated with the original context predicted successful retrieval.

#### Desirable difficulty

The concept of desirable difficulty (Bjork, 1994) further clarifies why the MoL is effective, suggesting that learning tasks requiring substantial yet desirable cognitive effort lead to enhanced recall. The MoL inherently involves effortful mental processes, such as generating elaborate visual imagery, directing attention, maintaining sequential organization, working within a complex spatial scaffold, and consistently reviewing mental associations during recall.

However, these cognitive demands must be balanced against individual capacities. Canellopoulou and Richardson (1998) underscored this point by demonstrating that individuals with impaired executive functions, such as patients with multiple sclerosis, benefited more from experimenter‐generated imagery than from self‐created imagery. This finding highlights that in the context of the MoL, excessive cognitive load can impair memory performance rather than enhance it, particularly among individuals with limited executive functioning capacities.

#### Elaborative processing and long‐term working memory

Elaborative processing (Craik & Tulving, 1975) refers to a deep encoding strategy involving the active connection of new information to pre‐existing knowledge. This process enhances memory by forming rich, interconnected mental representations that significantly improve recall.

In the context of the MoL, each locus functions as an anchor point for encoding to‐be‐remembered items, leveraging deeply embedded spatial cues within a well‐known environment. The resulting memory palace, constructed from known and familiar locations, provides a robust spatial scaffold of pre‐existing knowledge, offering strong, stable cues that facilitate efficient retrieval.

Ericsson and Kintsch's (1995) long‐term working memory (LT‐WM) model also provides a useful theoretical perspective on the efficacy of the MoL. According to this model, experts can store large quantities of information within long‐term memory while ensuring swift and effective retrieval through established retrieval structures.

Within the MoL, the familiar route and its corresponding loci serve precisely this function. Each locus acts as a retrieval cue that can be temporarily held in short‐term memory to enable immediate reactivation of the associated item stored in long‐term memory. Furthermore, the MoL may be resilient to interruptions because it relies on stable memory traces established in long‐term memory. By sequentially revisiting each locus, users can resume recall even after attention is diverted. Thus, memory traces are not lost from a short‐term store; rather, they are simply reactivated by moving to the next locus.

#### Spatial binding

Spatial binding, the cognitive process of associating items with specific mental locations, seems to be the core mechanism underlying the effectiveness of the MoL.

Reggente et al. (2020) demonstrated that explicitly binding objects to fixed locations within fantastical virtual environments consistently improved both serial and free recall performance, with moderate effect sizes observed in forward serial and reverse recall tasks.

Lea (1975) originally demonstrated that a stable, consistent mental arrangement of locations significantly enhances recall, emphasizing its importance over literal mental navigation or the physical distances between loci. Caplan et al. (2019) noted that the primary advantages of MoL arise from imagery‐based strategies rather than active spatial navigation, comparing its effectiveness to Pegword mnemonic techniques.

Moreover, VR could serve as a valuable tool to enhance experimental control in MoL research. Reggente et al. (2018), although not specifically examining MoL, demonstrated that VR increases ecological validity by providing immersive, standardized environments closely resembling real‐world contexts. Additionally, Reggente (2023) showed that VR effectively bridges the gap between laboratory‐based assessments and everyday memory performance. Supporting this idea, Krokos et al. (2019) found that virtual memory palaces presented through head‐mounted displays significantly improved recall performance compared with traditional desktop‐based displays.

Consequently, adopting VR‐enhanced methods could improve experimental control by ensuring that all participants use, for example, identical standardized memory locations instead of highly variable individually chosen ones.

#### Quality of evidence

Nevertheless, 80% of the experiments were assessed as having a high risk of bias, 6.67% as having a serious risk of bias, and the remaining 13.3% were judged to have some concerns, including inconsistencies in results (e.g. reliance on bizarre imagery). Using the GRADE approach, the body of evidence for the cognitive mechanisms underlying the effectiveness of the MoL was rated as very low quality, primarily due to high risk of bias and the nature of the experimental designs. Although some findings are consistent across studies, methodological flaws and imprecision significantly reduce confidence in the overall body of evidence.

#### Conclusion

Taken together, the narrative synthesis underscores that the MoL unites several complementary cognitive mechanisms, many of which fall under the broader framework of the levels of processing theory, including dual coding theory, emotional context enhancement, self reference effect, autobiographical memory, context‐dependent memory, desirable difficulty, elaborative processing, and long‐term working memory.

By simultaneously engaging verbal and visual channels, leveraging personal and emotional significance, and anchoring information within well‐known spatial routes, the MoL fosters deeper levels of encoding that reinforce memory retention. Spatial binding and long‐term working memory further complement this multifaceted encoding process by supporting stable and easily reactivated mental frameworks.

Although the precise contribution of each mechanism may vary based on individual differences and task demands, their combined influence consistently emerges as a powerful foundation for the MoL's mnemonic efficacy.

### Narrative synthesis of the method of loci in the context of neuropsychology

#### Spatial encoding: Hippocampal, parahippocampal and retrosplenial networks

Neuroimaging studies have consistently identified several key brain regions involved in the MoL, specifically those essential for spatial memory, visualization, and navigation. The hippocampus, especially the right posterior hippocampus, plays a critical role in supporting the spatial memory and navigational processes central to the MoL (Maguire et al., 2003). Müller et al. (2018) further emphasize the cooperative activity between the hippocampus and the caudate nucleus observed in memory athletes. Liu et al. (2022) provided additional insights by demonstrating that hippocampal CA1 and CA23DG subfields actively encode and retrieve temporal order and sequence boundaries, while highlighting the parahippocampal cortex as crucial for spatial encoding. Activity in the parahippocampal gyrus and cortex during memory encoding supports scene construction and memory consolidation processes (Fellner et al., 2016; Kondo et al., 2005; Mallow et al., 2015).

Although the following studies did not explicitly test MoL, recent neuroimaging and VR research strongly supports the method's effectiveness by showing that anchoring items to spatial locations robustly engages the hippocampus and parahippocampal cortex during navigation and recall tasks (Reggente, 2023). VR‐based studies, in particular, have demonstrated that even brief immersive interactions, such as actively exploring or virtually placing items within scenes, can significantly activate hippocampal and parahippocampal regions, thus reinforcing the importance of spatial memory networks during encoding. Robin et al. (2018) similarly found that spatial context serves as a fundamental organizing principle for episodic memory representation, indicating that the brain primarily distinguishes events based on location. Their findings implicated not only the parahippocampal and posterior hippocampal regions but also involved the retrosplenial cortex, posterior cingulate cortex (PCC), and precuneus in spatially guided recall. Together, these studies illustrate how spatial context, whether imagined, real, or virtual, can effectively scaffold memory formation and retrieval, aligning closely with the foundational premise of MoL.

The retrosplenial cortex appears to be a key brain region that contributes to spatial navigation and recall when using the method of loci (Kondo et al., 2005; Nyberg et al., 2003; Wagner et al., 2021). In a different study, Reggente et al. (2018) further underscored how spatial memory tasks, especially those using VR, recruit the hippocampus and retrosplenial cortex, and can elicit grid‐cell‐like coding in the entorhinal cortex, revealing parallels to rodent navigation and reinforcing the idea that forming robust ‘scene‐based’ memory traces helps recall. Similarly, Robin et al. (2016) reported that individuals naturally and preferentially utilize spatial context to aid memory retrieval, elucidating why spatial mnemonic strategies such as MoL can be particularly effective. Additionally, Benn et al. (2015) demonstrated that even navigating digital folder structures activates a network including the retrosplenial cortex, parahippocampal gyrus, cingulate gyrus, lingual gyrus, precuneus, and superior parietal lobule (SPL), regions commonly activated during real‐world navigation. While their study was not specifically centred on MoL, these findings confirm that spatially guided retrieval consistently relies on neural networks also utilized by MoL.

#### Executive processes in encoding and recall

Applying MoL also involves higher level executive processes and frontal‐lobe engagement. The dorsolateral prefrontal cortex (DLPFC), particularly on the right side, plays a significant role in organizing and monitoring the MoL (Belleville et al., 2023; Nyberg et al., 2003; Raz et al., 2009). Dresler et al. (2017) found that following MoL training, connectivity in the DLPFC reorganizes to support more efficient memorization. Additionally, the inferior and middle frontal gyri appear to play a role in linking objects with their corresponding loci (Kondo et al., 2005).

Brain regions associated with vision and imagery significantly contribute to MoL performance as well. The fusiform gyrus, which is critical for object recognition, exhibits heightened activation and structural cortical changes during both encoding and recall phases when using MoL (Engvig et al., 2010; Kondo et al., 2005). The lingual gyrus and visual cortex facilitate visualization processes essential for the method (Kondo et al., 2005; Mallow et al., 2015), while the cingulate gyrus and PCC support attentional modulation and episodic memory retrieval (Dresler et al., 2017; Kondo et al., 2005). The medial prefrontal cortex and SPL further bolster spatial attention and memory consolidation (Dresler et al., 2017; Liu et al., 2022).

#### Cognitive plasticity, learning strategies, and age

From a functional perspective, mnemonic training can reorganize large‐scale brain networks (Dresler et al., 2017). For example, training with the MoL can enhance neural efficiency and strengthen functional connectivity between the hippocampus and neocortical areas (e.g. DLPFC, angular gyrus), thereby supporting stable long‐term memory (Wagner et al., 2021). Structural changes in grey and white matter associated with MoL use, particularly in the right fusiform gyrus and hippocampal cingulum, have also been linked to performance gains (Engvig et al., 2010). de Lange et al. (2018) likewise documented structural alterations associated with consistent mnemonic practice. Fellner et al. (2016) noted that reduced theta power in the medial temporal lobe contributes to more optimal encoding.

Additionally, expert memorizers may sometimes rely less on direct spatial navigation, instead favouring linguistic or sequential strategies when using MoL (Mallow et al., 2015).

Finally, older adults benefit from MoL training by recruiting occipito‐parietal regions as well as the retrosplenial cortex (Belleville et al., 2023; Nyberg et al., 2003).

#### Quality of evidence

However, 20% of the included experiments were judged to have a high risk of bias, 6.7% exhibited a serious risk of bias, 20% had some risk, and 53.3% had a moderate risk. Applying the GRADE approach, we assess the overall confidence in the evidence as low, primarily due to concerns regarding risk of bias and study design, particularly the limited number of RCTs.

#### Conclusion

Overall, the evidence consistently converges on a central principle. The MoL leverages the brain's natural predisposition to anchor memories within spatial contexts, drawing upon an extensive neural network that prominently includes the hippocampus, parahippocampus, retrosplenial cortex, as well as additional frontal and parietal regions. Specifically, areas involved in scene construction and spatial navigation – particularly the parahippocampal gyrus, retrosplenial cortex, and posterior hippocampus – activate when real or imagined locations serve as mnemonic anchors. Higher order executive and visual‐processing regions, such as the DLPFC, fusiform gyrus, and lingual gyrus, further contribute by managing attentional control, supporting detailed imagery, and binding information precisely to designated loci.

Beyond simply enhancing memory performance, training with MoL induces significant neural plasticity, strengthening hippocampal–cortical connectivity and reducing medial temporal theta power during encoding. For a summary of frequently identified brain regions, see Table 19.

**TABLE 19:** The method of loci in the context of neuropsychology: first 10 key brain regions frequently identified as important and arranged by citation count.

Merged brain regions	Mentioned as important by	Citation count (*N*/12)	Region's general function
Retrosplenial cortex	↑^U^ Maguire et al. (2003), ↑^E^ Nyberg et al. (2003), ↑^R^ Kondo et al. (2005), ↑^E^ Mallow et al. (2015), ↑^C^ Dresler et al. (2017), ↓^O^ Wagner et al. (2021)	6	Retrosplenial cortex is primarily involved in spatial navigation, path integration, landmark processing, viewpoint transformation; posterior RSC in navigation, scene processing; anterior RSC in episodic memory, mental imagery, self‐referential; left RSC in episodic memory; right RSC in spatial processing, perspective shifting, prediction generation/updating (Alexander et al., 2023; Chrastil, 2018; Mitchell et al., 2018; Vann et al., 2009)
Parahippocampus	↑^R^ Kondo et al. (2005), ↑^E^ Mallow et al. (2015), ↓↑^O^↑^C^ Wagner et al. (2021), ↑^UR^ Fellner et al. (2016), ↑^C^ Dresler et al. (2017), ↑^E^ Liu et al. (2022)	6	PHC is primarily involved in contextual associative processing, plays a crucial role in both spatial and non‐spatial cognitive tasks, including episodic memory, visuospatial processing, and emotional stimuli processing. Specifically, it helps in linking items with their contextual associations, such as object‐place pairings in memory or scenes and environments in visuospatial tasks (Aminoff et al., 2013)
Cingulate cortex	↑^U^ Maguire et al. (2003), ↑^ER^ Kondo et al. (2005), ↑↓^ER^↑^V^ Raz et al. (2009), ↑^C^ Dresler et al. (2017), ↑^U^ Fellner et al. (2016), ↓^R^ Belleville et al. (2023)	6	The posterior cingulate cortex (PCC) is a key part of the default mode network, involved in internally directed cognition like autobiographical memory, future planning, and attention control (Leech & Sharp, 2014). The anterior cingulate cortex (ACC) detects task conflicts, regulates control effort, and balances rewards versus cognitive control costs. It helps adjust behaviour based on past performance and adapts control intensity based on task demands (Shenhav et al., 2013). Both regions optimize cognitive control and attention management
Hippocampus	↑^U^ Maguire et al. (2003), ↑^V^ Müller et al. (2018), ↓^O^↑^C^ Wagner et al. (2021), ↑↓^ER^ Raz et al. (2009), ↑^ER^ Liu et al. (2022), ↑^T^de Lange et al. (2018)	6	The human hippocampus is involved in several cognitive functions, including declarative and working memory, semantic and conceptual relationships, coding associations, and spatial navigation (Quian Quiroga, 2023). It also plays a role in tracking contextual continuity, which helps maintain a sense of ‘sameness’ in an environment despite continuous sensory changes (Maurer & Nadel, 2021), and has a temporally graded function in autobiographical memory retrieval (Gilmore et al., 2021)
Fusiform gyrus	↑^U^ Maguire et al. (2003), ↑^ER^ Kondo et al. (2005), ↓^O^ Wagner et al. (2021), ↑^E^ Raz et al. (2009), ↑^T^ Engvig et al. (2010)	5	The fusiform gyrus is key in processing high‐order visual information, especially for facial recognition, bodies, and high spatial frequency stimuli. The right fusiform face area (FFA) is linked to facial perception, with stimulation causing facial distortions. Its connections to the lingual and inferior occipital gyri further support its role in broader visual processing (Palejwala, O'Connor, Milton, et al., 2020; Palejwala, O'Connor, Pelargos, et al., 2020)
Middle frontal gyrus	↓^R^ Belleville et al. (2023), ↑^E^ Kondo et al. (2005), ↑^E^ Fellner et al. (2016), ↑^E^ Wagner et al. (2021), ↑^E^ Raz et al. (2009)	5	The middle frontal gyrus (MFG) is involved in attention, working memory, and language‐related processing (Briggs et al., 2021). The middle frontal gyrus helps shift attention between goal‐directed and stimulus‐driven processes, acting as a ‘circuit breaker’ to reorient focus to unexpected stimuli (Japee et al., 2015)
Inferior frontal gyrus	↑^E^ Fellner et al. (2016), ↑^E^ Wagner et al. (2021), ↓^R^ Belleville et al. (2023), ↑^E^ Kondo et al. (2005), ↑^U^ Maguire et al. (2003)	5	The inferior frontal gyrus (IFG) supports language processing, working memory, motor control, and empathy. The left IFG handles semantic and phonological processing (linked to Broca's area) and is active in working memory tasks, while the right IFG manages fine motor control. The left IFG also processes empathy, connecting action perception with emotional understanding. The IFG plays a key role in language, memory, motor control, and social cognition (Liakakis et al., 2011)
Orbitofrontal cortex	↑^E^ Raz et al. (2009), ↓^O^ Wagner et al. (2021), ↑^T^ Engvig et al. (2010), ↑^E^ Liu et al. (2022), ↓^R^ Belleville et al. (2023)	5	The orbitofrontal cortex (OFC) decodes and represents the reward value of primary reinforcers such as taste and touch, and helps learn and reverse associations between these and other stimuli, including visual, auditory, and abstract cues like monetary rewards. It controls reward‐ and punishment‐related behaviour, crucial for emotion regulation. The OFC predicts reward values and correlates with subjective emotions. Damage impairs emotional learning and behaviour. The medial prefrontal cortex (area 10) aids in binary decision‐making. OFC activity is influenced by attention to affect, while stimulus intensity affects earlier cortical areas (Rolls, 2004; Rolls & Grabenhorst, 2008)
Superior parietal cortex	↑^U^ Maguire et al. (2003), ↑^E^ Mallow et al. (2015), ↑^E^^,^ ^a^ Fellner et al. (2016), ↑^R^ Liu et al. (2022), ↓^O^ Wagner et al. (2021)	5	The superior parietal lobule (SPL) plays a pivotal role in many sensory and cognitive processes, including somatosensory and visuomotor integration, spatial perception, mental rotation, visuospatial attention and memory (Wang et al., 2015). It is also critically important for the manipulation of information in working memory (Koenigs et al., 2009)
Occipital cortex	↑^U^ Nyberg et al. (2003), ↑^U^ Fellner et al. (2016), ↑^R^ Liu et al. (2022), ↓^E^ Raz et al. (2009)	4	The occipital lobe has significant role in visuospatial cognition. The dorsal and ventral streams are essential for spatial location and object recognition, respectively (Palejwala, O'Connor, Milton, et al., 2020)

*Note*: ↑ = Increase, ↓ = decrease (mainly in activity), E = encoding, R = recall, U = (while) using the method, T = cortical thickness of a region tied to using the method, V = volume of a brain region tied to using the method, C = higher connectivity in the brain region while using the method, O = other tasks (mostly temporal order recognition) (for example ↑^E^ = increase brain activity while encoding, ↑^V^ increased volume of a brain regions tight to using the method, ↑^ER^ = increase activity while both encoding and recalling ↓↑^O^↑^C^ = increas and decrease of activity in other tasks and increase of connectivity of a region while using the method).

^a^ Alpha/beta power increase for mnemonic processing and positive SMEs with peak in superior parietal gyrus.

### Miscellaneous studies outside the scope of the current review

There are also several noteworthy studies employing the MoL in clinical contexts and other diverse applications that merit attention. Although these studies lie outside the primary scope of the current systematic review, they illustrate the broader versatility and practical utility of MoL in enhancing memory performance across diverse populations, settings, and conditions, thus providing a more comprehensive perspective on the method and integrating the complete body of evidence‐based experimental research conducted since 1968.

#### Studies with clinical context

Sousa et al. (2021) found the MoL effective for enhancing episodic memory in healthy individuals but not in individuals with schizophrenia. Dalgleish et al. (2013) demonstrated that MoL significantly improves memory retention of self‐affirming memories in people with depression. Similarly, Werner‐Seidler and Dalgleish (2016) showed the method's efficacy in helping individuals with recurrent depression maintain positive memories and enhance mood. Ruchkin et al. (2024) found MoL beneficial for children with ADHD but noted adherence challenges due to motivational difficulties and distractions. de Tournay‐Jetté et al. (2012) reported significant improvements in attention and memory among older adults following coronary artery bypass surgery. Saleh et al. (2015) demonstrated that preoperative cognitive training using MoL effectively reduced postoperative cognitive dysfunction in elderly patients undergoing gastrointestinal surgery.

#### Application‐based and other studies

Sandberg et al. (2021) demonstrated that a smartphone app employing MoL improved memory performance across various age groups, although older adults showed comparatively smaller improvements. Varela‐Aldás et al. (2023) also utilized a smart device application combining MoL with paired‐associate learning, reporting significant cognitive enhancements over a 2‐month period. Thana‐Udom et al. (2021) showed that memory training incorporating loci improved older adults’ self‐perceptions regarding their memory capabilities. Amico et al. (2021) found no significant memory encoding benefits from engaging in light to moderate resistance exercise. Hagström and Winman (2018) demonstrated that using MoL within a virtual environment significantly enhanced the learning of German grammatical gender. Mastrogiorgio et al. (2021) reported that both traditional and virtual loci methods were equally effective for long‐term memory recall, though virtual memoryscapes offered additional organizational benefits. Finally, Schoen (1996) introduced ‘Mnemopoly’, a mnemonic training method inspired by the Monopoly game, designed as an engaging way to teach mnemonic techniques.

## GENERAL DISCUSSION

The method of loci (MoL) has been evaluated both qualitatively, through narrative synthesis and the GRADE approach, and quantitatively, using RoBMA_PSMA_. The method consistently demonstrates its effectiveness in enhancing memory performance across various contexts and populations. Despite these well‐documented benefits, certain limitations exist, and great caution is warranted when interpreting the overall results and conclusions.

### The effectiveness of the method of loci across populations

#### Young adults

Qualitatively, multiple studies consistently demonstrate the effectiveness of the MoL among young adults, particularly in enhancing free and serial recall. Research emphasizes that the MoL effectively reduces proactive interference, maintains high recall accuracy even after prolonged retention intervals, dramatically outperforms basic learning strategies such as rehearsal, and surpasses simpler mnemonic methods like the link method, maintenance rehearsal, and specific strategies such as survival processing. Additionally, it exhibits comparable or superior effectiveness relative to more complex mnemonics, such as the Pegword method. However, a practical limitation, typically allowing only around two to‐be‐remembered items per locus, restricts its overall capacity, especially without extensive practice or training. Furthermore, the inherent complexity of the MoL may deter some users.

Quantitatively, the meta‐analysis across all young adult groups revealed strong evidence for the MoL's moderate effect size in enhancing immediate serial recall compared with rehearsal (*d* = 0.75, 95% CI [0.00, 1.33], P(M|data) = 0.93, BF = 12.26), along with very strong evidence of high heterogeneity, publication bias, and the presence of small‐study effects.

The overall GRADE rating is very low, indicating that we have very little confidence in the effect estimate and that the true effect may be substantially different. Therefore, conclusions drawn from this finding should be interpreted with great caution.

#### Older adults

Qualitatively, a smaller yet consistent body of research shows that the MoL significantly enhances both free and serial recall performance among older adults, despite age‐related declines in cognitive functions such as processing speed. When using MoL, strong instructions, extended practice, and explicit prompts significantly enhance memory performance in older adults. However, older learners tend not to spontaneously apply MoL due to its high cognitive demands, frequently opting for simpler mnemonic techniques instead.

Quantitatively, the meta‐analysis for older adults indicates a strong evidence for the MoL's large effect in immediate serial recall compared with rehearsal (*d* = 1.05, 95% CI [0.00, 1.66], [P(M|data) = 0.93, BF = 12.71]), with very strong evidence for high heterogeneity, anecdotal evidence for publication bias, and the presence of small‐study effects.

Again, the overall GRADE rating is very low, indicating that we have very little confidence in the effect estimate and that the true effect may be substantially different. Therefore, conclusions drawn from this finding should be interpreted with great caution.

#### Comparative and combined findings

Qualitatively, comparative evidence indicates that while both younger and older adults benefit from the MoL, younger adults typically achieve higher performance levels and reach peak performance more quickly, whereas older adults almost never reach the same level of performance, especially in time‐sensitive tasks that require processing speed.

Quantitatively, meta‐analytic comparisons between younger and older adults provide very strong evidence for MoL's large effect size in enhancing immediate serial recall compared with rehearsal (*d* = 2.30, 95% CI [1.37, 2.82], P(M|data) = 0.996, BF = 221.69). However, this finding is accompanied by very strong evidence of heterogeneity, anecdotal evidence of publication bias, and signs of small‐study effects, warranting cautious interpretation.

Finally, when combining both age groups into the ‘all adults’ category, the meta‐analysis based on a total of 3006 participants provides very strong evidence for MoL's large effect size in enhancing immediate serial recall compared with rehearsal (*d* = 0.88, 95% CI [0.47, 1.25], P(M|data) = 0.99, BF = 161.94). This was accompanied by very strong evidence of high heterogeneity, publication bias, and indications of small‐study effects, again warranting cautious interpretation.

### Cognitive mechanisms underlying the general effectiveness of the method of loci

According to the levels of processing framework, deeper and more meaningful encoding processes result in stronger and more enduring memory traces. The MoL leverages this framework in multiple ways by engaging several cognitive mechanisms to enhance recall. Qualitative synthesis sheds light on which of these mechanisms may play a key role, while the levels of processing framework offers a unifying perspective for understanding how the MoL improves memory performance.

For example, dual coding facilitates simultaneous visual and verbal encoding by generating vivid imagery paired with linguistic labels, thereby enhancing multimodal memory traces. The MoL inherently supports this process by transforming verbal information into vivid – and often bizarre – imagery.

Autobiographical memory, the self‐reference effect, and emotional context enhancement further strengthen encoding by linking new information to personally meaningful and familiar locations, and by encouraging users to create their own bizarre associations, which are often emotionally charged.

Context‐dependent memory may enhance retrieval success by mentally revisiting the same spatial context during recall. In the case of MoL, this refers to mentally navigating through a memory palace and its locations, thereby systematically recreating the original encoding environment, albeit only mentally.

The concept of desirable difficulty emphasizes the cognitive effort required by MoL (such as generating elaborate images), fostering deeper cognitive processing and enhanced retention.

Elaborative processing supports the creation of rich, interconnected memory representations by actively linking new information to pre‐existing knowledge frameworks, such as a familiar route and its locations within a memory palace, thereby facilitating robust retrieval cues. The long‐term working memory model may explain how familiar spatial routes serve as stable retrieval structures, supporting rapid and reliable recall even after interruptions.

Lastly, spatial binding anchors information to specific mental locations, ensuring stable, orderly memory structures easily reactivated during recall.

Despite theoretical support, the overall GRADE rating is very low, indicating that we have very little confidence in the effect estimate and that the true effect may be substantially different. Therefore, conclusions drawn from this finding should be interpreted with great caution.

### Neural correlates and neuropsychological insights

The qualitative synthesis of neuroimaging studies collectively underscores that the method of loci engages a comprehensive network of brain regions essential for spatial memory, visualization, and executive control. The hippocampus is central to spatial memory and navigation, actively encoding and retrieving sequential information and temporal order. Complementary activation of the parahippocampus supports scene construction, spatial encoding, and memory consolidation, reinforcing spatial context as a primary scaffold for memory representation and recall. The retrosplenial cortex, PCC, and precuneus further facilitate spatial navigation and episodic retrieval by anchoring memories to distinct spatial contexts, thereby enhancing recall accuracy and stability.

Moreover, executive control regions, notably the DLPFC, orchestrate mnemonic strategies by organizing and monitoring memory encoding and retrieval processes, while the inferior and middle frontal gyri link objects to their spatial loci. Visualization processes critically involve the fusiform gyrus for detailed object recognition, the lingual gyrus for vivid imagery formation, and the visual cortex for broader perceptual processing. The medial prefrontal cortex and SPL additionally contribute by directing spatial attention and consolidating memories into stable representations.

Training in the MoL induces significant neural plasticity, characterized by strengthened hippocampal–neocortical connectivity and reduced medial temporal theta power, enhancing neural efficiency and long‐term memory performance. These structural and functional adaptations, including changes in grey and white matter within the hippocampus and fusiform gyrus, underscore the method's potential to modify large‐scale neural networks positively. Together, these insights confirm the MoL's robust neurobiological basis, leveraging spatial, visual, and executive brain networks to optimize mnemonic performance.

The overall GRADE rating is low, indicating that we have little confidence in the effect estimate and that the true effect may be different.

### Miscellaneous studies

Although outside the main scope of this study, additional studies demonstrate the broader applicability and practical utility of the MoL across clinical and applied contexts. In clinical populations, MoL has shown promise in improving memory in individuals with depression, ADHD, and adults recovering from surgery. While it was not effective for individuals with schizophrenia, it enhanced episodic memory in healthy controls and supported emotional regulation in depressive disorders.

Application‐based studies further illustrate the adaptability of MoL. Smartphone apps incorporating the method improved memory across age groups, though gains were smaller in older adults. Virtual environments and games such as ‘Mnemopoly’ suggest innovative ways to implement MoL beyond traditional settings.

Together, these findings underscore the versatility of the method of loci across diverse populations and delivery formats, highlighting its relevance not only as a cognitive strategy in experimental settings but also as a tool for clinical and applied use. They enrich the broader understanding of MoL and complement the evidence‐based literature covered in the main body of the review.

### Limitations of the method of loci and its research

Despite its strengths, MoL presents practical limitations, including substantial time investment for memory palace construction, limited locus capacity (typically two items per locus), and interference issues when loci are reused. Older adults often require extended explicit training, and clinical populations may struggle with cognitive load and consistent application.

A major limitation in MoL research is the presence of methodological shortcomings. In our evaluation of 63 studies and their 85 experiments, none were assessed as having a low risk of bias. Specifically, 68.24% of the experiments were assessed as having a high risk of bias, 11.76% as having a serious risk of bias, another 11.76% as having a moderate risk of bias, and 8.24% as having some risk of bias. Common issues include variable learning times across groups, an unspecified number of locations and to‐be‐remembered items per location, a lack of detail regarding how the method was implemented, absence of a proper control or comparable group, poorly matched control conditions, ceiling effects, and the high likelihood of fatigue and practice effects. There were also problems with participant compliance in using the method, inconsistent interpretations of how to apply the method, uncontrolled confounding variables like proactive interference, discrepancies between the number of participants reported and those included in statistical analyses, high dropout rates, small sample sizes, lack of pre‐registration, inadequate randomization, and absence of blinding or assessor blinding. Additional concerns include anecdotal to very strong evidence for publication bias, the presence of small‐study effects, and very strong evidence for moderate to high heterogeneity. These factors generally result in very low to low overall GRADE ratings, with only the question focusing on the MoL in the context of neuropsychology receiving a low overall GRADE. Consequently, there is limited confidence in the estimated effects, and the actual impact of the MoL on memory performance may differ substantially from current findings.

### The final conclusion

The MoL consistently demonstrates robust effectiveness in enhancing memory performance across diverse populations and memory tasks. Supported by quantitative meta‐analyses reporting moderate to large effect sizes in immediate serial recall (*d* = 0.42–0.88) and by qualitative syntheses outlining comprehensive cognitive and neural mechanisms, the MoL emerges as a powerful and versatile mnemonic strategy.

Its effectiveness arises from the integration of multimodal cognitive processes, including dual coding, self‐reference, autobiographical memory, context‐dependent memory, vividness of visual imagery, long‐term working memory, spatial binding, and elaborative processing, all of which are unified under the levels of processing framework.

These processes engage extensive neural networks, primarily involving brain regions essential for spatial memory, navigation, visualization, and executive control, and can induce significant neural plasticity.

To directly address the core questions. First, the MoL is a mnemonic that significantly enhances memory performance in adults, particularly in immediate serial recall tasks when compared with rehearsal. Second, it is grounded in deep levels of encoding, drawing upon a range of cognitive mechanisms that facilitate durable memory traces. Third, it activates key brain regions associated with spatial memory, navigation, and visualization.

However, given the substantial methodological concerns identified in the existing literature, these findings should be interpreted with great caution. More rigorous, well‐designed research is essential to clarify the full scope and boundaries of the MoL's practical applications.

### Study's limitations

The study has several limitations. First, the study does not encompass the full scope of literature on the method of loci. Numerous historical texts dating back to ancient times could provide additional general insights. Many conference papers present innovative ideas that might shed more light on the method's cognitive mechanisms and creative applications. Several master's theses and dissertations offer additional relevant knowledge. However, expanding the scope to include all these sources would have been overwhelming, and the decision was made to focus solely on the evidence base.

Second, despite the limited number of included studies, using the RoBMA_PSMA_ enabled us to quantify evidence of the effect, heterogeneity, and publication bias. Although publication bias cannot be ruled out, as evidence is very strong, using RoBMA_PSMA_ allowed us to provide reasonably accurate effect size estimates that account for the possibility of publication bias and heterogeneity. This approach helped us avoid confirmation bias and all‐or‐nothing decisions regarding these factors (Bartoš et al., 2022). However, RoBMA_PSMA_ does not account for the dependency of effect sizes. We addressed this limitation by averaging dependent effects, though this method artificially reduces the variance between effect sizes. This meta‐analysis may have led to underestimating the effect size estimates and the Bayesian factors supporting the estimated effects. An alternative approach for handling dependent effect sizes could involve correlated and hierarchial effects models with robust variance estimation (CHE‐RVE: Harrer et al., 2021). However, this approach requires additional information about the correlation between dependent effects within studies, which none reported. As a result, this method relies on estimation, which could be considered data manipulation, potentially leading to overestimation. Given this, we opted to report more conservative results despite the possibility of underestimation of effect size estimates and Bayesian factors.

Third, conducting the study alone without other assessors to evaluate the risk of bias could introduce bias into the evaluation process.

Fourth, in certain studies (De Beni et al., 1997; Moè & De Beni, 2005), only effect sizes from oral presentations were used, as the written formats intentionally interfered with the method of loci, rendering them unusable for meta‐analysis and limiting the results included.

Lastly, incomplete reporting of necessary data across studies posed significant limitations. Some studies lacked demographic or inferential information, requiring post‐hoc calculations of effect sizes and confidence intervals. Many did not provide the necessary statistics, making them unsuitable for quantitative analysis. Variations in effect size derivation and the need to select and transform multiple effect sizes per study potentially reduced measurement precision and limited the number of studies included.

## AUTHOR CONTRIBUTIONS

**Jan Ondřej:** Conceptualization; investigation; writing – original draft; validation; methodology; visualization; writing – review and editing; software; formal analysis; project administration; funding acquisition; data curation; resources.

## FUNDING INFORMATION

The study was funded by Masaryk University, Faculty of Arts, Department of Psychology, Czech Republic, through the specific research project [MUNI/A/1519/2023].

## CONFLICT OF INTEREST STATEMENT

We do not have any known competing interests to disclose.

## Supporting information


Appendix S1

## Data Availability

The data are available on the Open Science Framework at https://doi.org/10.17605/OSF.IO/SK2RV.
