# Supplementary material for: The method of loci in the context of psychological research: A systematic review and meta‐analysis
Source: Br J Psychol. 2025 Jun 3;116(4):930–86. doi: 10.1111/bjop.12799 (PMC12514325; doi:10.1111/bjop.12799)
Supplement: Supplementary file 1 — Appendix S1 [file BJOP-116-930-s001.docx]

**the Method of Loci in the Context of Psychological Research: A Systematic Review and Meta-Analysis**

Supplementary materials

### Additional Meta-Analyses data

##### The MoL’s effectiveness in young adults

A lenient meta-analysis including all serial recall effect size formulas

A RoBMA_PSMA_ of 1267 participants (mean age = 23.09, aged 17–44) evaluated serial immediate recall compared to free learning or rehearsal. Strong evidence was found for a moderate effect (d = 0.56, 95% CI [0.00, 0.91], [P(M|data) = 0.96, BF = 21.57]). There was extremely strong evidence for high heterogeneity (τ = 0.51, [P(M|data) = 1, BF = 5.98 × 10⁶]) and publication bias ([P(M|data) = 1, BF = 2.94 × 10⁷]). PET-PEESE analyses indicated small study effects, with PET showing a negligible effect (d = 0.04) and PEESE showing an inflated effect size (d = 8.95, 95% CI [6.90, 11.12]). In conclusion, strong evidence supports a moderate effect, extremely strong evidence for heterogeneity, publication bias and potential small study bias advising caution in interpreting free recall findings. See Table 1 for effect sizes and studies used and Figure 1 for forest plots.

| **Table 1**  *Studies with calculable effect sizes when comparing the MoL with rehearsal and free learning strategy in serial immediate recall* | |
| --- | --- |
| **Citation** | **Serial recall (d, 95 % CI)** |
| Roediger (1980)^6^ | MoL vs Rehearsal: *d* = 2.18, [1.55, 2.82] |
| Weinsein et al. (1981) Experiment 1^1^ | Elaborated instructions MoL vs rehearsal: *d* = 0.86, [0.21, 1.50]  Training MoL vs rehearsal: *d* = 2.10, [1.33, 2.88]  Elaborated training MoL vs rehearsal: *d* = 1.90, [1.16, 2.65]  Standard MoL vs rehearsal: *d* = 0.16, [-0.47, 0.78]  Weighted Mean Effect Size: *d* = 1.11, [0.77, 1.46] |
| De Beni & Cornoldi (1985) Experiment 2^3^ | *d =* 1.08, [0.47, 1.68] |
| De Beni & Cornoldi (1988)^1^ | Concrete words: List 1: *d =* 0.28, [-0.11, 0.67], List 2: *d =* 0.67, [0.27, 1.06], List 3: *d =* 0.41, [0.02, 0.80]  Abstract words: List 1: *d* = 0.54, [0.15, 0.93], List 2: *d =* 1.20, [0.78, 1.62], List 3: *d =* 0.78, [0.38, 1.17]  Weighted Mean Effect Size: *d* = 0.63, [0.47, 0.79] |
| Cornoldi & De Beni (1991) Experiment 1^1^ | Oral presentation: *d =* 0.98*, [0.10, 1.84]  Written presentation: *d =* 0.60, [-0.32, 1.52] Weighted Mean Effect Size: *d* = 0.80, [0.17, 1.43] |
| Cornoldi & De Beni (1997) Experiment 1^4^ | MoL vs Rehearsal: *d =* 1.44, [1.05, 1.84] |
| Cornoldi & De Beni (1997) Experiment 2^1^ | Written presentation: *d =* -0.45, [-1.39, 0.49]  Oral presentation: *d =* 0.80*, [-0.17, 1.76]  Weighted Mean Effect Size: *d* = 0.16, [-0.51, 0.83] |
| Cornoldi & De Beni (1997) Experiment 3^1^ | Written presentation: *d =* -1.46, [-2.57, -0.37]  Oral presentation: *d =* 4.25*, [2.48, 6.03]  Weighted Mean Effect Size: *d* = 0.12, [-0.81, 1.06] |
| Moe & De Beni (2005)^1?^ | *Oral presentation:* Descriptive passage: *d* = 0.82, [-0.09, 1.73] for Subject generated vs rehearsal; *d* = 0.36, [-0.53, 1.24] for Experimentator genereted vs rehearsal; *d* = 0.55, [-0.34, 1.44] for Subject vs Experimentator generated. Narrative passage: d = 1.42, [0.44, 2.40] for Subject generated vs rehearsal; *d* = 1.36, [0.39, 2.34] for Experimentator genereted vs rehearsal; *d* = 0.16, [-0.72, 1.04] for Subject vs Experimentator generated. Expository passage*: d* = 2.04, [0.96, 3.12] for Subject generated vs rehearsal; *d* = 1.32, [0.35, 2.28] for Experimentator genereted vs rehearsal; *d* = 0.64, [-0.26, 1.54] for Subject vs Experimentator generated  Weighted Mean Effect Size: *d* = 0.93, [0.70, 1.16]  *Written presentation:*  Descriptive passage: *d* = -1.13, [-2.07, -0.18] for Subject generated vs rehearsal; *d* = -0.65, [-1.55, 0.24] for Experimentator generated vs rehearsal; *d* = -0.49, [-1.38, 0.40] for Subject vs Experimentator generated. Narrative passage: *d* = -0.50, [-1.39, 0.39] for Subject generated vs rehearsal; *d* = 0.12, [-0.76, 1.00] for Experimentator generated vs rehearsal; *d* = -0.70, [-1.61, 0.20] for Subject vs Experimentator generated. Expository passage: *d* = -1.94, [-3.00, -0.87] for Subject generated vs rehearsal; *d* = -1.40, [-2.37, -0.42] for Experimentator generated vs rehearsal; *d* = -0.45, [-1.34, 0.44] for Subject vs Experimentator generated.  Weighted Mean Effect Size: *d* = -0.76, [-0.99, -0.54]  Written and Oral presentation Weighted Mean Effect Size: *d* = 0.13, [-0.14, 0.40]  *Comparsion between passages:*  Descriptive passage: *d*= 1.13, [0.19, 2.10] for Subject generated Oral vs Written; *d* = 0.23, [-0.65, 1.11] for Experimentator generated Oral vs Written. Narrative passage: *d* = 0.84, [-0.07, 1.76] for Subject generated Oral vs Written; *d* = 0.14, [-0.74, 1.02] for Experimentator generated Oral vs Written. Expository passage: *d* = 2.33, [1.20, 3.47] for Subject generated Oral vs Written; *d*= 1.01, [0.08, 1.94] for Experimentator generated Oral vs Written.  *All passages together comparsion of written vs oral presentation:*  Subject-generated: *d* = -1.67, [-2.26, -1.08]; Experimenter-generated: *d* = -0.66, [-1.18, -0.14]  *All passages together comparsion oral:*  Subject-generated vs rehearsal: *d* = 2.06, [1.44, 2.67]; Experimenter-generated vs rehearsal: *d* = 1.56, [0.99, 2.14]; Subject vs Experimentator generated: *d* = 0.43, [-0.08, 0.94]  *All passages together comparsion written:*  Subject-generated vs rehearsal: *d* = -1.23, [-1.79, -0.68]; Experimenter-generated vs rehearsal: *d* = -0.68, [-1.20, -0.16]; Subject vs Experimentator generated: *d* = -0.74, [-1.26, -0.22] |
| Massen & Vaterrodt-Plünnecke (2006) Experiment 1^1^ | Loci Similar vs Rehearsal Similar: *d* = 0.43, [-0.18, 1.05] for List 1; *d* = 0.23, [-0.38, 0.84] for List 2; *d* = 0.32, [-0.29, 0.93] for List 3  Loci Dissimilar vs Rehearsal Dissimilar: *d* = 0.04, [-0.57, 0.65] for List 1; *d* = 0.41, [-0.20, 1.03] for List 2; *d* = 1.07, [0.42, 1.72] for List 3  Weighted Mean Effect Size: *d* = 0.40, [0.15, 0.66]  Loci Similar: *d* = -0.41, 95% CI [-0.88, 0.05] indicating a moderate decline in performance  Loci Dissimilar: *d* = 0.91, 95% CI [0.38, 1.44] indicating a significant improvement in performance. |
| Massen & Vaterrodt-Plünnecke (2006) Experiment 2^1^ | Loci Similar vs Rehearsal Similar: *d* = 0.77, [0.26, 1.27] for List 1; *d* = 0.46, [0.00, 0.93] for List 2; *d* = 0.29, [-0.16, 0.74] for List 3  Loci Dissimilar vs Rehearsal Dissimilar: *d* = 0.21, [-0.24, 0.66] for List 1; *d* = 0.53, [0.06, 1.01] for List 2; *d* = 0.35, [-0.10, 0.81] for List 3  Weighted Mean Effect Size: *d* = 0.42, [0.23, 0.61]  Loci Similar: *d* = -0.11, 95% CI [-0.56, 0.33] indicating a slight decline in performance  Loci Dissimilar: *d* = 0.24, 95% CI [-0.21, 0.69] indicating a slight improvement in performance |
| Legge et al. (2012)^5^ | All participants (group: η_p_^2^ = 0.002, p > 0.1)  Compliant only (group: η_p_^2^ = 0.14): cMOL vs. CON: *d* = 0.47, [0.06, 0.88]; vMOL vs. CON: *d* = 0.33 [-0.07, 0.73]; cMOL vs. vMOL: *d* = 0.13 [-0.27, 0.53]  Weighted Mean Effect Size: *d* = 0.40, [0.11, 0.68] |
| Mallow et al. (2015)^5^ | MoL vs Control in digit recall: *d* > 4, [2.72, 5.28] |
| McCabe (2015)^1^ | *d* = 0.81, [0.54, 1.08] |
| Saraiva et al. (2016)^1^ | MoL vs Free strategy (η^2^ = 0.73): d = 9.39, [6.82, 11.96] |
| Kroneisen & Makerud (2017) Experiment 1^NCE^ | (MoL vs Survival vs Imagery: η_p_^2^ = 0.37) |
| Kroneisen & Makerud (2017) Experiment 2^NCE^ | (MoL vs Survival vs Control: η_p_^2^ = 0.28) |
| Liu et al. (2022)^5^ | MoL vs baseline: *d* = 1.56, [0.98, 2.14]; MoL vs 1st practice: *d* = 1.14, [0.64, 1.64]; MoL vs last practice: *d* = 0.75, [0.31, 1.19]  Weighted Mean Effect Size: *d* = 1.08, [0.79, 1.36] |
| Notes:  1 = Means, Standard Deviations, and Sample Sizes  2 = p-value from a Student's t-test with Unequal Sample Sizes  3 = Student's t-test and Total Sample Size  4 = Oneway F-test with Two Groups and Equal Sample Sizes  5 = Effect sizes reported, 95 % CI calculated using formula CI = d±Z*SE_d_  6 = Oneway ANOVA with three or More Groups  NCE = No calculable effect sizes for a specific method  * = Only these effect sizes were used fo the primary meta-analysis because written presentations were intentionally designed to create interference between the use of the Method of Loci and the act of reading the presented text, which, in my opinion, makes the resulting effects unreliable and unusable for meaningful analysis  Underscored studies = Studies used for the main Meta-analysis  Underscored effect sizes = Effect sizes used for calculating weighted means | |


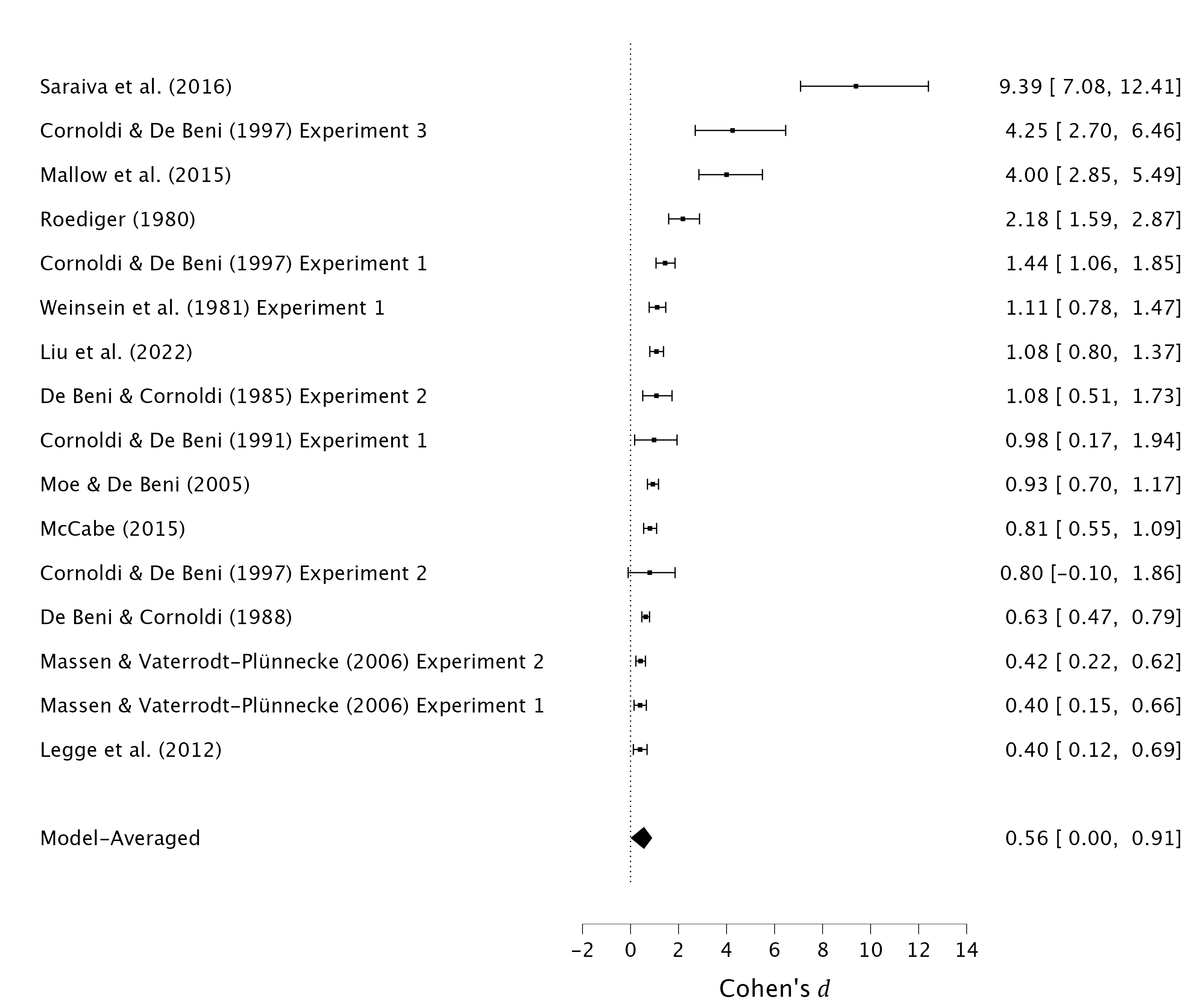


**Figure 1**

Forrest plot for all serial recall effect sizes in young adults.

A lenient meta-analysis, including all free recall effect sizes

A RoBMA_PSMA_ of 416 participants (mean age = 19.19, aged 17–27) assessed free immediate recall compared to free learning or rehearsal. Anecdotal evidence was found for a negligible effect (d = 0.19, 95% CI [−0.18, 0.78], [P(M|data) = 0.51, BF = 1.06]). Anecdotal evidence was also found for high heterogeneity (τ = 0.27, [P(M|data) = 0.73, BF = 2.67]) and moderate publication bias ([P(M|data) = 0.83, BF = 5.01]). PET-PEESE analyses suggested small study effects, with PET showing a moderate effect (d = 0.79, 95% CI [0.00, 4.00]) and PEESE an inflated effect (d = 3.41, 95% CI [0.00, 18.48]). In conclusion, anecdotal evidence supports a negligible effect, high heterogeneity, and moderate evidence for publication bias. Table 2 summarises the effect sizes and formulas used.

| **Table 2**  *Studies with calculable effect sizes when comparing the MoL with rehearsal and free learning strategies in free immediate recall* | |
| --- | --- |
| **Citation** | **Free recall (d, 95 % CI)** |
| Roediger (1980)^6^ | MoL vs Rehearsal: *d* = 1.37, [0.81, 1.93] |
| Legge et al. (2012)^5^ | All participants (group: η_p_^2^ = 0.06): cMOL vs. CON: *d* = 0.24, [-0.17, 0.65]; vMOL vs. CON: *d* = 0.27, [-0.13, 0.67]; cMOL vs. vMOL: *d* = 0.03 is [-0.37, 0.43]  Compliant only (group: η_p_^2^ = 0.24): cMOL vs. CON: *d* = 0.62, [0.21, 1.03]; vMOL vs. CON: *d* = 0.49, [0.08, 0.90]; cMOL vs. vMOL: *d* = 0.12, [-0.28, 0.52]  Weighted Mean Effect Size: *d* = 0.40, [0.20, 0.61] |
| Bass & Oswald (2014)^1^ | MoL vs No instructions in List 1: *d* = 0.47, [0.06, 0.88]; MoL vs No instructions in List 5: *d* = 0.69, [0.27, 1.11]; No instructions List 1 vs List 5: *d* = -1.66, [-2.12, -1.19]; MoL List 1 vs List 5: *d* = -1.08. [-1.51, -0.64]  All lists MoL vs No instructions: d = 0.48, [0.07, 0.89] |
| McCabe (2015)^1^ | d = 0.36, [-0.06, 0.78] |
| Bouffard et al. (2017) Experiment 1^NCE^ | MoL vs Free strategy vs Autobiographical: Learning: η_p_^2^ = 0.54; “Delayed” free recall: η_p_^2^ = 0.41; Final free recall: η_p_^2^ = 0.57 |
| Bouffard et al. (2017) Experiment 2^NCE^ | MoL vs Free strategy vs Procedural: Learning: η_p_^2^ = 0.53; “Delayed” free recall: η_p_^2^ = 0.40; Final free recall: η_p_^2^ = 0.46 |
| Dresler et al. (2017)^NCE^ | (MoL vs Control: η^2^ = 0.47) |
| Notes:  1 = Means, Standard Deviations, and Sample Sizes  5 = Effect sizes reported, 95 % CI calculated using formula CI = d±Z*SE_d_  NCE = No calculable effect sizes for a specific method  Underscored studies = Studies used for the main Meta-analysis  Underscored effect sizes = Used effect sizes, or used effect size for calculating weighted means | |


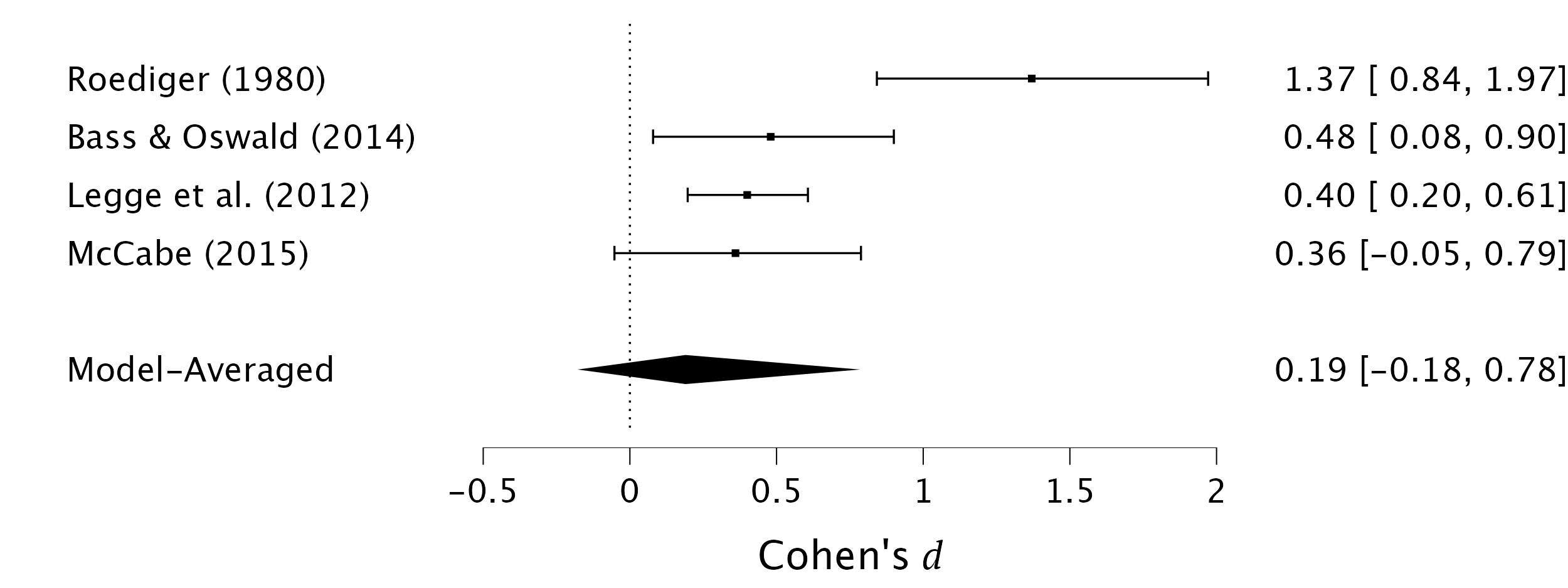


**Figure 1**

Forrest plot for all free recall effect sizes in young adults.

A lenient meta-analysis for all serial and free recall effect sizes

A RoBMA_PSMA_ of 1361 participants (mean age = 22.66, aged 17–44) examined serial and free immediate recall compared to free learning or rehearsal. Strong evidence was found for a small effect (d = 0.49, 95% CI [0.00, 0.79], [P(M|data) = 0.953, BF = 20.1]), with extremely strong evidence for high heterogeneity (τ = 0.45, [P(M|data) = 1, BF = 3.26 × 10⁶]) and publication bias ([P(M|data) = 1, BF = 2.29 × 10⁸]). PET-PEESE analyses indicated small study effects, with PET showing a negligible effect (d = 0.01) and PEESE an inflated effect (d = 9.05). In conclusion, strong evidence supports a small effect, but extreme evidence for high heterogeneity and publication bias warrants cautious interpretation. See Tables 1 and 2 for the effect sizes used. See Figure 3 for forest plots.


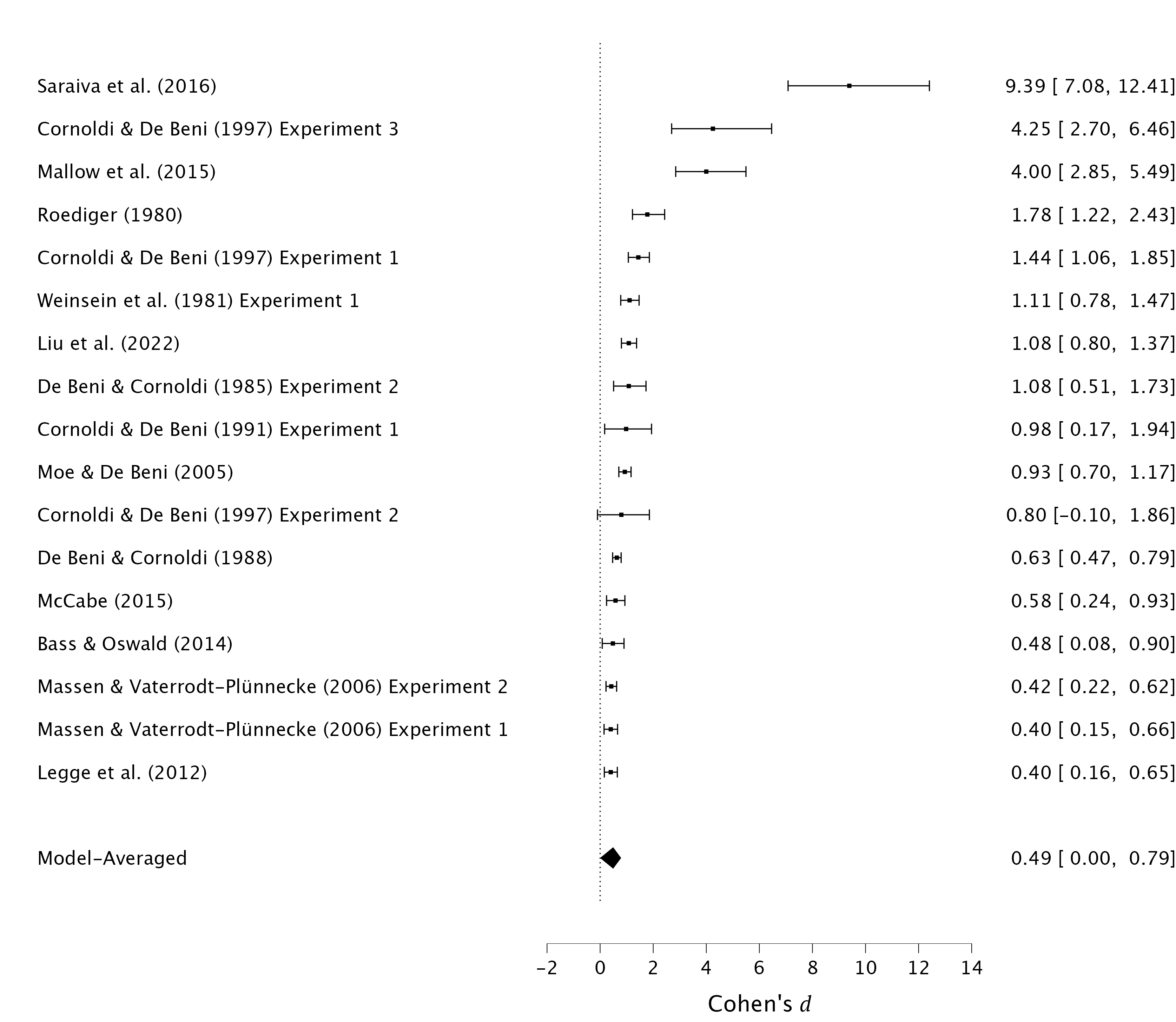


**Figure 3**

Forrest plot for all serial and free recall effect sizes in young adults.

##### The MoL compared to the free learning strategy in short, medium and long retention intervals

The evidence for the effectiveness of the MoL compared to rehearsal and free learning strategy in short, medium and long retention intervals is based on 406 participants, with an approximate mean age of 23.66, aged 20–36.6. Compared to rehearsal and the free learning strategy, the MoL’s effect sizes are positive, ranging from moderate to large for short-delayed 24-hour recall. In moderate-delayed one-week serial and free recall compared to free learning strategy or rehearsal, the MoL’s effect size ranges from small negative to large positive. The MoL effect size is large in long-delayed five-week free and serial recall compared to a free learning strategy (only Groninger, 1971). Table 3 summarises the effect sizes and formulas used.

| **Table 3**  *Studies with calculable effect sizes when comparing the MoL with rehearsal and free learning strategies in delayed serial or free recall* | | | | | | |
| --- | --- | --- | --- | --- | --- | --- |
| **Citation** | **24 hours (d, 95% CI)** | | **1 week (d, 95% CI)** | | **5 weeks (d, 95% CI)** | |
|  | **Free learning strategy serial recall** | **Free learning strategy free recall** | **Free learning strategy serial recall** | **Free learning strategy free recall** | **Free learning strategy serial recall** | **Free learning strategy free recall** |
| Groniger (1971)^2^ |  |  | *d* = 1.05 [0.43, 1.68] | *d* = 0.88  [0.27, 1.5] | *d* = 1.18  [0.48, 1.89] | *d* = 1.18  [0.48, 1.89] |
| Roediger (1980)^6^ | MoL vs Rehearsal: *d* = 2.18, [1.54, 2.81] | MoL vs Rehearsal: *d* = 2.11, [1.49, 2.74] |  |  |  |  |
| Cornoldi & De Beni (1997) Experiment 3^1^ |  |  | MoL vs Rehearsal written condition: *d* = -0.31, [-1.30, 0.67]; MoL vs Rehearsal oral condition: *d* = 3.73, [2.11, 5.35]; MoL oral vs written: *d* = 2.87, [1.48, 4.27] |  |  |  |
| Qureshi et al. (2014)^1^ |  | *d* = 0.73, [0.25, 1.22] |  |  |  |  |
| Dresler et al. (2017)^NCE^ |  | (MoL vs Control: η² = 0.58) |  |  | (MoL vs Control: η² = 0.39) |  |
| Notes:  1 = Means, Standard Deviations, and Sample Sizes  2 = p-value from a Student's t-test with Unequal Sample Sizes  3 = Student's t-test and Total Sample Size  6 = Oneway ANOVA with three or More Groups  NCE = No calculable effect sizes for a specific method | | | | | | |

##### The MoL’s effectiveness compared to mnemonic strategies in immediate recall

A lenient meta-analysis for all serial recall effect size formulas when comparing to other mnemonics

A RoBMA_PSMA_ of 683 participants (mean age = 24.94, aged 18–44) evaluated the MoL in serial immediate recall compared to mnemonics. No evidence of an effect was found (d = 0.17, 95% CI [−0.46, 1.22], [P(M|data) = 0.43, BF = 0.76]). Extremely strong evidence was found for high heterogeneity (τ = 0.76, [P(M|data) = 1, BF = 6744.53]) and moderate publication bias ([P(M|data) = 0.92, BF = 11.10]). PET-PEESE analyses suggested small study effects, with PET estimating a moderate effect (d = 1.14, 95% CI [0.00, 6.87]) and PEESE an inflated effect (d = 7.94, 95% CI [0.00, 21.697]). In conclusion, no effect was found, with strong evidence for high heterogeneity, moderate publication bias, and potential inflation due to small study effects. Table 4 summarises the effect sizes and formulas used. See Figure 4 for forest plots.

| **Table 4**  *Studies with calculable effect sizes when comparing the MoL with mnemonics in serial immediate recall* | |
| --- | --- |
| **Citation** | **Serial recall (d, 95% CI)** |
| Roediger (1980)^6^ | MoL vs Imagery: *d* = 2.47, [1.76, 3.18]  MoL vs Link: *d* = 1.12, [0.58, 1.67]  MoL vs Pegwords *d* = 0.31, [-0.19, 0.81]; Weighted Mean Effect Size: *d* = 1.06, [0.73, 1.39] |
| De Beni & Cornoldi (1985) Experiment 1^4^ | MoL vs Progresive Elaboration method: *d* = 1.55, [0.71, 2.40] |
| Massen & Vaterrodt-Plünnecke (2006) Experiment 1^1^ | MoL Similar vs Link Similar: *d* = 0.19, [-0.42, 0.80] for List 1, *d* = -0.03, 95% CI [-0.63, 0.58] for List 2, and *d* = -0.02, 95% CI [-0.63, 0.58] for List 3  MoL Dissimilar vs Link Dissimilar: *d* = 0.25, [-0.37, 0.86] for List 1, *d* = 0.47, [-0.15, 1.08] for List 2, *d* = 0.80, [0.16, 1.43] for List 3  Weighted Mean Effect Size: *d* = 0.27, [0.02, 0.52]  Loci Similar: *d* = -0.41, [-0.88, 0.05], indicating a moderate decline in performance; Loci Dissimilar: *d* = 0.91, 95% CI [0.38, 1.44], indicating a significant improvement in performance  (Link Similar: *d* = -0.20, [-0.65, 0.25], indicating a slight decline in performance; Link Dissimilar: *d* = 0.00, [-0.44, 0.44], indicating no change in performance) |
| Massen & Vaterrodt-Plünnecke (2006) Experiment 2^1^ | Loci Similar vs Link Similar: List 1: *d* = 0.60, [0.12, 1.08] for List 1; *d* = 0.69, [0.20, 1.19] for List 2; *d* = 0.36, [-0.10, 0.82] for List 3  Loci Dissimilar vs Link Dissimilar: *d* = 0.59, [0.11, 1.07] for List 1, d = 0.77, [0.26, 1.27] for List 2, d = 0.26, [-0.19, 0.71] for List 3  Weighted Mean Effect Size: d = 0.53, [0.34, 0.73]  Loci Similar: *d* = -0.11, [-0.56, 0.33] indicating a slight decline in performance; Loci Dissimilar: *d* = 0.24, [-0.21, 0.69] indicating a slight improvement in performance.  (Rehearsal Dissimilar: *d* = 0.17, [-0.27, 0.62] indicating a slight improvement in performance; Link Similar: *d* = 0.11, [-0.34, 0.55] indicating a minimal improvement in performance; Link Dissimilar: *d* = 0.72, [0.22, 1.21] indicating a significant improvement in performance) |
| Fellner et al. (2016)^3^ | EEG group MoL vs Pegs: *d* = 4.46, [2.86, 6.06]  fMRI group MoL vs Pegs: *d* = 2.61, [1.5 , 3.7]  Weighted Mean Effect Size: d = 3.20, [2.30, 4.11] |
| Kroneisen & Makerud (2017) Experiment 1^NCE^ | (MoL vs Survival vs Imagery: η_p_^2^ = 0.37) |
| Kroneisen & Makerud (2017) Experiment 2^NCE^ | (MoL vs Survival vs Control: η_p_^2^ = 0.28) |
| Kluger et al. (2022)^5^ | MoL vs Activity: d = 0.26, [-0.14, 0.66] |
| Notes:  1 = Means, Standard Deviations, and Sample Sizes  3 = Student's t-test and Total Sample Size  4 = Oneway F-test with Two Groups and Equal Sample Sizes  5 = Effect sizes reported, 95 % CI calculated using formula CI = d±Z*SE_d_  6 = Oneway ANOVA with three or More Groups  NCE = No calculable effect sizes for a specific method  Underscored effect sizes = Effect sizes used for calculating weighted means | |


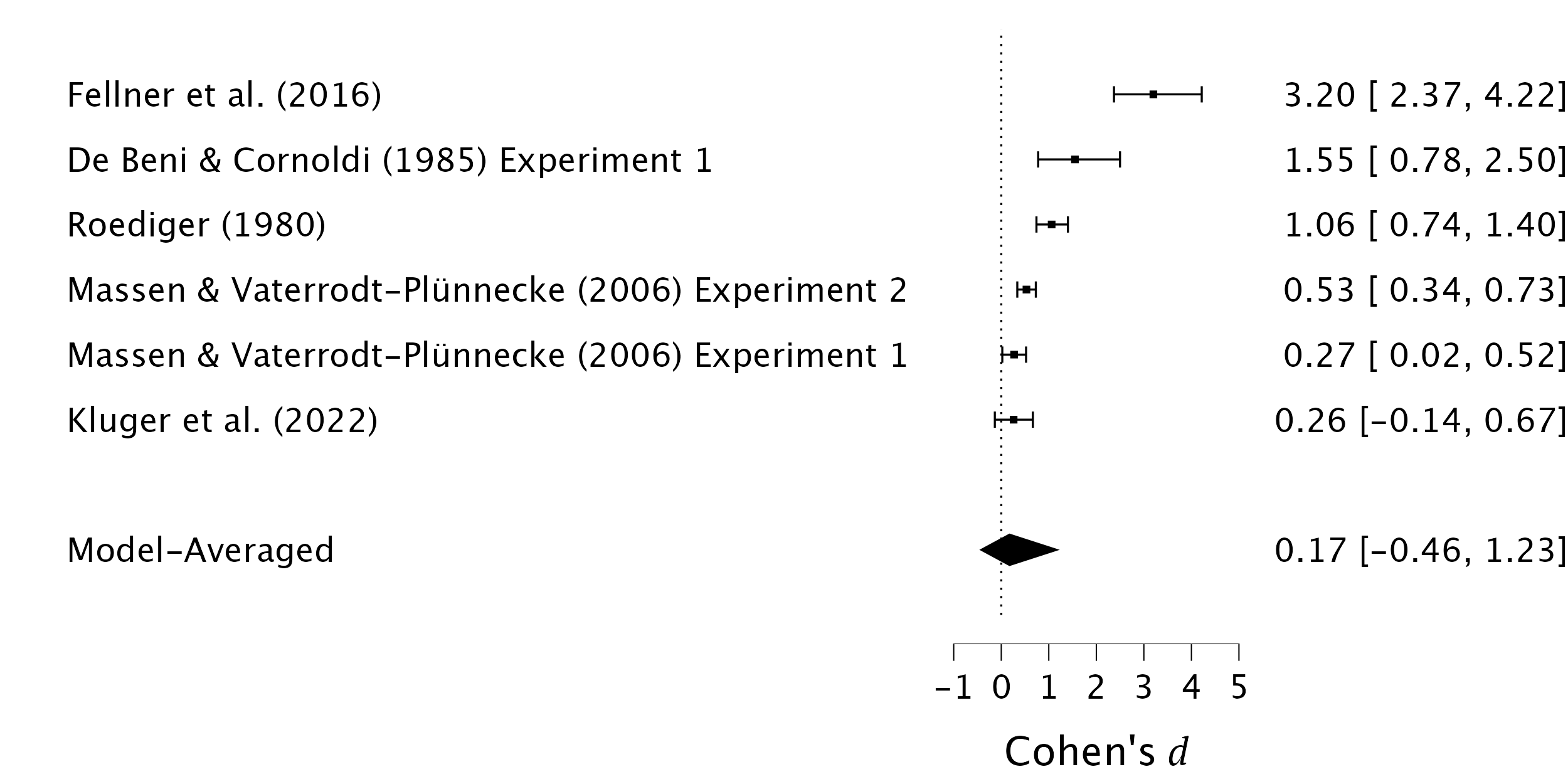


**Figure 2**

Forrest plot for all serial recall effect sizes in young adults compared to other mnemonics.

For immediate free recall (only Roediger, 1980), all calculable effect sizes are positive compared to memory strategies except one. Positive effect sizes generally range from small to very large. The negative effect size is negligible (d = -0.11). Table 5 summarises the effect sizes and formulas used.

| **Table 5**  *Studies with calculable effect sizes when comparing the MoL with mnemonics in free immediate recall* | |
| --- | --- |
| **Citation** | **Free recall (d, 95% CI)** |
| Roediger (1980)^6^ | MoL vs Imagery: *d* = 0.77, [0.21, 1.33]; MoL vs Link: *d* = -0.11, [-0.61, 0.40]; MoL vs Pegwords *d* = 0.39, [-0.12, 0.89]; Weighted Mean Effect Size: d = 0.32, [0.02, 0.62]. |
| Bouffard et al. (2017) Experiment 1^NCE^ | MoL vs Free strategy vs Autobiographical:  Learning: η_p_^2^ = 0.54; “Delayed” free recall: η_p_^2^ = 0.41; Final free recall: η_p_^2^ = 0.57 |
| Bouffard et al. (2017) Experiment 2^NCE^ | MoL vs Free strategy vs Procedural:  Learning: η_p_^2^ = 0.53; “Delayed” free recall: η_p_^2^ = 0.40; Final free recall: η_p_^2^ = 0.46 |
| Notes:  6 = Oneway ANOVA with three or More Groups  NCE = No calculable effect sizes for a specific method  Underscored effect sizes = Effect sizes used for calculating weighted means | |

##### The MoL’s effectiveness compared to mnemonic strategies in the short and medium retention interval

The evidence for the effectiveness of the MoL compared to rehearsal and free learning strategy in short and medium retention intervals is based on 150 with an unknown specific mean age or age range. Only two studies showed calculable effect sizes when comparing the MoL with other mnemonic strategies, and all effect sizes were positive. For short 24-hour retention intervals in strict scoring, the MoL’s effect sizes range from small to large. In the case of lenient scoring, all effect sizes are positive except one. The positive effect sizes range from moderate to large. The negative effect size is small (*d* = -0,30; Roediger, 1980). In medium 1-week retention intervals, the MoL has a large effect. Table 6 summarises the effect sizes and used formulas.

| **Table 6**  *Studies with calculable effect sizes when comparing the MoL with mnemonics in delayed serial and free recall.* | | | |
| --- | --- | --- | --- |
| **Citation** | **24 hours** | | **1 week** |
|  | **Mnemonic strategy serial recall** | **Mnemonic strategy free recall** | **Mnemonic strategy free recall** |
| Roediger (1980)^6^ | MoL vs Imagery: *d* = 2.33, [1.63, 3.02]  MoL vs Link: *d* = 0.39, [-0.12, 0.90]  MoL vs Pegwords *d* = 0.44, [-0.07, 0.94] | MoL vs Imagery: *d* = 1.87, [1.23, 2.50]  MoL vs Link: *d* = -0.30, [-0.80, 0.21]  MoL vs Pegwords *d* = 0.69, [0.18, 1.20] |  |
| De Beni & Cornoldi (1985) Experiment 2^4^ |  |  | MoL vs Progresive elaboration method: *d* = 1.94, [1.31, 2.58] |
| Notes:  4 = Oneway F-test with Two Groups and Equal Sample Sizes  6 = Oneway ANOVA with three or More Groups | | | |

##### The summary of meta-analytic results

Across five RoBMA_PSMA_ (see Supplementary Materials for the additional four), findings show varying levels of evidence for effect sizes, heterogeneity, and publication bias. Three analyses provide moderate to strong evidence for small to moderate effect sizes (*d* = 0.42–0.55), while two indicate no significant effect (*d* = 0.16–0.19).

The primary meta-analysis supports small effect sizes with moderate strength. All analyses consistently found strong evidence for high heterogeneity (*τ* = 0.27–0.76) and publication bias. PET-PEESE analyses showed that small study effects likely inflated effect sizes, calling for caution in interpreting these results. While there is evidence of an effect, it is critical to account for small study bias, high heterogeneity, and strong evidence of publication bias. Table 7 summarises the meta-analytic results, but only meta-analysis 1 is the most relevant due to consistent effect size calculations, as the others use different formulas for calculating Cohen’s *d* but still bring new information to the overall analysis.

| **Table 7**  *Results from multiple meta-analyses* | | | | | |
| --- | --- | --- | --- | --- | --- |
| **Meta-analysis** | **Effect sizes used** | **Number of studies** | **Effect size, 95 % CI, BF** | **Heterogenity, 95 % CI, BF** | **Publication bias BF** |
| 1 | **Serial immediate recall when compared to free learning strategy or rehearsal^1^** | 13 | ***d* = 0.42, [0.00, 0.80], 6.23** | *τ* = 0.46, [0.18, 0.90], 12196.17 | 6.41×10^6^ |
| 2 | Serial immediate recall when compared to free learning strategy or rehearsal^2^ | 15 | *d* = 0.56, [0.000, 0.91], 21.57 | *τ* = 0.51, [0.27, 0.89], 5.98×10^6^ | 2.94×10^7^ |
| 3 | Free immediate recall when compared to free learning strategy or rehearsal^2^ | 4 | *d* = 0.19, [-0.150, 0.794], 1.06 | *τ* = 0.27, [0.00, 1.03], 2.67 | 5.013 |
| 4 | Serial and free immediate recall when compared to free learning strategy or rehearsal^2^ | 17 | *d* = 0.49, [0.00, 0.79], 20.24 | τ = 0.45, [0.23, 0.79], 3.28×10^6^ | 2.32×10^8^ |
| 5 | Effectiveness in serial immediate recall compared to other mnemonics^2^ | 6 | *d* = 0.16, [-0.51, 1.20], 0.75 | *τ* = 0.76, [0.25, 1.90], 6710.92 | 11.12 |
| Notes:  1 = Effect size formula based only on Means, SD or SE and N  2 = All effect size formulas | | | | | |

### The MOL in the context of neuropsychology

See Table 7 for a detailed description before synthesising the results of identified brain regions using the MoL and their activation and decreased activation.

| **Table 7**  *The MoL in the context of neuropsychology: Brain ragions identified as important* | |
| --- | --- |
| **Citations** | **Important findings** |
| Maguire et al. (2003) | *Activation experimental > control group:*  ↑R posterior hippocampus  ↑L retrosplenial cortex  ↑R retrosplenial cortex  ↑R cerebellum  ↑L medial superior parietal cortex  ↑R cingulate cortex  ↑L fusiform cortex  ↑L posterior inferior frontal sulcus  ↑vicinity of R pallidum  ↑vicinity of L pallidum  *Only active:*  L medial superior parietal gyrus  L retrosplenial cortex  R retrosplenial cortex |
| Nyberg et al. (2003) | *Using MoL:*  ↑L occipito-parietal cortex  ↑dorsal BA 19 (peak)  ↑L dorsolateral prefrontal cortex  ↑L retrosplenial cortex *Encoding locations:*  ↑R parietal cortex  ↑L parietal cortex  ↑medial parietal cortex  ↑R dorsal frontal cortex |
| Kondo et al. (2005) | *Encoding to-be-remembered items:*  ↑L middle frontal gyrus (BA 6/8), z = 3.50  ↑L fusiform gyrus (BA 37), z = 4.22 ↑L lingual gyrus, z = 4.97  ↑R cingulate gyrus, z = 4.97  ↑R inferior frontal gyrus (BA 45), z = 3.42  ↑R middle frontal gyrus (BA 8), z = 3.73  ↑R lingual gyrus, z = 4.19  ↑R cingulate gyrus, z = 4.19  *Recalling to-be-remember items:*  ↑L parahippocampal cortex (BA 30), z = 4.57  ↑L retrosplenial cortex (BA 31), z = 4.57  ↑L lingual cortex (BA 18), z = 4.57  ↑L cingulate cortex (BA 19), z = 4.57  ↑L fusiform gyrus (BA 37), z = 3.68 ↑L precuneus (BA 7), z = 3.34 ↑R lingual gyrus, z = 3.62  ↑R cingulate gyrus, z = 3.62  ↑R lingual gyrus, z = 3.51  ↑R cingulate gyrus, z = 3.51  ↑R thalamus, z = 3.40  ↑cerebellum, z = 3.64  *Both recalling and encoding:*  ↑L lingual gyrus (BA 18/19), z = 4.16  ↑L fusiform gyrus (BA 37), z = 3.68 |
| Raz et al. (2009) Experiment 1 | *π recall vs counting numbers:*  ↑medial frontal gyrus (Broadman’s Area (BA) 10)  ↑dorsolateral prefrontal cortex (BA 9)  ↓ventral anterior cingulate cortex (BA 24)  ↓ventromedial prefrontal cortex (BA 11, 47)  ↓posterior cingulate cortex (BA 31)  ↓R hippocampus  ↓L hippocampus  ↑lateral frontal cortex |
| Raz et al. (2009) Experiment 2 | *Early digits encoding:*  ↑motor associative areas and midline frontal regions (BA 16)  ↑visual association areas around the precuneate gyrus (BA 31)  ↑lingual gyrus (BA 19)  ↑fusiform gyrus (BA 23)  medial frontal (BA 10) ↑ and ↓ in the anterior cingulate gyrus and posterior cingulate gyrus (BA 24, 31) and hippocampus  *Digit recall:*  medial frontal (BA 10) ↑ and ↓ in the anterior cingulate gyrus and posterior cingulate gyrus (BA 24, 31) and hippocampus  *Later vs early encoding:*  ↓visual association cortex (BA 19, 23)  ↑anterolateral prefrontal region  ↑orbitofrontal cortex  *Early vs late encoding:*  ↑ association areas for visual processing and motor planning (e.g., BA 19, 16)  early ↑ in medial frontal cortex (BA 10) migrates later to regions of the dorsolateral prefrontal cortex (DLPFC) (BA 9, 46, and 47) |
| Raz et al. (2009) Experiment 1 and Experiment 2 | absence of hippocampal ↑ for new encoding but not for old retrieval  *π recall vs random digit early encoding:*  ↑dorsolateral prefrontal cortex (BA9)  ↑medial frontal gyrus (BA 10) early learning  *π recall vs random digit late encoding:*  ↑medial frontal gyrus (BA 10)  *Volumetric measures:*  ↑R subgenual region of the cingulate gyrus |
| Engvig et al. (2010) | *Cortical thickening:*  ↑L lateral orbitofrontal cortex (r with source/recognition memory ratio)  ↑R lateral orbitofrontal cortex  ↑R insular cortex  ↑fusiform cortices (r with memory improvement) |
| Mallow et al. (2015) | *Mnemonist vs control:*  recall: d > 4, recall speed: d > 1.5  *Encoding:*  ↑L visual cortex (BA 18)  ↑L visual cortex (BA 19)  ↑R visual cortex (BA 18)  ↑L medial superior parietal cortex (BA 7)  ↑L middle temporal gyrus (BA 39)  ↑L retrosplenial cortex (BA 30)  ↑R retrosplenial cortex (BA 30)  ↑L parahippocampus  *Recall:*  ↑L anterior superior temporal gyrus (BA 7)  ↑R motor cortex (BA 30**)** |
| Fellner et al. (2016) | *EEG group MoL vs Pegs:*  d = 4.46, 95 % CI [2.86 , 6.06]  *fMRI group MoL vs Pegs:*  d = 2.61, 95 % CI [1.5 , 3.7]  *Whole brain:*  ↑L posterior cingulate cortex (BA 30)  ↑L parahippocampal gyrus (BA 36)  ↑R posterior cingulate (BA 30)  ↑L superior occipital gyrus (BA 19)  ↑L superior temporal gyrus (BA 22)  ↑L middle temporal gyrus (BA 39)  ↑R parahippocampal gyrus (BA 36)  ↑R middle temporal gyrus (BA 39)  ↑R superior temporal gyrus (BA 39)  *Whole brain positive SME: remembered > forgotten (successful memory encoding):*  ↑L parahippocampal gyrus (BA 35)  ↑L insula (BA 13)  ↑L caudate body  ↑L putamen  ↑L middle frontal gyrus (BA 6)  ↑L middle temporal gyrus (BA 22, 21)  ↑L superior temporal gyrus (BA 22)  ↑L middle temporal gyrus (BA 39)  ↑L superior temporal gyrus (BA 39)  ↑L inferior frontal gyrus (BA 44,13)  *Whole brain negative SME, forgotten > remembered (failed memory encoding):*  ↓L lingual gyrus (BA 18)  ↓L posterior cingulate gyrus (BA 30)  ↓R cuneus (BA 18)  ↓R inferior parietal lobule (BA 40)  ↓R superior parietal lobule (BA 7)  ↓R supramarginal gyrus (BA 40)  ↓R middle frontal gyrus (BA 10, 8)  ↓R superior frontal gyrus (BA 9)  ↓R insula (BA 13)  *EEG whole brain:*  Tp ↓ L MTL (spatial processing)  Tp ↓ R MTL (spatial processing)  Tp ↓ L MTL (memory formation)  Tp ↓ for processing in L anterior temporal lobe (peak fusiform gyrus)  Tp ↓ for memory formation in L lateral temporal lobe areas (peak middle temporal gyrus)  ABp ↑ for mnemonic processing and positive SMEs in occipito-parietal areas and R lateralized regions (peak superior parietal gyri) |
| Müller et al. (2017) | *Memory athletes vs control group:*  ↑Enlarged anterior hippocampus but not posterior hippocampus.  ↑Enlarged R hippocampus but not the ↓L hippocampus.  ↑R anterior hippocampus exhibited the largest group difference.  The memory athletes exhibit a strong relation between the R posterior hippocampus and the R caudate nucleus volume, both of these volumes predict their ranking.  Volume of the R posterior hippocampus and the R caudate nucleus that predict the world ranking.  Within the athletes, functional connectivity from R anterior hippocampus to both the R posterior hippocampus and R caudate nucleus predicted the world ranking position.  The R anterior hippocampus is significantly correlated to both the R posterior hippocampus and the R caudate nucleus. |
| Dresler et al. (2017) | *Training effect:*  after 6 weeks of training MoL group n^2^ > 0.4 each  after 4 months follow-up η^2^ = 0.39  mnemonic training elicited changes in brain network organization that significantly resembled the network connectivity patterns that distinguish memory athletes from controls  *Connectivity between major hubs:*  ↑medial prefrontal cortex + ↑R dorsolateral prefrontal cortex + ↑L parahippocampal gyrus, ↑L and ↑R retrosplenial cortex, posterior cingulate cortex, and R angular gyrus  *Default mode network, medial temporal lobe and visual network connectivity:*  Resting State Network Dynamics: The mnemonic training condition, similarity with athlete-control connectivity patterns was significantly larger for between- versus within- network connectivity. Hence, the observed effect was mainly driven by between- rather than within-network connectivity patterns during task-free baseline rest.  Brain Network Connectivity during Encoding: For the mnemonic training condition, similarity with athlete-control con- nectivity patterns was significantly larger for within- versus between-network connectivity Hence, in contrast to the task-free resting state, the similarity effect was driven by within- rather than between-network connectivity pat- terns during task. |
| Wagner et al. (2021) | *Encoding MTG vs AC – Interaction contrast:*  ↑L superior frontal gyrus, z = 4.68  ↑L precentral gyrus, z = 4.5 ↑L inferior frontal gyrus, z = 4.48  ↑L angular gyrus, z = 4.32  *Encoding MTG vs PC – Main effect of session S1>S2:*  ↑L inferior frontal gyrus, z = 4.64  ↑L superior frontal gyrus, z = 4.43  ↑L middle frontal gyrus, z = 4.41  ↑L angular gyrus, z = 4.02  *Temporal order recognition*:  ↓R posterior parahippocampal and L and R retrosplenial cortex, z = –4.54  ↓L lateral superior parietal gyrus, z = –5.31  ↓R lateral superior parietal gyrus, z = –6.01  ↓posterior parahippocampal cortices  ↓L retrosplenial cortex  ↓R retrosplenial cortex  ↓precuneus  *Training-related activation decreases during temporal order recognition are associated with better free recall performance after 4 months:*  ↓L fusiform gyrus, z = –5.06  ↓R precuneus, z = –5.18  ↓L angular gyrus, z = –4.75  ↓hippocampus  ↓posterior parahippocampal region  ↓retrosplenial cortex  ↓thalamus  ↓R striatum  ↓L striatum  ↓medial prefrontal cortex  ↓orbitofrontal cortex  ↓precentral gyrus  *Increased hippocampal-neocortical coupling during post-task rest is related to memory consolidation in athletes and initially mnemonics-naïve participants after training:*  ↑coupling between the hippocampus and a L and R cerebellar region, z = 4.6  *Post-training stronger connectivity between:*  ↑hippocampus and the L and R lateral prefrontal cortex, L angular gyrus, the L hippocampus and parahippocampal cortex, L and R insula and R caudate nucleus, the brainstem and cerebellum |
| Liu et al. (2022) | *Effect of training:*  improvement across days η^2^ = 0.5  accuracy during scanning (baseline) *d* = 1.56, and the first practice session *d* = 1.14  *Hippocampal Contributions to Temporal Order Memory (SME) – Employment of strategy:*  ↑CA23DG  ↑parahippocampal gyrus  ↑L frontal medial cortex  ↑L orbital frontal cortex  *Hippocampal Contributions to Temporal Order Memory (SME) – Retrieval (more active during remembered than forgotten items):*  ↑CA1  ↑CA23DG  ↑L supramarginal gyrus  ↑R supramarginal gyrus  ↑L superior parietal lobule  ↑R superior parietal lobule  ↑L lateral occipital cortex  ↑R lateral occipital cortex  ↑L frontal pole  ↑R frontal pole  *Hippocampal Representations of Structured Event Sequences:*  The hippocampus subfields contained the representation of event structure: Whereas the CA1 contained information about the location identity, the CA23DG were sensitive to the sequential distance of the locations.  *Hippocampal Sequence Boundary Effects during Encoding:*  The similarity of neural representations is higher for within-boundary pairs than cross-boundary pairs. This effect was found in both CA1 and CA23DG.  *Hippocampal Temporal Context Reinstatement during Retrieval:*  We predicted that the brain regions containing representations of temporal context should show higher pattern similarity for short-distance pairs than long-distance pairs. We found this pattern in CA1. |
| Glasø de Lange et al. (2022) | *Effect of training on mitigating age-related decline in white matter (WM) microstructure*  ↑Inferior Longitudinal Fasciculus (ILF)  ↑Hippocampal Cingulum Bundle (HCB)  ↑Superior Longitudinal Fasciculus (SLF) |
| Belleville et al. (2022) | *Encoding:*  ↑L inferior frontal gyrus (BA9), z = 26.98 (might be artefact)  *Retrieval:*  ↓L temporal pole (BA38), z = –4.73  ↓R temporal pole (BA38), z = – 4.27  ↓L inferior frontal gyrus (p. triangularis) (BA32), z = –3.83  ↓L ACC (BA32), z = –3.55  ↓L superior medial gyrus (BA10), z = –3.54  ↓R middle orbital gyrus, z = –4.05  ↓R middle frontal gyrus (BA9,10), z = –3.99  ↓R superior medial gyrus, z = –3.52  ↓R posterior-medial frontal, z = –4.03  ↓L pallidum, z = –3.99  ↓L putamen, z = –3.44  ↓L rectal gyrus, z = –3.38  ↓L middle orbital gyrus, z = –3.66  ↓R inferior frontal gyrus (p. triangularis), z = –3.64  ↓R middle frontal gyrus, z = –3.36 |
| Notes: ↓ = decrease, ↑ = increase, z = z-score, BA = Brodmann area | |

####

#### THE MOL AND ITS EFFECTIVENESS IN YOUNG ADULTS ADDITIONAL INFORMATION

The to-be-remembered items were mostly concrete or high-imagery words, though some studies included complex texts and digits. Word lists were typically concrete nouns. Most studies assigned one item per location, but some unknowingly used multiple items per location, generally ranging from 1–6, with exceptions between 1–32 items and on average, 43.5 items were used across experiments. Locations ranged from 0–52, with an average of 10 locations and 2.77 items per location. Participants mainly created the locations themselves, though some were designed by researchers or controlled by administrators.

#### THE MOL AND ITS EFFECTIVENESS IN OLD ADULTS ADDITIONAL INFORMATION

The to-be-remembered items were mostly concrete or high-imagery words. Most studies assigned one item per location, but some unknowingly used multiple items per location, generally ranging from 1–6, with exceptions between 1–72 items with an average of 13 items per location and 1.6 items without the specific studies reusing the exact locations for a long time. On average, 21.6 items were used across experiments, with locations ranging from 12–30, with an average of 19.6 locations. Participants mainly created the locations themselves, though researchers designed a smaller half.

#### THE MOL’S COGNITIVE MECHANISMS UNDERLYING ITS EFFECTIVENESS ADDITIONAL INFORMATION

The to-be-remembered items were mostly concrete or high-imagery words. On average, 20 items were used across experiments, with locations ranging from 7 to 20, with an average of 16 locations and an average of 1 item per location. Experimentation mainly created the locations, though participants created a smaller half themselves.

#### THE MOL IN THE CONTEXT OF NEUROPSYCHOLOGY ADDITIONAL INFORMATION

The to-be-remembered items were mostly concrete or high-imagery words. On average, 36.3 items were used across experiments, with locations ranging from 10 to 20, an average of 14 locations, and an average of 2.8 items per location. Participants mainly created the locations themselves, and some locations were created by experimentation. The primary neuroimaging method was fMRI.
